# OsdR of *Streptomyces coelicolor* and the Dormancy Regulator DevR of *Mycobacterium tuberculosis* Control Overlapping Regulons

**DOI:** 10.1128/mSystems.00014-16

**Published:** 2016-05-03

**Authors:** Mia Urem, Teunke van Rossum, Giselda Bucca, Geri F. Moolenaar, Emma Laing, Magda A. Świątek-Połatyńska, Joost Willemse, Elodie Tenconi, Sébastien Rigali, Nora Goosen, Colin P. Smith, Gilles P. van Wezel

**Affiliations:** a Molecular Biotechnology, Institute of Biology Leiden, Leiden University, Leiden, The Netherlands; b Microbial Sciences, Faculty of Health and Medical Sciences, University of Surrey, Guildford, Surrey, United Kingdom; c Molecular Genetics, Leiden Institute of Chemistry, Leiden University, Leiden, The Netherlands; d InBioS - Centre for Protein Engineering, Université de Liège, Institut de Chimie B6a, Liège, Belgium; University of California, Berkeley

**Keywords:** Developmental control, *Streptomyces*, dormancy, stress response

## Abstract

Two-component regulatory systems allow bacteria to respond adequately to changes in their environment. In response to a given stimulus, a sensory kinase activates its cognate response regulator via reversible phosphorylation. The response regulator DevR activates a state of dormancy under hypoxia in *Mycobacterium tuberculosis*, allowing this pathogen to escape the host defense system. Here, we show that OsdR (SCO0204) of the soil bacterium *Streptomyces coelicolor* is a functional orthologue of DevR. OsdR, when activated by the sensory kinase OsdK (SCO0203), binds upstream of the DevR-controlled dormancy genes *devR*, *hspX*, and Rv3134c of *M. tuberculosis*. *In silico* analysis of the *S. coelicolor* genome combined with *in vitro* DNA binding studies identified many binding sites in the genomic region around *osdR* itself and upstream of stress-related genes. This binding correlated well with transcriptomic responses, with deregulation of developmental genes and genes related to stress and hypoxia in the *osdR* mutant. A peak in *osdR* transcription in the wild-type strain at the onset of aerial growth correlated with major changes in global gene expression. Taken together, our data reveal the existence of a dormancy-related regulon in streptomycetes which plays an important role in the transcriptional control of stress- and development-related genes.

**IMPORTANCE** Dormancy is a state of growth cessation that allows bacteria to escape the host defense system and antibiotic challenge. Understanding the mechanisms that control dormancy is of key importance for the treatment of latent infections, such as those from *Mycobacterium tuberculosis*. In mycobacteria, dormancy is controlled by the response regulator DevR, which responds to conditions of hypoxia. Here, we show that OsdR of *Streptomyces coelicolor* recognizes the same regulatory element and controls a regulon that consists of genes involved in the control of stress and development. Only the core regulon in the direct vicinity of *dosR* and *osdR* is conserved between *M. tuberculosis* and *S. coelicolor*, respectively. Thus, we show how the system has diverged from allowing escape from the host defense system by mycobacteria to the control of sporulation by complex multicellular streptomycetes. This provides novel insights into how bacterial growth and development are coordinated with the environmental conditions.

## Abstract (precis)

Dormancy is a state of growth cessation that allows bacteria to escape the host defense system and antibiotic challenge. Understanding the mechanisms that control dormancy is of key importance for the treatment of latent infections, such as those from *Mycobacterium tuberculosis*. In mycobacteria, dormancy is controlled by the response regulator DevR, which responds to conditions of hypoxia. Here, we show that OsdR of *Streptomyces coelicolor* recognizes the same regulatory element and controls a regulon that consists of genes involved in the control of stress and development. Only the core regulon in the direct vicinity of *dosR* and *osdR* is conserved between *M. tuberculosis* and *S. coelicolor*, respectively. Thus, we show how the system has diverged from allowing escape from the host defense system by mycobacteria to the control of sporulation by complex multicellular streptomycetes. This provides novel insights into how bacterial growth and development are coordinated with the environmental conditions.

## INTRODUCTION

**FIG 1:**
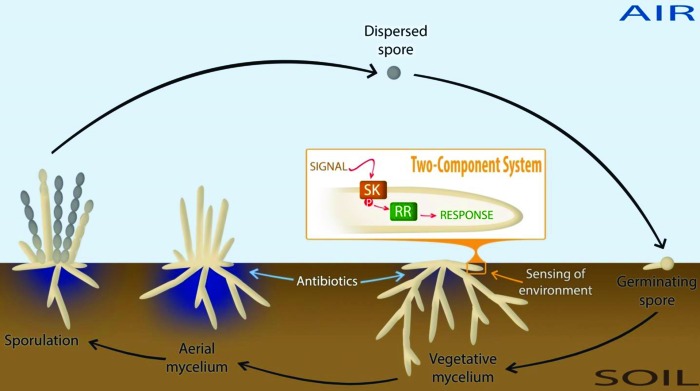
Life cycle of streptomycetes and environmental sensing of nutrients. The life cycle starts with the germination of spores, which grow out to form a branched network of vegetative hyphae. Under environmentally adverse conditions, such as nutrient depletion, streptomycetes initiate a complex developmental program whereby the vegetative mycelium serves as a substrate for a new so-called aerial mycelium. This stage of development usually corresponds with the production of secondary metabolites, such as antibiotics. Eventually, the aerial hyphae develop into chains of spores. Numerous regulatory networks exist in order to accurately sense and respond to the changing environmental conditions. Two-component systems (TCSs) couple the environmental stimulus (signal) of a sensor kinase (SK) to an adaptive response through phosphorylation of a cognate response regulator (RR), which exerts a regulatory response (usually through DNA binding), as illustrated in the orange box.

Complex natural habitats of bacteria call for rapid response systems to ensure adaption to often-changing environmental conditions. One prevalent mechanism that bacteria such as streptomycetes use to couple environmental stimuli to adaptive responses consists of a sensor kinase (SK) and a cognate response regulator (RR), which act as a two-component signal transduction system (TCS) (Fig. 1) (1, 2). Upon stimulation of the sensory domain of the SK by an external signal, the SK autophosphorylates itself prior to the transfer of the phosphate to a conserved His residue in the RR (1). Typically, the activity of the RR is mediated through DNA binding, although RNA and protein binding activities as well as catalytic activities have also been reported (1, 2).

Soil-borne bacteria, such as streptomycetes, have developed intricate sensory systems to detect nutrient availability and to initiate appropriate response mechanisms. Streptomycetes are industrially important organisms and produce a wide range of natural products, including over 50% of all known antibiotics (3, 4). The bacteria have a complex mycelial lifestyle (Fig. 1) and produce a branching network of vegetative hyphae, which are compartmentalized by cross-walls, making *Streptomyces* a rare example of a multicellular prokaryote (5). Under environmentally adverse conditions, such as nutrient depletion, streptomycetes initiate a complex developmental program whereby the vegetative mycelium serves as a substrate for a new so-called aerial mycelium. Eventually, the outer part of the aerial hyphae develops into chains of spores (6). In turn, the spores are able to survive periods of unfavorable conditions, such as anaerobiosis (for example, as a result of heavy rainfall). Though the model organism *Streptomyces coelicolor* is able to survive anaerobic conditions, anaerobic growth has not been reported for this microorganism (7).

The environmental conditions of a streptomycete’s natural habitat are ever-changing, and the complexity of the signals that are received and of the responses that are transmitted is reflected in the large number of TCSs, with the genome of *S. coelicolor* encoding 85 sensory kinases and 79 response regulators, with 67 known sensor-regulator pairs (8). One such pair is made up of the SK SCO0203 and the RR SCO0204. Unusually, SCO0203 has a second cognate RR in addition to SCO0204, namely, the orphan response regulator SCO3818 (9). The deletion of either RR gene was shown to enhance the production of actinorhodin, the blue-pigmented antibiotic of *Streptomyces coelicolor*. Although no biochemical evidence was provided, it was previously suggested that sensory kinase SCO0203 may be a direct orthologue of DosT, an SK from a well-studied TCS from the pathogenic obligate aerobe *Mycobacterium tuberculosis* (10).

In *M. tuberculosis*, gradual oxygen depletion is sensed by two SKs (DosT and DevS [alternatively known as DosS]) and induces a regulon controlled by the response regulator DevR (alternatively known as DosR), which consists of some 50 genes, including universal stress proteins (USPs), nitroreductases (which allow anaerobic nitrate respiration), redox proteins, and heat shock proteins (11). It is thought that this TCS regulates the escape from the host defense system by promoting dormancy to survive anaerobic conditions, and it is likely that this nonreplicating state plays a major role in the resistance of the bacilli to antibiotics (12, 13). An orthologous oxygen-sensing mechanism in streptomycetes may be essential for the sensing of oxygen levels in soil; under conditions of oxygen depletion, the appropriate response needs to be activated to ensure survival. Alternatively, under nutrient availability (and sufficient oxygen), vegetative hyphae form a very dense mycelium, where oxygen is locally depleted, and this depletion might be regulated via SCO0203/SCO0204.

In this work, we suggest that the TCS pair SCO0203/SCO0204 regulates a dormancy-related response in *S. coelicolor*. Major changes are seen in the global transcription patterns of genes related to stress and development in SCO0204 null mutants. The predicted core regulon of SCO0204, which revolves around the region from SCO0167 to SCO0219 in the *S. coelicolor* genome, contains many dormancy regulon-related genes and is conserved between SCO0204 and the dormancy regulator, DevR, of *M. tuberculosis*. We show binding of SCO0204 upstream of *M. tuberculosis* genes that are part of the DevR regulon as well as binding to the predicted binding site in *S. coelicolor*, including direct binding to developmental genes (which lack a predicted binding site). The locus tags SCO0203 and SCO0204 were named *osdK* and *osdR*, respectively, to highlight their function in response to oxygen availability, stress, and development.

## RESULTS

### Analysis of the two-component regulatory system OsdKR.

SCO0203 (OsdK) and SCO0204 (OsdR) form a two-component regulatory system (9) and are encoded by the *osdR-osdK* operon. OsdK has 41% and 42% amino acid identity (57% amino acid similarity) with DevS and DosT, respectively (see Fig. S1 in the supplemental material), and it was postulated as a possible ortholog of the dormancy sensory kinases of *M. tuberculosis* (14). Indeed, of the 18 amino acid residues required for oxygen sensing (15, 16), 15/18 residues of DosT and 12/18 residues of DevS are conserved in OsdK (Fig. S1). The interaction between the RR DevR and its target site is known in structural detail (17). OsdR and DevR share 61% amino acid identity (79% amino acid similarity) (Fig. S1 and S2), and comparison of the residues in the DevR and OsdR proteins revealed that 11 of the 13 residues implicated in DNA binding are conserved between DevR and OsdR (Fig. S2).

10.1128/mSystems.00014-16.6 Figure S1  Sequence alignment of the DNA binding domain of DevR with *Streptomyces* and *Mycobacterium* orthologues. The secondary-structure prediction was based on the crystal structure of DevR (PDB accession number 3C3W) and is shown on top. Conserved residues are shown in red, and boxes denote conservative substitutions. An upward arrow indicates residues involved in interactions with DNA; blue arrows indicate residues contacting nucleotide bases, and red arrows indicate residues making DNA phosphate oxygen contacts. Abbreviations: M. tuber, *M. tuberculosis*; Mmarin, *Mycobacterium marinum*; Msmeg, *Mycobacterium smegmatis*; Mvanba, *Mycobacterium vanbaalenii*; Shygro, *Streptomyces hygroscopicus*; Sscab, *Streptomyces scabies*; Scoeli, *S. coelicolor*; Sviola, *Streptomyces violaceoruber*; Ssirex, *Streptomyces* sp. strain Sirex. For accession numbers, see Materials and Methods. Download Figure S1, TIF file, 1 MB. Copyright © 2016 Urem et al. 2016 Urem et al. This content is distributed under the terms of the Creative Commons Attribution 4.0 International license.

10.1128/mSystems.00014-16.7 Figure S2  Alignment of SCO0203 (OsdK) and SCO0204 (OsdR). Multiple alignments of OsdK with DevS and DosT of *M. tuberculosis* (A) and OsdR with DevR of *M. tuberculosis* and NarL of *Pseudomonas aeruginosa* (B) were created with ClustalW (digits indicate the amino acid number). The different protein domains are indicated in italic. Amino acids conserved in at least 80% of the sequences are shaded (identical amino acids are in black, and amino acids with similar properties are in gray). Important amino acids of DosT and DevS are indicated beneath and above the alignment, respectively. GAF, GAF domain; DHp, dimerization and histidine phosphotransfer domain; CA, C-terminal catalytic and ATP binding domain; REC, receiver domain; HTH_LuxR, helix-turn-helix LuxR domain; ♦, phosphorylation site; ○, RR activation; **#**, H-bond network from iron to the surface; *, hydrophobic space surrounding a heme; **~**, contact with a propionate group heme; ●, ligand binding; ▲, surface crevice; **≈**, heme binding; ■, cavity next to a ligand binding pocket. Download Figure S2, TIF file, 2.4 MB. Copyright © 2016 Urem et al. 2016 Urem et al. This content is distributed under the terms of the Creative Commons Attribution 4.0 International license.

**FIG 2:**
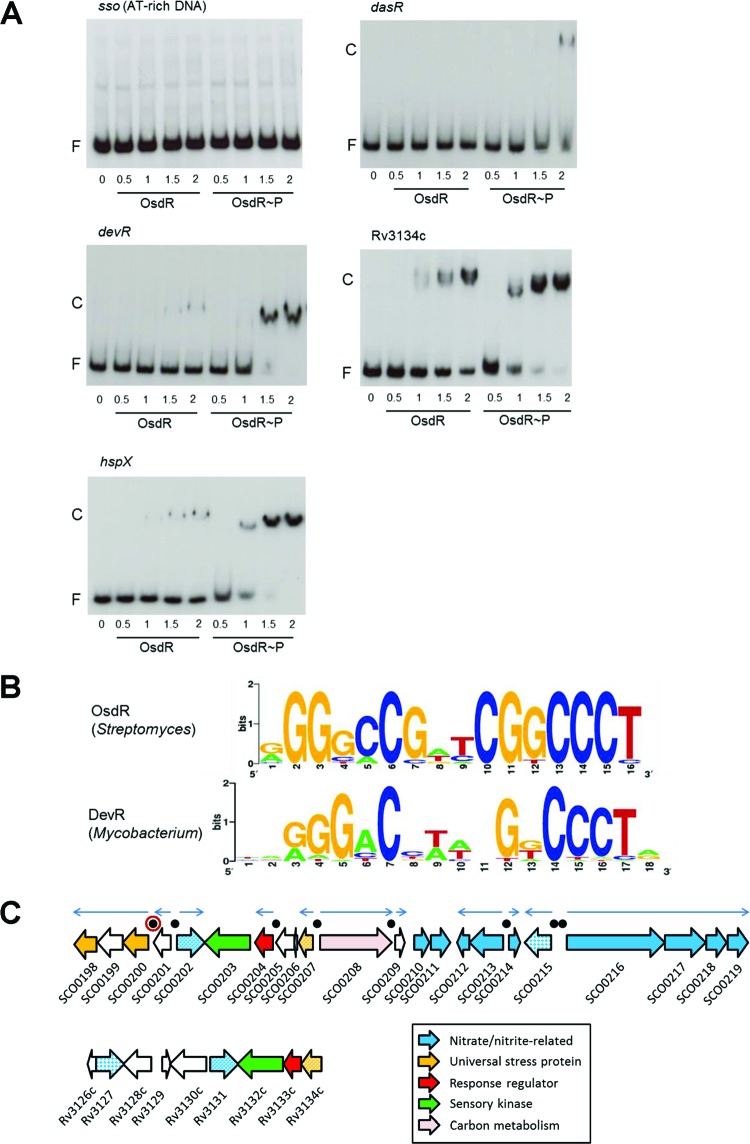
Binding site of OsdR (SCO0204) and comparison to that of *M. tuberculosis* DevR. (A) EMSAs using purified His_6_-tagged OsdR on known targets of DevR in *M. tuberculosis*. Both phosphorylated OsdR (OsdR~P) and nonphosphorylated OsdR (OsdR) were used in the assays. OsdR~P was obtained after AcP phosphorylation *in vitro*. Numbers on the horizontal axis refer to micromolar concentrations. F, free DNA fragment; C, complexes of DNA and protein. (B) Weblogo representation of *cis*-regulatory elements identified upstream of *osdR*, identified from the upstream regions of *osdR* orthologues in 12 *Streptomyces* species (see Materials and Methods). For comparison, the upstream regulatory element recognized by DevR (97) is presented. (C) Gene synteny between the loci around *osdR* in *S. coelicolor* (top) and *devR* in *M. tuberculosis* (bottom). Functional categories are given in the figure, and black dots indicate predicted OsdR binding sites, with that of *upsA* (SCO0200) surrounded by a red ring. Orthologues are presented in the same colors, and when multiple genes with similar functions are present, they appear in patterns.

To test whether *S. coelicolor* OsdR could bind to the recognition site of *M. tuberculosis* DevR, electrophoretic mobility shift assays (EMSAs) were performed. His_6_-tagged OsdK and OsdR were purified, and the sensory kinase OsdK was autophosphorylated using ^32^P-radiolabeled ATP and then used to transphosphorylate OsdR (Fig. S3). OsdR transphosphorylation could be achieved with autophosphorylated OsdK. However, OsdR readily lost its phosphosignal in the presence of OsdK, as previously observed for DevRS/DosT. Therefore, acetyl phosphate (AcP) was used as phosphor donor (18). As probes for EMSAs we used three mycobacterial promoters that are known targets of DevR (18), namely, the promoters for *devR*, Rv3134c (which is located upstream of *devR* and encodes a universal stress domain protein), and *hspX*, which encodes a latency-related heat shock protein. As negative controls, DNA fragments of the upstream region of *dasR* of *S. coelicolor* and AT-rich DNA from *Escherichia coli* were used. OsdR bound with low affinity to the DNA fragment encompassing the *dasR* promoter region, while no binding was seen when AT-rich *E. coli* control DNA was used (Fig. 2A). Interestingly, OsdR bound well to all probes for the mycobacterial target genes (Fig. 2A). Furthermore, similar differential affinities for the three fragments were observed as described previously for DevR in *M. tuberculosis* (18), with stronger binding upstream of Rv3134c and *hspX* than to the autoregulatory site of *devR*. Nonphosphorylated OsdR bound significantly less efficiently to the probes. Taken together, these data strongly suggest that OsdR and the dormancy regulator DevR recognize the same upstream regulatory elements, with phosphorylation by OsdK required to enhance DNA binding.

10.1128/mSystems.00014-16.8 Figure S3  *In vitro* autophosphorylation and transphosphorylation of OsdRK. OsdK was readily phosphorylated as shown by the large band in the lane “auto.” Using autophosphorylated OsdK, OsdR was transphosphorylated as shown by the presence of a band of phosphorylated OsdR and OsdK in the lane “trans.” However, there was significant phosphosignal loss observed (a decrease in band intensity over time). The reaction mixtures were run on 3.5% acrylamide gels in 0.5× Tris-borate-EDTA (TBE) buffer. Gels were dried and then subjected to autoradiography for analysis. Download Figure S3, TIF file, 0.1 MB. Copyright © 2016 Urem et al. 2016 Urem et al. This content is distributed under the terms of the Creative Commons Attribution 4.0 International license.

### *In silico* prediction of the OsdR regulon of *S. coelicolor.*

The OsdR consensus sequence was deduced by searching the upstream regions of *osdR* orthologues from 12 *Streptomyces* species for possible similar *cis*-acting regulatory elements using MEME (19). This identified a 16-nucleotide palindromic consensus sequence, 5′-AGGGCCGATCGGCCCT, which conforms well to the consensus sequence found in *M. tuberculosis* (Fig. 2B). The *S. coelicolor* genome was then scanned by PREDetector (20), using a position weight matrix (see Table S3 in the supplemental material) based on 12 predicted upstream elements as inputs. With a cutoff score of 8.0 for medium reliability (21), PREDetector identified putative binding sites for OsdR upstream of 27 transcription units. There was a total of 43 binding sites that may affect the transcription of 85 genes, which therefore may qualify as the direct OsdR response regulon (Table 1).

**TABLE 1:** Predicted binding sites for SCO0204 (OsdR) in *Streptomyces coelicolor*

Locus tag^a^	Function	Sequence	Position^b^	Score^c^	Cotranscribed gene(s)^d^	Function(s)
**SCO0204c**	OsdR, two-component response regulator	AGGGCCGGTCGGCCCC	−81	13.74		
**SCO0200c**	Universal stress protein	GGGGCCGACCGTCCCT	−100	12.49	SCO0199c/ SCO0198c	Zinc-deprived alcohol dehydrogenase, universal stress protein
SCO0215c	Nitroreductase family protein	AGGGCCGTCCGGCCCC	−99	12.24		
**SCO0208**	Pyruvate phosphate dikinase	CGGGCCGACCGGCCCT	−102	12.19		
			−158			
**SCO0207c**	Universal stress protein		−144	10.08		
			−88			
**SCO5979**	Enoyl-CoA hydratase	CGGGACCTTCGGCCCT	−80	11.62	SCO5980	Bifunctional hydroxylase, oxidoreductase
**SCO5978c**	Hypothetical protein		−68			
**SCO2637**	Secreted serine protease	AGGGCCGGTCGGCCTT	−53	11.27		
SCO7188c	Subtilisin-like secreted peptidase	GGGGACGATCGTCCCC	−47	11.2		
SCO0039	Hypothetical protein	AGGCCCGTTCCGCCCT	−132	10.86	SCO0040/ SCO0041/ SCO0042	Glycosyl transferase, integral membrane protein, hypothetical protein
SCO0038c	Sigma factor		−130		SCO0037c/ SCO0036c	Sigma factor, hypothetical protein
SCO0168	Crp-like regulatory protein	GAGGCCGGTCGGCCCT	−284	10.75		
		GGGGCCGACGGTCCCT	−36	9.21		
SCO0167c	Universal stress protein	AGGGACCTTCGGCCCC	−391	10.75		
			−114	10.73		
SCO0216	Nitrate reductase alpha chain NarG2	AGGGACCTTCGGCCCC	−53	10.73	SCO0217/ SCO0218/ SCO0219	Nitrate reductase beta chain NarH2, nitrate reductase delta chain NarJ2, nitrate reductase delta chain NarI2
SCO5410	Hypothetical protein	AGGGCAGGACGGCCCT	+36	10.6		
**SCO6041**	Protoporphyrinogen oxidase	GGGGCCGTCCGGCCCC	−51	10.57	SCO6042	Chlorite dismutase (oxygen-generating enzyme)
**SCO6040c**	Lipoprotein		−246		SCO6039c	Flavoprotein oxidoreductase, CoA disulfide reductase
SCO3431	EmrB/QacA subfamily transporter	GGGGCCGAACGGCCGT	+13	10.52		
SCO6164	Hypothetical protein with DksA/TraR family C4 zinc finger domain	GGGTCCGATCGGCCCG	−62	10.5		
SCO6163c	Sensor kinase		−334		SCO6162c	Two-component system response regulator
SCO0517	Possible Crp-like regulatory protein	GGGACCGACCGGCCCT	−248	10.49		
		AGGGCCGGCCGGCCCG	−268	10.46		
SCO3857	Nosiheptide resistance regulator	GGGCCCGTTCGGCGCT	−271	10.34		
SCO3856c	Peptidyl-prolyl *cis*-*trans* isomerase		−66			
SCO5251	Puromycin *N*-acetyltransferase	AGGGCCGTACGGCACC	−243	10.31		
SCO2347	Integral membrane protein	AGGGCCGAAAGTCCCG	−295	10.3		
SCO2348	Secreted protein		−221			
SCO0214	Pyridoxamine 5′-phosphate oxidase	GGGGCCATCCGGCCCT	−50	10.18		
SCO0213c	Nitrate-nitrite transporter protein		−252		SCO0212c	Hemerythrin cation binding domain-containing protein (oxygen transporting protein)
SCO0179c	Zinc-containing dehydrogenase	TGGGCCGGTCGGCCCC	−152	9.46		
SCO7021	Secreted protein	AGGCCCGAACGGCCCA	−94	9.4	SCO7022	Hypothetical protein SC1H10.11.
SCO4412	Regulatory protein	AGGGCGGAACGGCCGT	−261	9.34		
SCO0355	Conserved hypothetical protein SCF41.14	AGGGCTGACCGGCCCG	−81	9.2	SCO0356	Probable oxidoreductase

a SCO numbers in boldface were tested by EMSA.

b Position relative to the start of the gene.

c The cutoff score calculated using the PREDetector algorithm and based on the position weight matrix in Table S3 in the supplemental material.

d Genes known or predicted to be cotranscribed with the gene and therefore likely influenced by the regulatory element.

Eight binding sites were identified upstream of genes/operons in the vicinity of *osdR*, including *osdR* itself, controlling 20 of the 22 genes in the region between SCO0198 and SCO0219 (Fig. 2C). Comparison with the genomic region around *M. tuberculosis* *devR* revealed significant gene synteny (Fig. 2C). Of the 11 *S. coelicolor* genes for USP domain proteins, 8 are found in the genomic region between SCO0167 and SCO0021, and in *M. tuberculosis*, *usp* genes are part of the DevR regulon. SCO0213 to SCO0219 encode a nitrate transporter and nitrate reductase, which also prominently feature in the DevR regulon. When a lower cutoff score of 6.0 was used, PREDetector predicted a possible 27 elements in the regions SCO0167 to SCO0181 and SCO0198 to SCO0219.

### Specificity analysis of OsdR binding to the predicted regulatory element of *uspA.*

**FIG 3:**
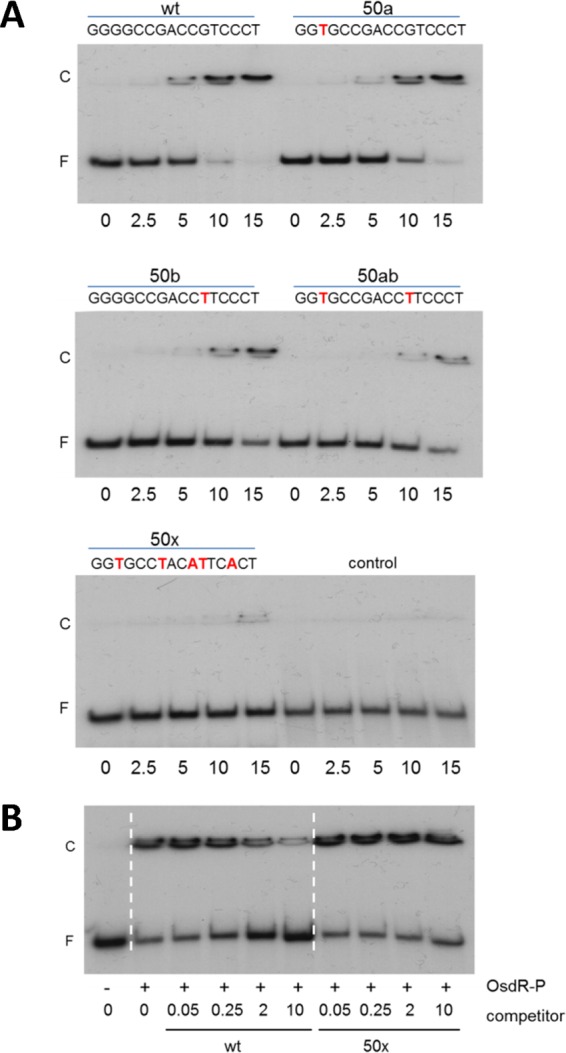
EMSAs with OsdR on a predicted *S. coelicolor* binding site. (A) Analysis of the OsdR binding site by mutation of highly conserved nucleotides in the *uspA* (SCO0200) binding site. Twenty femtomoles of a 50-mer DNA was incubated with increasing micromolar concentrations of OsdR~P. Replacements of the *upsA* binding site are indicated with red letters in the sequences; the 50-mer *dasR* fragment was used as a control. wt, wild type. (B) Competition assays using 10 µM protein and 20 fmol of labeled 50-mers centered on the *uspA* binding site. Increasing concentrations of the unlabeled competitor 50-mer were added, using either the wild-type *uspA* 50-mer or a mutated *uspA* 50-mer with 5 substitutions of highly conserved nucleotides (50x). − and + refer to the presence of phosphorylated OsdR (OsdR~P); competitor DNA is given in micromolar units. F, free DNA fragment; C, complexes of DNA and protein.

To investigate whether OsdR binds specifically to the predicted nucleotide sequence, a 50-mer probe of the upstream region of *uspA* (SCO0200), centered on the predicted binding site, was used as a probe (see Table S2 in the supplemental material). Indeed, AcP-phosphorylated OsdR (OsdR~P) bound well to the DNA fragment (Fig. 3A). Some retarded DNA remained in the wells of the gel, likely due to bridging, whereas each of the monomers of the OsdR dimer bound to a different probe rather than to the same site, which can result in long concatemers, as was observed for, e.g., NagR in *Bacillus subtilis* (22) and DasR in *S. coelicolor* (23).

We then designed four mutant 50-mer probes containing single mutations (designated 50a and 50b), a double mutation (50ab), or a quintuple mutation (50x) of the most conserved nucleotides of the binding site. In line with the predicted importance of the conserved nucleotide positions in the consensus sequence (Fig. 2B), nucleotide permutations significantly decreased the binding of OsdR to the probes, such that the single G→T substitution at position 3 (50a) and the G→T substitution at position 11 (50b) lowered binding efficiency by around 50%, which was further reduced by mutating both positions (Fig. 3A). Binding was abolished when five of the conserved nucleotides were mutated (50x). We also performed a competition assay with unlabeled DNA on the radiolabeled wild-type 50-mer *uspA* probe. Increasing the amount of the unlabeled wild-type *uspA* probe strongly inhibited binding by OsdR, while addition of unlabeled competitor DNA with 5 permutations in the binding site (50x) had no effect on OsdR binding (Fig. 3B). Taken together, these experiments provide conclusive evidence that OsdR specifically recognizes the predicted regulatory element.

### Verification of the regulon predictions by EMSAs.

**FIG 4:**
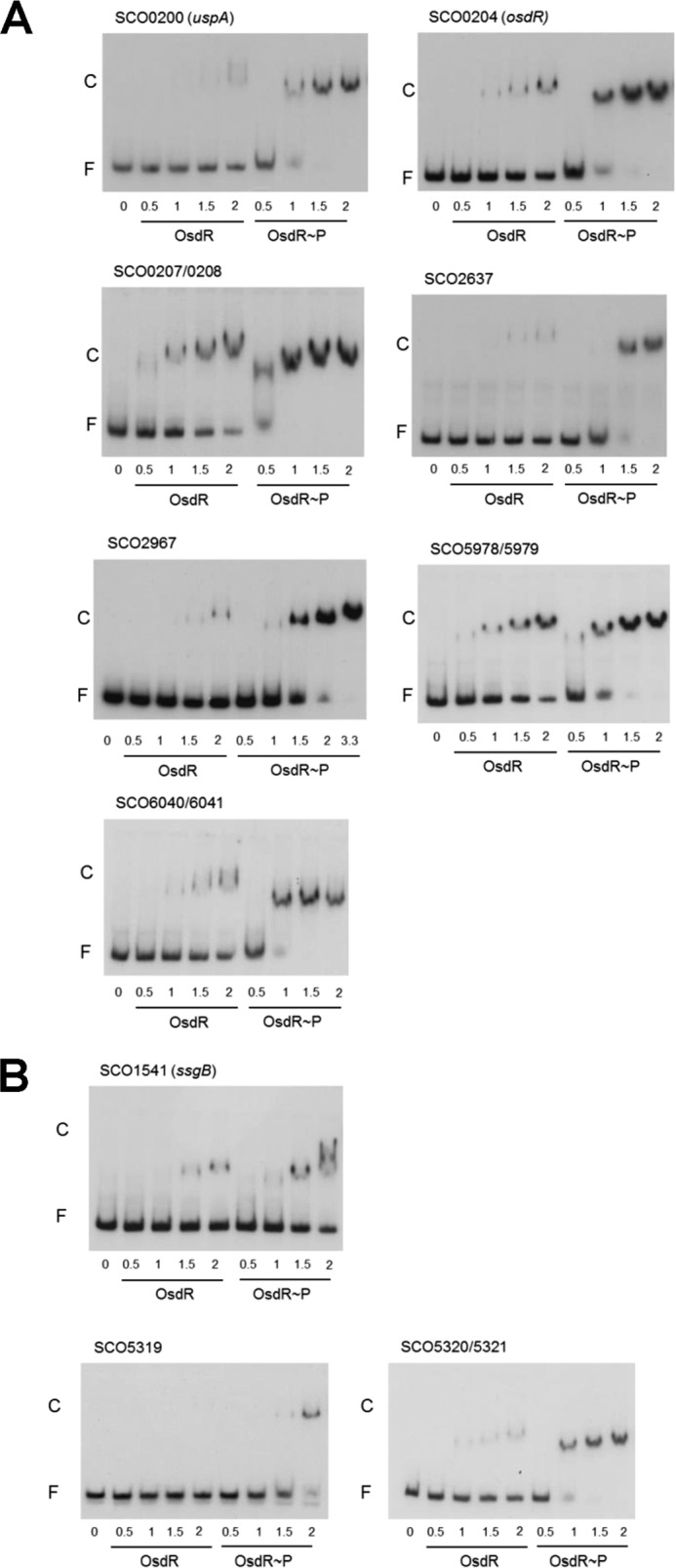
EMSAs with OsdR on selected *S. coelicolor* targets. (A) EMSAs were performed on DNA fragments harboring predicted binding sites upstream of the indicated genes. (B) EMSAs of *ssgB* (SCO1541) and *whiE* (SCO5319 and SCO5320-SCO5321). Concentrations of nonphosphorylated OsdR (OsdR) and phosphorylated OsdR (OsdR~P) are given in micromolar amounts. F, free DNA fragment; C, complexes of DNA and protein.

Next we tested DNA binding by OsdR to predicted targets using EMSAs of PCR-amplified DNA probes (Table S2). These were *uspA*, *osdR*, SCO2637 (for a serine protease), and SCO2967 (for a carboxypeptidase), and the intergenic regions between the divergent genes SCO0207 and SCO0208 (for another USP domain protein and pyruvate phosphate dikinase), SCO5978 and SCO5979 (for a hypothetical protein and an enoyl coenzyme A [enoyl-CoA] hydratase), and SCO6040 and SCO6041 (for a lipoprotein and a protoporphyrinogen oxidase). All the predicted binding sites were bound by OsdR, with most probes fully bound by OsdR~P (at 1 µM), except SCO2637, which was bound with 2-fold-lower affinity (Fig. 4A). This suggests that phosphorylation (by OsdK) leads to enhanced binding of OsdR to its binding sites. The combined predictions and EMSA data reveal some 50 likely OsdR target genes or gene clusters, of which at least 13 have orthologues that are controlled by DevR in *M. tuberculosis* (*osdR*, SCO0167, SCO0198, *uspA* [SCO0200], and SCO0207 and genes for nitrate reductase subunits).

### Transcriptional analysis of OsdR targets.

**FIG 5:**
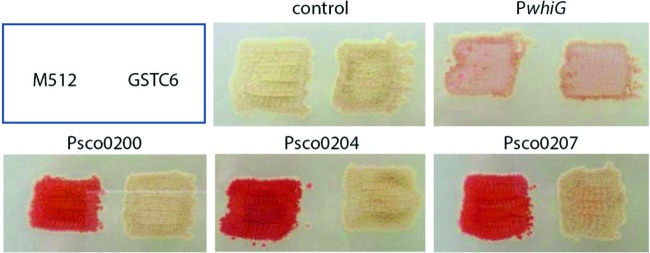
*In vivo* transcriptional analysis of OsdR targets. Promoter probing assays were performed for an analysis of the transcription of the promoters of *uspA* (SCO0200), *osdR* (SCO0204), and SCO0207 in the M512 *osdR* null mutant (GSTC6). As controls, the empty vector pIJ2587 and the *whiG* promoter were used.

To analyze the transcriptional control by OsdR, promoter probing was performed using the Red promoter probing system (see Materials and Methods) in the nonpigmented *S. coelicolor* strain M512 and the M512 *osdR* mutant derivative GSTC6. Promoter-probe vectors harboring the upstream regions of *uspA* (SCO0200), *osdR*, and SCO0207 were introduced into *S. coelicolor* M512 and the mutant GSTC6, and the promoter activity was analyzed, with as a control the empty vector or the vector with the *whiG* promoter, which is transcribed constitutively (the developmental control of the gene product σ^WhiG^ is governed primarily at the posttranslational level). While the empty vector did not show activity and *whiG* transcription was not affected by the deletion of *osdR*, P_SCO0200_, P_SCO0204_, and P_SCO0207_ were all active in M512 but poorly or not expressed in the mutant (Fig. 5), strongly suggesting that the genes are transcriptionally activated by OsdR.

### Global transcription profiling of the *osdR* null mutant by DNA microarray analysis.

**FIG 6:**
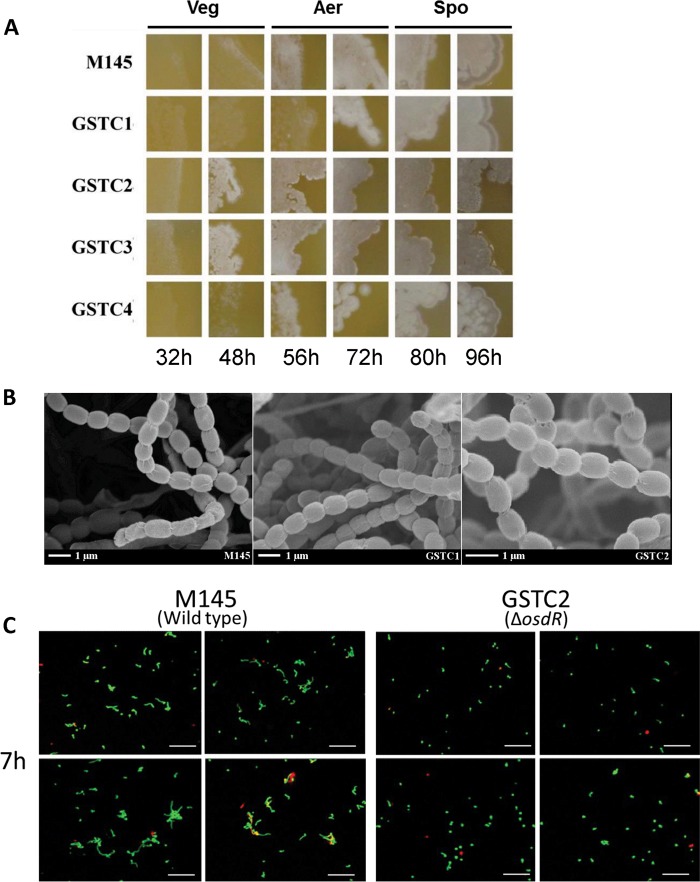
Phenotypic analysis of M145 OsdK and OsdR null mutants. (A) The different *osdK* and *osdR* mutants and their parent, *S. coelicolor* A3(2) M145, were grown on MS agar plates and monitored over time. Veg, vegetative growth; Aer, aerial growth; Spo, sporulation. (B) Phenotypic characterization of the *osdK* and *osdR* mutants and their parent, *S. coelicolor* M145, by cryo-scanning electron microscopy. Samples were prepared after 5 days of growth on MS. (C) Confocal fluorescence micrographs of germinating spores of *S. coelicolor* M145 and its *osdR* mutant GSTC2. Spores were inoculated onto MM agar and imaged after 7 h. Cells were stained with propidium iodide to identify dead cells (red) and with SYTO 9 green to identify living cells. GSTC1, M145 Δ*osdK*; GSTC2, M145 Δ*osdR*; GSTC3, M145 *osdR* in-frame deletion mutant; GSTC4, M145 *osdRK* double mutant.

Phenotypic analysis of the M145 *osdR* null mutants GSTC2 and GSTC3 as well as M145 Δ*osdK* (GSTC1) and M145 Δ*osdRK* (GSTC4) on MS medium indicated earlier formation of mycelial hyphae in the *osdR* mutants and accelerated sporulation and enhanced production of the gray spore pigment (Fig. 6A). In the absence of both OsdR and OsdK, this phenotype was not observed. High-resolution imaging by cryo-scanning electron microscopy revealed that the spores had a normal morphology (Fig. 6B). Observation of the spores with laser confocal microscopy indicated a strong delay in the germination of spores of the *osdR* null mutant compared to that of spores of the parental strain (Fig. 6C). Staining of dead and viable spores showed that this delay in germination in the GSTC2 mutant was not due to extensive accumulation of dead spores, as the proportions of viable/dying spores were comparable between *S. coelicolor* M145 and its *osdR* mutant derivative GSTC2.

To obtain a global overview of the effect of the deletion of *osdR* on transcription, microarray analysis was performed using RNA extracted from *S. coelicolor* M145 and its *osdR* null mutant GSTC2 grown on minimal medium (MM) agar plates overlaid with cellophane discs. Biomass was harvested at time points corresponding to vegetative growth (24 h), the onset of aerial growth (30 h), aerial growth (36 h), early sporulation (42 h), and sporulation (54 h) in the parental *S. coelicolor* M145. RNA from two independent biological replicate experiments was subsequently used as a template for cDNA synthesis/Cy3-dCTP labeling and subsequently hybridized onto oligonucleotide-based *S. coelicolor* whole-genome DNA microarrays (see Materials and Methods). By rank product analysis, a list of genes whose levels of expression were statistically significantly different was obtained at a percentage of false positives (PFP) of <0.01. With the additional cutoff of a minimum 2-fold change in the levels of transcription between the wild type and mutant, a list of over 800 genes whose transcription was significantly altered in the *osdR* null mutant was obtained (see Table S4 in the supplemental material). Classes of genes that were overrepresented were related to stress, anaerobic growth, and development. Notably, and as detailed further below, many of the genes that were differentially expressed between the wild type and *osdR* mutant had particularly strongly altered mRNA levels at 36 h. Suggestively, transcription of OsdR itself peaks at 36 h in wild-type cells, as shown in the present study and as established previously (see, e.g., reference 24).

### Stress-related genes and the chromosomal region around *osdRK.*

**FIG 7:**
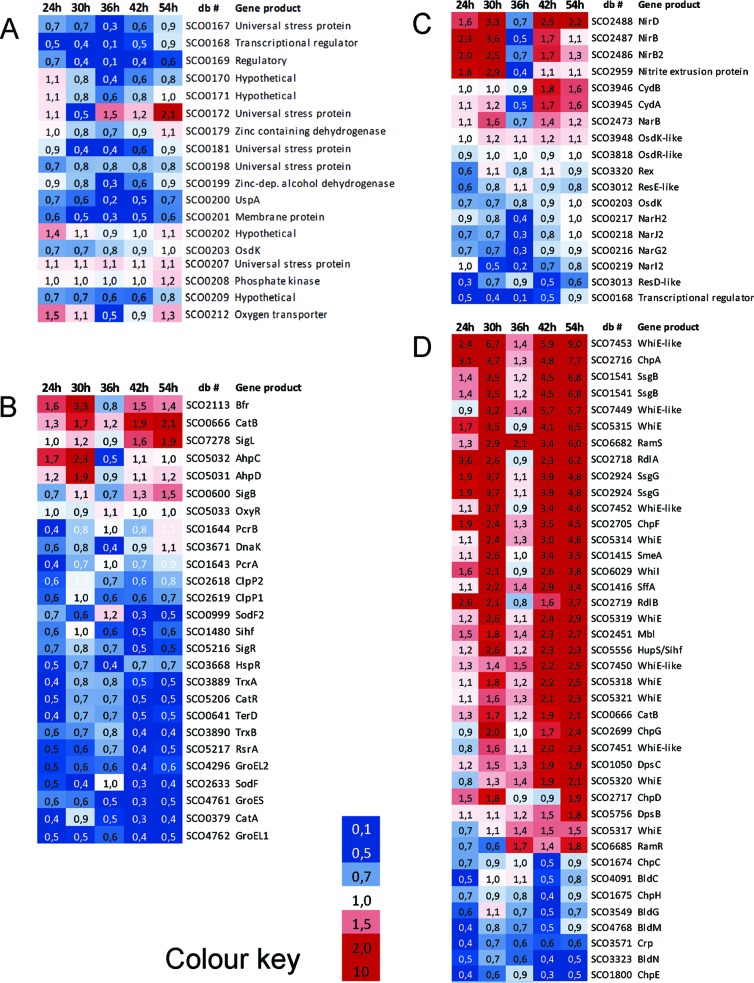
Heat maps of stress- and development-related genes differentially expressed between the *osdR* mutant and its parent, *S. coelicolor* M145. Transcription patterns (expressed as fold changes between the *osdR* mutant and the wild type) are presented for genes close to *osdRK* (A), stress-related genes (B), anaerobic-growth-related genes (C), and developmental genes (D). RNA was isolated from MM agar during vegetative growth (24 h), vegetative/aerial growth (30 h), aerial growth (36 h), aerial growth/early sporulation (42 h), and sporulation (54 h). Blue indicates downregulation (<0.5) and red indicates upregulation (>2.0) in the mutant; intermediate fold changes are represented in white. See Table S4 in the supplemental material. db #, database locus tag.

The majority of the genes encoding universal stress proteins are located in the vicinity of *osdK* and *osdR*, and several are predicted or proven members of the direct OsdR regulon (see above). Of these, SCO0167, SCO0172, SCO0181, and SCO0200 (*uspA*) were all downregulated at one or more time points in the mutant (Fig. 7A). The same was observed for the genes for the nitrate reductase system Nar2 (SCO0216 to SCO0219) at 36 h (Fig. 7C). *S. coelicolor* has three different nitrate reductases (Nar1 to -3) for anaerobic respiration, each active at different stages of development (25, 26). Genes for the two other nitrate reductase systems were not affected (see Table S4 in the supplemental material).

Deletion of *osdR* had a major effect on the transcription of many of the genes that were previously shown to be involved in stress management (27–31), such as the response to redox and (thiol) oxidative, osmotic, and temperature stress (Fig. 7B; see also Table S4 in the supplemental material). The σ factor gene *sigL*, which is involved in osmoprotection and oxidative stress (32), was upregulated, as was *catB*, but most of the stress-related genes were significantly downregulated. This included genes that in *B. subtilis* are part of the oxidative-stress response regulon (33), namely, *katA*, *trxA*, *trxB*, *msrA*, a *catR*/*perR*-like gene, and the genes for the oxidative-stress-related σ factor/anti-σ factor pair SigR/RsrA (34–36), as well as genes involved in protein degradation and folding, such as *clpP1* to *clpP2* (SCO2618 to SCO2619), *dnaK*, *hspR*, *groEL1*, *groEL2*, *groES*, genes encoding the proteasome (SCO1643 to SCO1644), and several cold shock genes. Zinc-related genes like those of the gene cluster for the zincophore coelibactin, were downregulated at all time points except 36 h, at which time levels of transcription were comparable between wild-type and *osdR* mutant cells (Fig. S4). Sufficient zinc is necessary for processes related to protein folding, redox balance, and oxygen stress (37–39). Similar changes in expression were observed for genes related to sulfur, cysteine synthesis, and thiol homeostasis (Fig. S4), which are involved in the management of (thiol) oxidative, redox, or osmotic stress (40, 41).

10.1128/mSystems.00014-16.9 Figure S4  Heat maps of genes related to zinc import (A), sulfur metabolism and thiol homeostasis (B), and nitrogen metabolism (C) that are significantly differentially expressed between the *osdR* mutant and its parent, *S. coelicolor* M145. RNA was isolated from mycelium grown on MM with 1% mannitol during vegetative growth (24 h), vegetative/aerial growth (30 h), aerial growth/early sporulation (42 h), and sporulation (54 h). Only genes with a PFP value of less than 0.010 and a fold change (Δ*osdR* expression/M145 expression) of more than 2.0 are presented. The levels of the fold changes are indicated with colors, as represented by the scale bar. Download Figure S4, TIF file, 0.3 MB. Copyright © 2016 Urem et al. 2016 Urem et al. This content is distributed under the terms of the Creative Commons Attribution 4.0 International license.

### Developmental control.

Major changes were observed in the global transcription profile of developmental genes, with a very distinctive pattern of upregulation of many sporulation genes in the absence of *osdR* at most time points, while early-developmental (*bld*) genes were downregulated at the same time points (Fig. 7D). Transcription of other genes, namely, *ssgB*, *ssgG*, *smeA-ssfA*, *chpADFG*, *rdlAB*, and *sapB*, all followed the same pattern, with a sharp peak at the onset of sporulation in wild-type cells and, instead, a steady increase in the mutant (see Table S5 in the supplemental material). SsgB and SsgG are members of the actinomycete-specific family of SsgA-like proteins (SALPs) (42) and determine the positions of septum sites during sporulation-specific cell division (43, 44). SmeA and SsfA are also involved in the control of septation as well as DNA segregation (45), and the *rdl* and *chp* genes encode the rodlin and chaplin proteins, respectively, which form amyloid-like structures to create a water-repellent hydrophobic sheath around aerial hyphae and spores (46–48). SapB is a lanthipeptide that acts as a signaling molecule for the onset of development (49, 50). The same transcriptional upregulation was observed for the *whiE* gene cluster for the spore pigment WhiE (51) and for the *whiE*-like gene cluster from SCO7449 to SCO7453, which also produces a spore pigment (52) (Fig. 7D; Table S4). The upregulation of sporulation genes correlates well to the accelerated development and enhanced pigmentation of *osdR* mutants (Fig. 6A).

Conversely, the early-developmental genes were downregulated in the *osdR* null mutant, including *bldC*, *bldG*, *bldM*, *bldN*, and *crp*, as well as *chpCEH*. The *crp* gene encodes the cAMP receptor protein that controls spore germination and early development (53, 54). The reduced expression of *crp* correlates with the observed strong delay in the germination of spores of the *osdR* null mutant (Fig. 6C). *bldG* encodes a developmental anti-σ factor antagonist that controls the activity of the stress σ factor σ^H^, *bldM* and *whiI* encode orphan response regulators that control complex developmental pathways (55), and *bldN* encodes a σ factor that is required for the transcription of, among other genes, the *chp* and *rdl* genes (56, 57). The downregulation of *chpCEH* contrasts with the upregulation of the other *chp* genes, which is the first time that such differential regulation has been observed. Interestingly, the *chpCEH* genes have been shown to belong to the early *chp* genes and are sufficient to support aerial development, while the other *chp* genes as well as *rdlAB* are produced significantly later during development (58). This is again consistent with the concept that OsdR represses sporulation and activates early-development processes.

### Differential expression at 36 h.

Interestingly, some 200 genes showed deregulated expression at the 36-h time point. These genes include 22 genes in the genomic region between SCO160 and SCO0220, as well as other members of the direct or indirect OsdR regulon that are involved in nitrogen metabolism and anaerobic respiration genes (e.g., *nar2*, *ureAB*, *nirB*, *glnD*, *glnII*, *glnK*, and *draK*), development (*whiE* and *whiE-*like genes, *ssgB*, *chp*, and *rdl*), stress management, etc. (see Table S5 in the supplemental material). These genes all showed a sharp rise or drop of transcription at 36 h in wild-type cells, with transcription recovering at 42 h, while such a sharp change in transcript levels was not seen in the *osdR* null mutant. The deregulated transcription of these genes in wild-type cells corresponds to a peak in *osdR* transcription at 36 h. A sharp peak in the expression of *osdR* toward the end of exponential growth in liquid cultures was observed by others, both in shake flasks (59) and in a fermentor (60). The transition from exponential to stationary phase roughly corresponds to the onset of aerial growth in surface-grown cultures. Interestingly, another peak in transcription was observed around 5 h after spore germination (61), which may correspond to OsdR’s control of early events.

### Verification by RT-qPCR and EMSAs.

**FIG 8:**
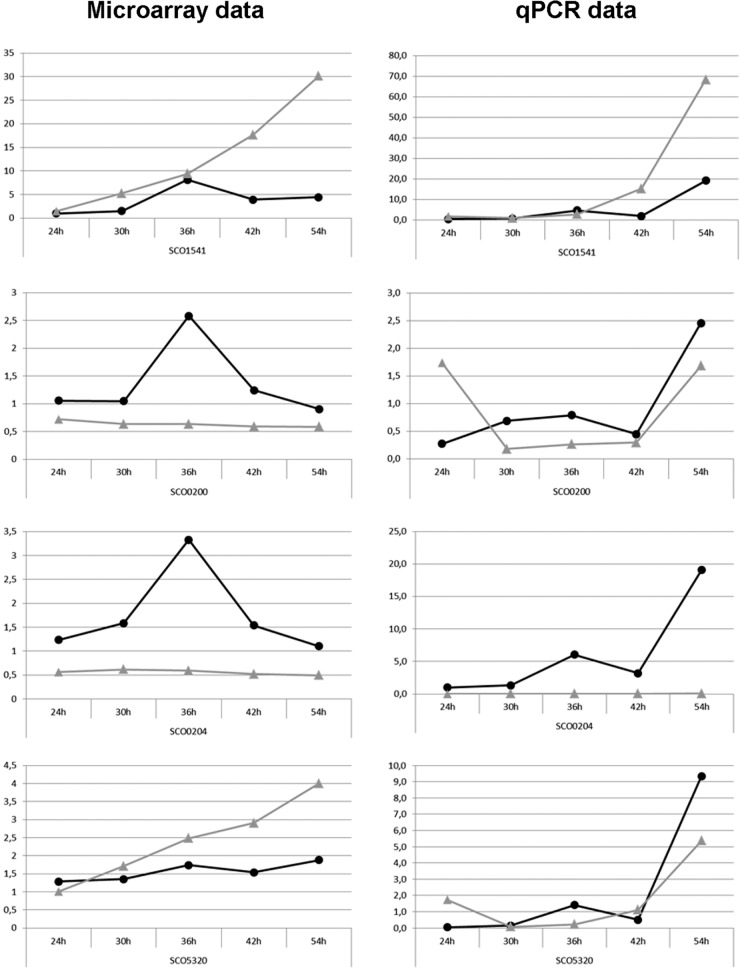
Microarray and RT-qPCR expression profiles of genes deregulated in the *osdR* mutant. RNAs for microarray analysis (left) and RT-qPCR (right) profiling were prepared from independent cultures. For time points, see Fig. 7. The expression profiles of the wild type (black circles) and the *osdR* mutant (gray triangles) from the microarray (left) and RT-qPCR (right) were compared. Genes of interest tested were SCO0200 (*uspA*), SCO0204 (*osdR*), SCO1541 (*ssgB*), and SCO5320 (*whiE*). See also Fig. S4 in the supplemental material. Note that the graphs are not at the same scale.

To corroborate the microarray data, reverse transcription-quantitative PCR (qPCR) analysis was performed on independent RNA samples isolated from the mycelia of *S. coelicolor* M145 and its *osdR* null mutant GSTC2 grown under the same conditions as those used to prepare RNA samples for microarray analysis. The results were normalized using *rpsI* (SCO4735) as the internal standard, and RNA obtained from mycelia of M145 grown for 24 h was used to normalize the results between the different qPCR runs. Similar trends in expression profiles were observed in both sets of transcript analyses (Fig. 8; see also Fig. S5 in the supplemental material). Expectedly, no *osdR* transcripts were detected in the *osdR* null mutant. The peak in the transcription of *osdR* after 36 h in wild-type cells, both in the microarray and in the qPCR data, again suggests that *osdR* plays an important regulatory role at this stage of the life cycle (Fig. 8). Downregulation of *upsA* (SCO0200) in the *osdR* mutant together with the binding of OsdR to the upstream regulatory element strongly suggests that *uspA* transcription is transactivated by OsdR. *ssgB* (SCO1541) transcription was higher in the mutant, which corresponds well with the accelerated development and enhanced spore pigmentation of GSTC2 (Fig. 6). The transcription of SCO5320 and SCO5321, which are part of the *whiE* gene cluster for the gray spore pigment, was increased at several time points (though *whiE* transcription also characteristically peaked at 36 h in the wild-type strain).

10.1128/mSystems.00014-16.10 Figure S5  Microarray and RT-qPCR expression profiles of genes deregulated in the *osdR* mutant. RNA was isolated for microarray analysis (left) and RT-qPCR (right) profiling from independent cultures grown on MM with 1% mannitol during vegetative growth (24 h), vegetative/aerial growth (30 h), aerial growth (36 h), aerial growth/early sporulation (42 h), and sporulation (54 h). The expression profiles of the wild type (black circles) and the osdR mutant (gray triangles) over time in the microarray data (left) and the RT-qPCR data (right) were compared. Genes of interest tested were SCO2637, SCO3323 (*bldN*), and SCO5321 (*whiE*). Download Figure S5, TIF file, 0.3 MB. Copyright © 2016 Urem et al. 2016 Urem et al. This content is distributed under the terms of the Creative Commons Attribution 4.0 International license.

While no regulatory elements were predicted upstream of *ssgB* or within the *whiE* cluster, EMSAs showed specific binding by phosphorylated OsdR to *ssgB* and to the intergenic region between genes SCO5320 and SCO5321 (Fig. 4B), while the promoters of SCO5319 and SCO5316 (the latter is not shown) were only weakly bound by OsdR *in vitro*. Considering the lack of binding of nonphosphorylated OsdR to the upstream regions of SCO5316 and SCO5319 and the weak binding of OsdR~P, it is unclear whether these two genes are directly controlled by OsdR *in vivo*.

## DISCUSSION

The two-component regulatory system (TCS) formed by OsdK (SCO0203) and OsdR (SCO0204) shows significant sequence similarity to the dormancy TCS in *Mycobacterium tuberculosis* (16). In this work, we show not only that the OsdR binding site conforms very well to the binding site for DevR in *M. tuberculosis* but also that OsdR recognizes the regulatory elements upstream of key genes of the *M. tuberculosis* dormancy regulon and with affinities similar to those of DevR. EMSAs established OsdR binding to short, 50-bp DNA sequences containing the predicted recognition site, and the specificity was validated by the decrease in binding upon changing of one or more nucleotides of the consensus sequence. Thus, the TCS formed by OsdK and OsdR is most likely orthologous to the dormancy control system DosT/DevS/DevR in *M. tuberculosis*. This is further supported by gene synteny, as many genes for USP domain proteins are in the vicinity of the TCSs in the respective organisms. Despite hundreds of millions of years of evolution, some 15 targets are conserved between the DevR-controlled dormancy regulon of *M. tuberculosis* and the regulon predicted to be controlled by OsdR in *S. coelicolor*. Most of these lie in the region around *osdR*, namely, SCO0167, *uspA*, *osdR*, SCO0207, SCO0215, and SCO0216 to SCO0219 (*narG2-narJ2*).

The sensory kinase OsdK activates its cognate response regulator, OsdR, by phosphorylation and enhances its DNA binding capability, as shown by the enhanced binding of OsdR~P in the EMSAs. Combined, the *in silico* predictions and *in vitro* validation by EMSAs indicate that around 50 genes or gene clusters are controlled directly by OsdR. Analysis of the transcriptional changes in the *osdR* null mutant by global transcription profiling revealed the deregulation of numerous stress-related genes, including numerous stress-related genes in the region around *osdR*. A distinctive pattern of deregulation of developmental genes was evident, with upregulation of sporulation genes (including *whiE*, *whiI*, *smeA-ssfA*, *rdlAB*, *ssgBG*, *ramS*, and the late *chp* genes) and downregulation of genes involved in early development (*bldC*, *bldG*, *bldM*, *bldN*, *crp*, and the early *chp* genes), which corresponds well to the observed accelerated development of *osdR* mutants. The transcriptional data suggest that OsdR controls a hinge point in development. This is perhaps best illustrated by the divergent transcription of the *chp* genes in the *osdR* mutant. It has previously been shown that the *chpCEH* genes are expressed earlier than the other *chp* genes and also that the ChpCEH proteins are sufficient to form the characteristic chaplin layer on the outside the aerial hyphae and spores and to support aerial growth. BldN was previously shown to control all of the *chp* genes (48, 56), which does not explain the difference in *chp* gene expression profiles. Our data show that in the *osdR* null mutant, transcription of *bldN* and *chpCEH* is reduced, while the other *chp* genes as well as *rdlAB* are upregulated. Therefore, we propose that fine-tuning of *chp* and *rdl* gene expression is maintained by OsdR.

Some of the differentially expressed genes that lack an obvious consensus sequence, in particular the *ssgB* and genes of the *whiE* gene cluster, were bound by OsdR *in vitro*. This indicates that the OsdR regulon may be larger than anticipated, and some members of the regulon may be controlled by so-called class II binding sites, in other words, sites that do not conform to the predicted consensus sequence site. Similar duality has been shown for many other functionally diverse global regulatory networks in bacteria, including those controlled by LexA (62) and Crp (63) in *E. coli*, Spo0A in *B. subtilis* (64), CtrA in *Caulobacter crescentus* (65), and Crp (66), GlnR (67), PhoP (68), and DasR (69) in *Streptomyces*. For *B. subtilis* Spo0A, some 15% of the total binding sites were not bound *in vitro* (64).

Extensive studies of the DosT and DevS signaling systems have indicated that, during hypoxia, the dissociation of oxygen from the SKs results in the transition from the inactive to the active states of these proteins. With the initial DevR hypoxic response mediated by DosT, which has a higher dissociation constant than DevS, the response is then maintained through DevS. Differences in the local structures surrounding a heme in either SK result in different oxygen affinities (15, 16). Additionally, ascorbic acid, nitric oxide, and carbon monoxide also induce the DevR regulon (70). NO has been shown to activate DosT under aerobic conditions by displacement of oxygen (71), while DevS acts as a redox sensor of the electron transport system and a decrease activates the SK under aerobic conditions (72). The similarity of the amino acid residues involved in signal recognition by DosT/DevS and OsdK suggests that oxygen is the major candidate as a sensory signal. Indeed, Daigle and colleagues showed that *osdR*, as well as many genes in the genomic region around *osdR*, were strongly upregulated in wild-type cells under both low-oxygen conditions and when cells were grown with sodium nitroprusside, an NO donor (10). Additional evidence for the oxygen stress-related function of OsdR was provided by a study of the proteomes of large versus small pellets (73), in which oxygen depletion within large pellets—which created local anaerobic conditions—resulted in the upregulation of various proteins expressed from the OsdR-controlled SCO0168-SCO0208 genomic region (26).

In liquid-grown cultures, where *S. coelicolor* forms large mycelial pellets (causing oxygen transfer problems toward the center of the clump [74]), and on solid-grown cultures (7), local oxygen depletion occurs. OsdKR-mediated oxygen sensing may well be responsible for the response to microaerobic conditions, during which the bacterium switches metabolism to meet the challenge of low oxygen. Still, streptomycetes cannot grow anaerobically, despite the presence of an arsenal of genes for enzymes associated with anaerobic metabolism (75). This has previously been referred to as the “anaerobic paradox.” This is exemplified by the surprising presence of three nitrate reductases in *S. coelicolor*, and our work shows that one of these is directly controlled by OsdR. Alternatively, *S. coelicolor* may undergo a state of dormancy as a means of survival. Indeed, while *S. coelicolor* cannot grow in oxygen-deprived soil, it is able to survive periods of anaerobiosis in which it remains dormant (7). Sporulation is a state of dormancy, and the fact that spore germination is significantly delayed in *osdR* null mutants without affecting spore viability (Fig. 6C) supports the notion that *osdR* controls this dormancy state. This delay was corroborated independently by imaging the germination of 500 spores of the wild type and the *osdR* mutant using light microscopy (not shown).

The transcriptional changes at 36 h of growth in the *osdR* null mutant are noteworthy, and while the results need to be worked out further, they may have major implications for the control of the switch from early- to late-developmental growth. Interestingly, such a clear transition in the global transcriptional profile of *S. coelicolor* has been reported previously, during growth in a fermentor. Distinctive sharp increases and decreases in the transcription of many genes were observed at this time point, and importantly, this includes several genes of the OsdR regulon, namely, genes in the nitrate reductase cluster adjacent to *osdR* (SCO0212-SCO0220), *bldN*, the *bldN*-controlled *chp* genes, and several other developmental genes (60). We observed a similar distinctive change in gene expression at 36 h in surface-grown cultures of wild-type cells, with many of the genes of the OsdR regulon, as well as *osdR* itself, showing expression in the wild-type strain different from that in the *osdR* null mutant. To some extent, the data from surface- and liquid-grown cultures can be compared, with many developmental genes upregulated in liquid-grown cultures at the time corresponding to the transition from exponential to stationary growth, suggesting that the phase of growth cessation in submerged culture is comparable to the onset of development (59). Our data provide a first indication that OsdR may play a major role in mediating a switch in gene expression during the transition from normal to developmental growth. The transcription of *osdR* also shows a peak almost immediately after germination (61), which suggests that OsdR may play a similar role during the transition from dormancy to early growth. Such a role of OsdR in mediating a rapid and global change in gene expression requires further investigation.

In summary, the TCS OsdKR of *S. coelicolor* is orthologous to the dormancy TCS system of *M. tuberculosis*, with OsdR regulating development and stress management in *S. coelicolor*. The signal activating this response system is likely related to stress, such as nutrient deprivation or hypoxic stress; however, this remains to be confirmed. OsdK also partners with SCO3818 (9), which adds an extra level of complexity. This also means that deletion of *osdR* may not completely inactivate the OsdK-based sensory system in *S. coelicolor*. The system may be even more complicated, as sensory kinase SCO3948 has a higher amino acid identity to OsdK than any other SK encoded by the *S. coelicolor* genome. Mutational and functional analysis followed by a system-wide analysis of the effects of all possible members of the control system on global gene expression should establish the level of cross talk between the two sensory systems and how they control the stress response of the complex soil bacterium *Streptomyces*.

## MATERIALS AND METHODS

### Bacterial strains and media.

The bacterial strains described in this work are listed in Table S1 in the supplemental material. *E. coli* strains JM109 and ET12567 were grown and transformed by standard procedures (76). *S. coelicolor* A3(2) M145 was the parent for the *osdK* (GSTC1), *osdR* (GSTC2 and GSTC3), and *osdRK* (GSTC4) null mutants. *S. coelicolor* M512 (M145 Δ*redD* Δ*actII*-ORF4 [77]) was the parent strain for the *osdR* null mutant GSTC6, and M512 and GSTC6 were the hosts for promoter probing experiments (78). Preparation of protoplasts, transformations, and conjugations were performed according to routine procedures (79). R5 medium was used for regeneration of protoplasts and MS medium (79) for the selection of mutants, for the preparation of spores, and for phenotypic characterization of mutants. To obtain mycelia for transcript analysis, strains were grown on minimal medium (agar plates with mannitol [1%, wt/vol] [79]).

10.1128/mSystems.00014-16.1 Table S1  Bacterial strain, plasmids, and constructs. Download Table S1, PDF file, 0.4 MB. Copyright © 2016 Urem et al. 2016 Urem et al. This content is distributed under the terms of the Creative Commons Attribution 4.0 International license.

### Preparation of gene knockout constructs.

Details for all plasmids and mutants are presented in Table S1 in the supplemental material. The gene replacement strategy was as described previously (80) and used the highly unstable vector pWHM3 (81), harboring around 1,500 bp of flanking region on either side of the gene targeted for deletion, and the genes of interest were replaced by the apramycin resistance cassette *aacC4* (82). PCRs were performed as previously described (83) with the oligonucleotides listed in Table S2. Plasmids pGWS378 and pGWS376 allowed gene replacement of *osdK* and *osdR*, respectively. To create an in-frame *osdR* deletion mutant (designated GSTC3), construct pGWS377, which carries only the flanking regions, was used for homologous recombination. Construct pGWS380 was designed for the construction of an in-frame *osdRK* double mutant (called GSTC4) by combining the upstream region of *osdR* (obtained from pGWS377) and the downstream region of *osdK* (obtained from pGWS378). GSTC6 (M512 Δ*osdR*) was created for promoter probing purposes using the same approach as for the *S. coelicolor* M145 *osdR* mutant.

10.1128/mSystems.00014-16.2 Table S2  Oligonucleotides. Download Table S2, PDF file, 0.5 MB. Copyright © 2016 Urem et al. 2016 Urem et al. This content is distributed under the terms of the Creative Commons Attribution 4.0 International license.

10.1128/mSystems.00014-16.3 Table S3  Position weight matrix for the OsdR binding site. Download Table S3, PDF file, 0.3 MB. Copyright © 2016 Urem et al. 2016 Urem et al. This content is distributed under the terms of the Creative Commons Attribution 4.0 International license.

10.1128/mSystems.00014-16.4 Table S4  Overview of microarray results. Download Table S4, PDF file, 0.1 MB. Copyright © 2016 Urem et al. 2016 Urem et al. This content is distributed under the terms of the Creative Commons Attribution 4.0 International license.

10.1128/mSystems.00014-16.5 Table S5  Overview of genes deregulated at 36 h. Download Table S5, PDF file, 0.8 MB. Copyright © 2016 Urem et al. 2016 Urem et al. This content is distributed under the terms of the Creative Commons Attribution 4.0 International license.

### Protein isolation, phosphorylation of OsdR, and electrophoretic mobility shift assays.

His_6_-tagged OsdR and OsdK were overexpressed from plasmids pET0203 and pET0204 in *E. coli* BL21(DE3) (9). The plasmids were a kind gift from Weihong Jiang (Shanghai Institutes for Biological Sciences, Chinese Academy of Sciences, Shanghai, China). Proteins were isolated using Ni-nitrilotriacetic acid (NTA) chromatography as described previously (84).

*In vitro* autophosphorylation of 30 pmol of OsdK was performed with ^32^P-radiolabeled ATP as described previously (9). For transphosphorylation of OsdR, 30 pmol of OsdK was autophosphorylated in 10 µl and incubated for 20 min at 30°C. Following a chill on ice, 80 pmol of OsdR was added. *In vitro* phosphorylation of OsdR for electrophoretic mobility shift assays (EMSAs) was achieved using the phosphor donor acetyl phosphate (AcP) as described previously (18). EMSAs with ^32^P-radiolabeled probes were performed as previously described (85).

The OsdR binding site was predicted and used to scan the *S. coelicolor* genome by PREDetector (20). This binding sequence was investigated by binding assay experiments with wild-type and mutated 50-mers of the predicted binding site upstream of SCO0200. The most-conserved nucleotides in the predicted binding sites (Table 1 and Fig. 2B) were identified, and single (50a, 50b), double (50ab), and quintuple (50x) substitutions were introduced (for 50-mer oligomers, see Table S2 in the supplemental material).

### Promoter probing.

Promoter probing experiments were performed using the *redD* system as described previously (78). The nonpigmented mutant *S. coelicolor* M512 lacks the pathway-specific activator genes *actII*-ORF4 and *redD* (77). When *redD* is transcribed from a promoter element cloned into the promoter-probe vector pIJ2587 (78), the RED biosynthetic pathway is activated, which can be monitored as a nondiffusible red pigment. Constructs for the *redD* promoter-probe system were created for the promoters of SCO0200, *osdR*, and SCO0207, using the *whiG* promoter as the control (Table S1). The promoter fragments were amplified by PCR, and EcoRI/BamHI-digested fragments were cloned into pIJ2587, resulting in the constructs pGWS345, pGWS1058, pGWS1059, pGWS1060 (for probing of *whiG*), SCO0200, *osdR*, and SCO0207.

### Transcript analysis.

RNA was isolated from *S. coelicolor* M145 (wild-type strain) and its *osdR* mutant GSTC2 by harvesting biomass from cellophane disks on MM with 1% mannitol after 24, 30, 36, 42, and 54 h of growth. Total RNA was isolated as described previously (85).

### Microarray analysis.

The quality and integrity of the RNA was tested with the Agilent 2100 Bioanalyzer (Agilent Technologies). The RNA was reverse transcribed into cDNA using Cy3-dCTP (http://www.surrey.ac.uk/fhms/microarrays/Downloads/Protocols/index.htm). Together with Cy5-dCTP-labeled *S. coelicolor* M145 genomic DNA as the common reference, the samples were hybridized onto 44,000 60-mer oligonucleotide microarray slides (86). The fluorescent signals on the slides were captured by an Agilent microarray scanner with Feature Extraction software (Agilent Technologies). Within-array normalization (global median) followed by cross-array normalization was performed in R (http://www.r-project.org) using Limma (version 2.5.0) (87, 88). Rank product analysis by means of the R packages RankProd (89) and RankProdIt (90) was applied to identify significantly differentially expressed genes (for which the probability of false prediction [PFP] value was <0.01) between the wild type and mutant at each time point. 

### RT-qPCR analysis.

For RT-qPCR analysis, cDNA was generated using the iScript Advanced cDNA synthesis kit (Bio-Rad Laboratories). RT-qPCRs were performed on 200 ng RNA with the iTaq universal SYBR green supermix (Bio-Rad Laboratories), using *rpsI* (SCO4735) as an internal control. Each reaction mixture was tested in triplicate and for normalization between different plates, with the 24-h wild-type sample as the reference. An average of all three measurements was used to calculate normalized expression.

### Microscopy.

Cryo-scanning electron microscopy was performed as described previously (91) with a JEOL JSM6700F microscope. Stereomicroscopy was done using a Zeiss Lumar.V12 stereomicroscope. Confocal laser-scanning microscopy was performed with a Leica TCS-SP2 microscope and Leica confocal software. Staining of dead and viable *Streptomyces* filaments and spores was performed as described previously (92) using the cell-impermeable nucleic acid stain propidium iodide (for dead cells) and the green fluorescent nucleic acid stain SYTO 9 (for live cells). Samples were examined at wavelengths of 488 and 568 nm for excitation and 530 nm (green) and 630 nm (red) for emission.

### Bioinformatics analysis.

Motif searching was performed with InterProScan (93) and Pfam 24.0 (94). Protein homology searches were performed using BLASTp (95). The comparative analysis of the upstream regions of OsdR orthologues was performed with MEME (19), using orthologues from *S. coelicolor*, *S. clavuligerus*, *S. scabies*, *S. ghanaensis*, *S. bingchengensis*, *S. cattleya*, *S. sviceus*, *S. viridochromogenes*, *S. griseoaurentiacus*, *Streptoccocus* sp. E14, *Streptoccocus* sp. TRS4, and *S. hygroscopicus*. The *S. coelicolor* genome was scanned for possible similar *cis*-acting regulatory elements using PREDetector (20). The consensus sequence for the predicted binding site of OsdR was visualized using WebLogo (96). The *M. tuberculosis* DevR binding site logo was created based on the primary DevR binding sites identified in reference 97.

### Accession numbers.

The microarray expression data have been deposited in ArrayExpress (with the accession number E-MTAB-4597). The GenBank nucleotide sequence accession number of *M. tuberculosis* DosT is P9WGK0, and that of DevS it is NP_217648. 
