# Supplementary figures and images for: OsdR of Streptomyces coelicolor and the Dormancy Regulator DevR of Mycobacterium tuberculosis Control Overlapping Regulons
Source: mSystems. 2016 May 3;1(3):e00014-16. doi: 10.1128/mSystems.00014-16 (PMC5069765; doi:10.1128/mSystems.00014-16)

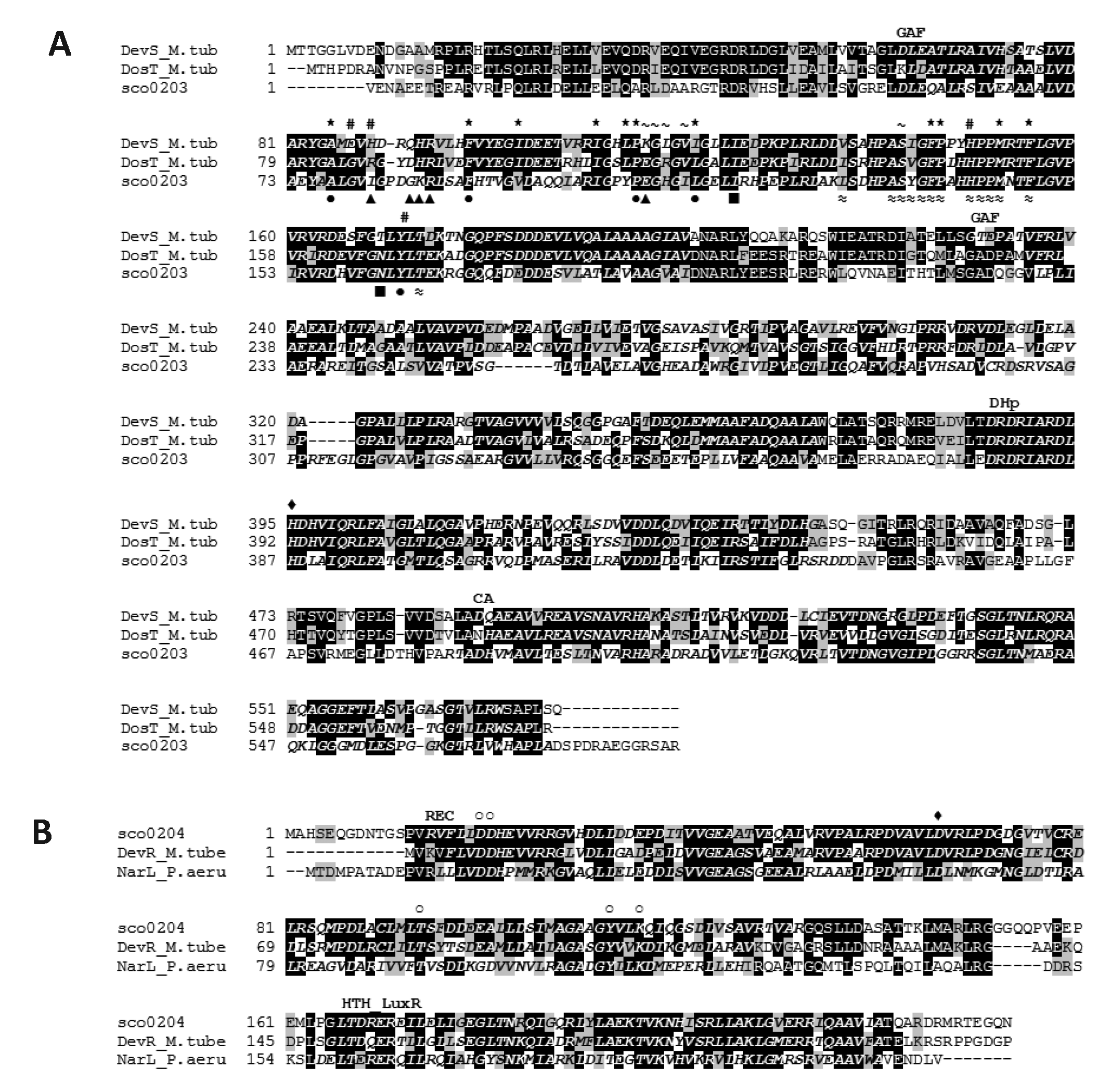

Supplement: Figure S1 [file sys001162018sf6.tif]

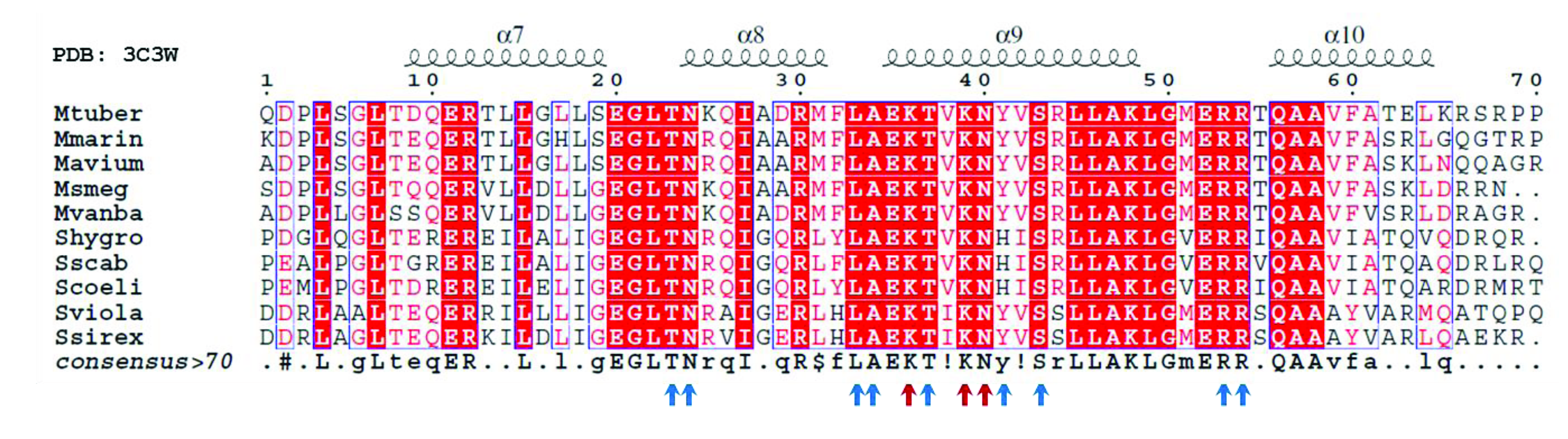

Supplement: Figure S2 [file sys001162018sf7.tif]

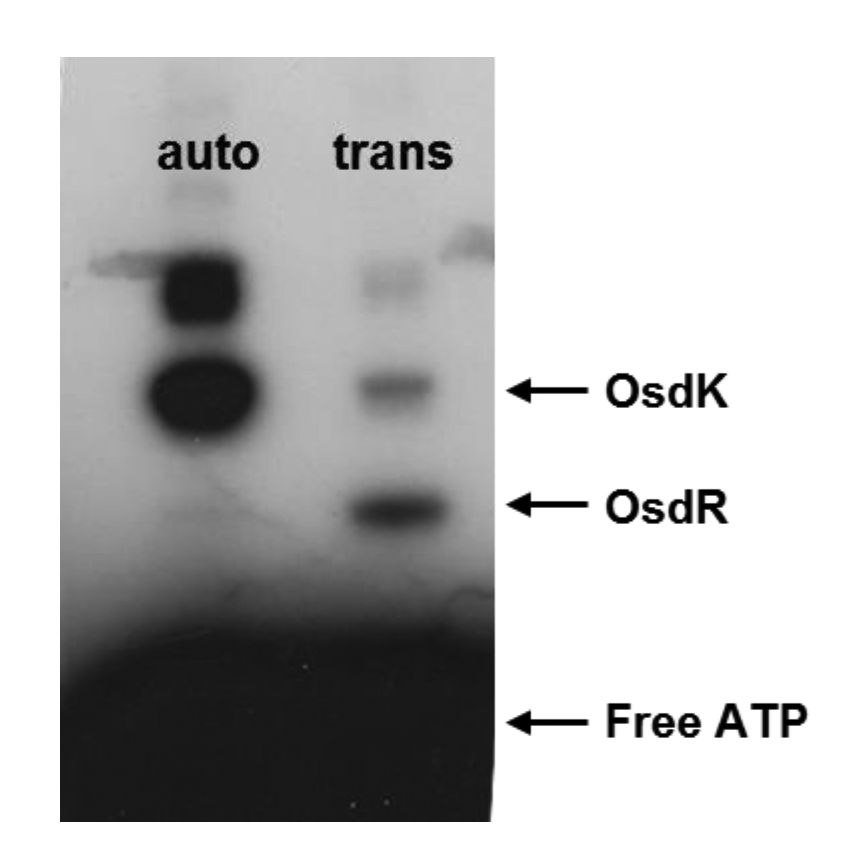

Supplement: Figure S3 [file sys001162018sf8.tif]

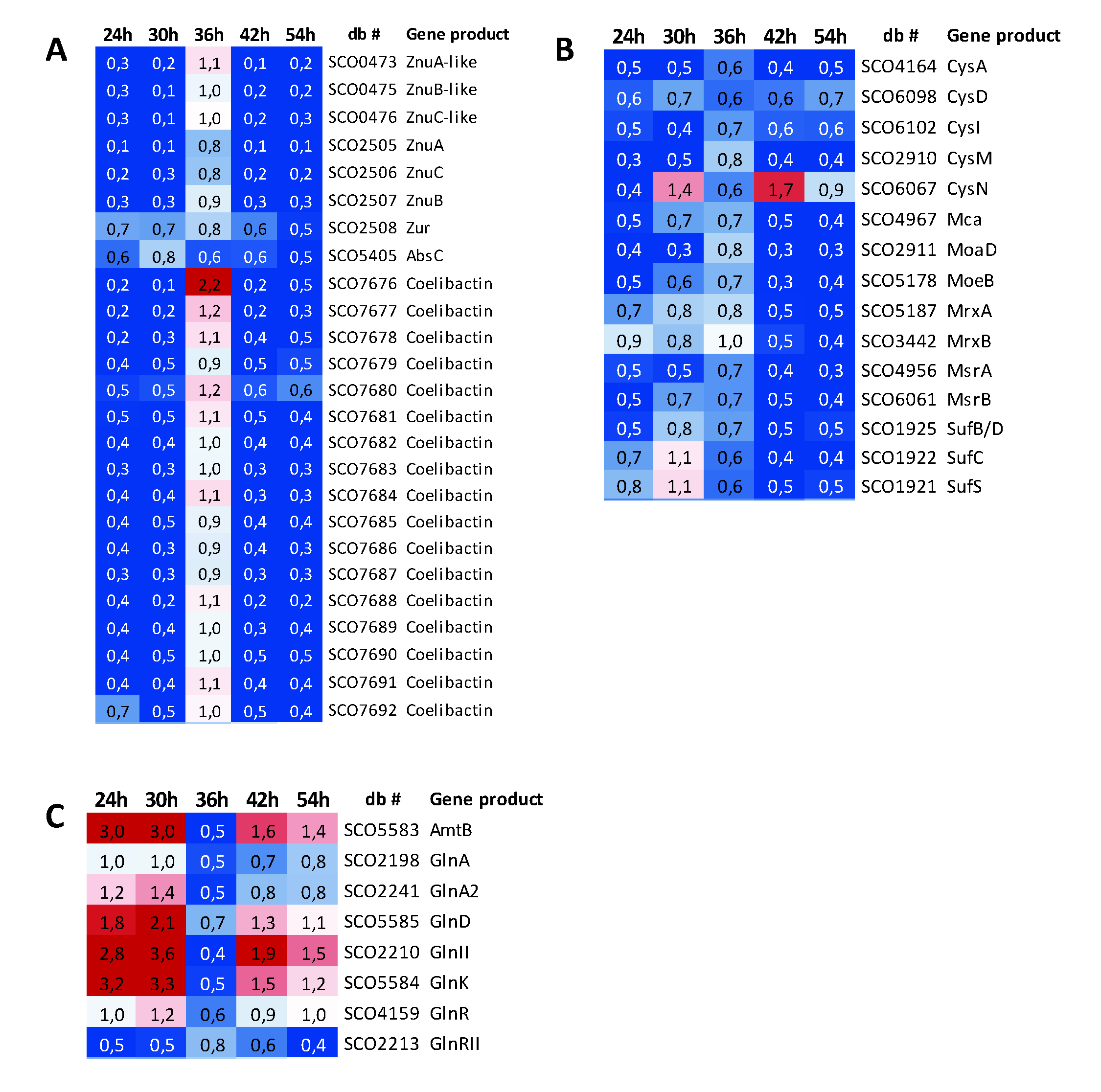

Supplement: Figure S4 [file sys001162018sf9.tif]

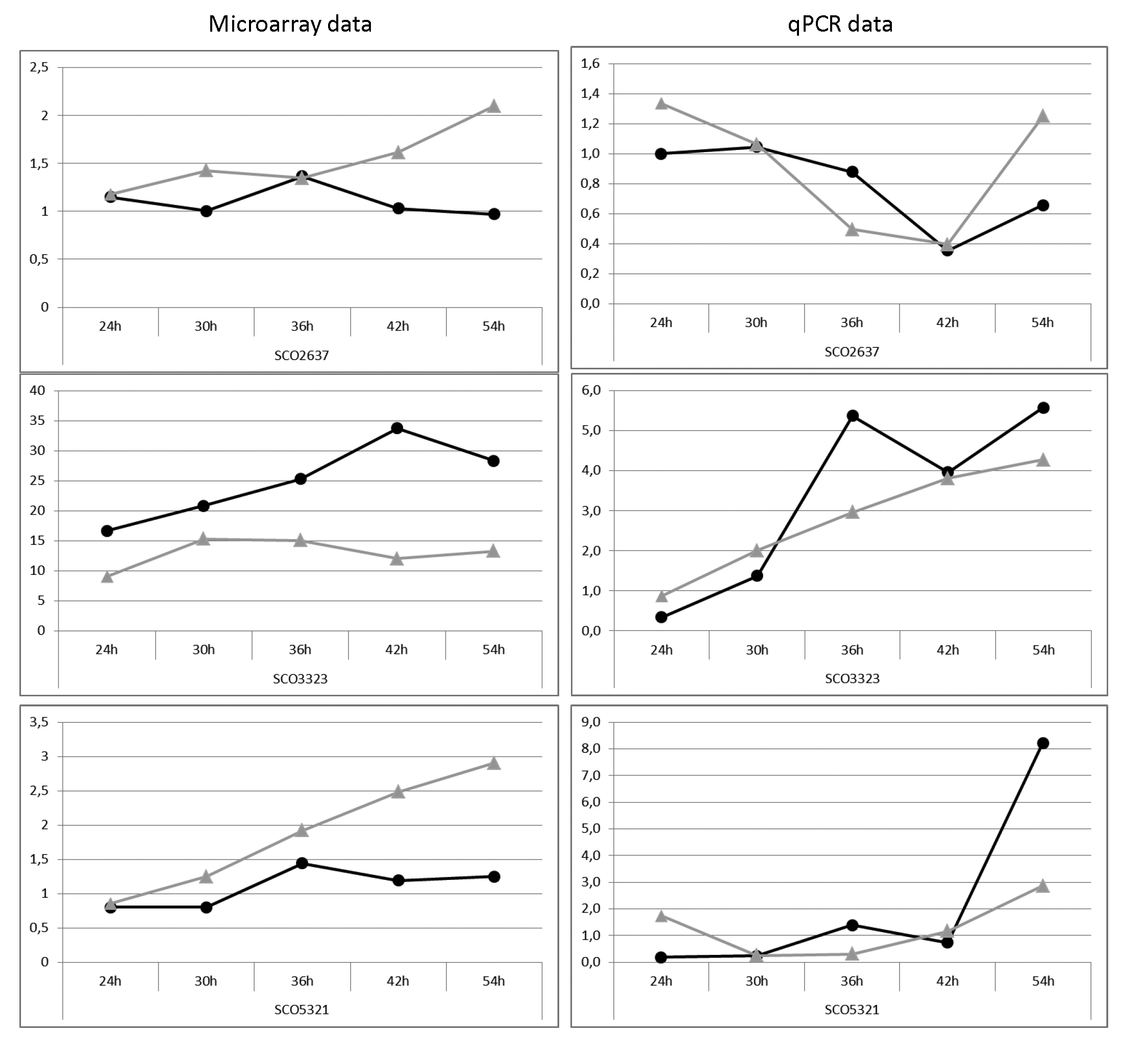

Supplement: Figure S5 [file sys001162018sf10.tif]
