# Supplementary material for: OsdR of Streptomyces coelicolor and the Dormancy Regulator DevR of Mycobacterium tuberculosis Control Overlapping Regulons
Source: mSystems. 2016 May 3;1(3):e00014-16. doi: 10.1128/mSystems.00014-16 (PMC5069765; doi:10.1128/mSystems.00014-16)
Supplement: Table S1 [file sys001162018st1.pdf]

Strains/plasmids	Genotype/description	Reference
Bacterial strain		
S. coelicolor A3(2) M145	SCP1⁻ SCP2⁻	(1)
M512	M145 Δ*actII-ORF4* Δ*redD*	(2)
GSTC1	M145 ΔSCO0203 (::*aacC4*)	this work
GSTC2	M145 ΔSCO0204 (::*aacC4*)	this work
GSTC3	M145 ΔSCO0204 (IFD[a])	this work
GSTC4	M145 ΔSCO0203/SCO0204 (IFD[a])	this work
GSTC6	M512 ΔSCO0204 (::*aacC4*)	this work
E. coli JM109	See reference	(3)
E. coli ET12567	See reference	(4)
Plasmid		
pWHM3	*E. coli - Streptomyces* shuttle vector (multi-copy in both hosts)	(5)
pHJL401	*E. coli-Streptomyces* shuttle vector with multiple copies in *E. coli* and 1-5 copies per chromosome in *Streptomyces*	(6)
pIJ2587	pHJL401 derivative with promoterless reporter gene *redD*	(7)
pGWS345	pIJ2587 harbouring the -395/+122 region relative to the start of whiG	this work
pGWS1059	pIJ2587 harbouring the -250/+38 region relative to the start of SCO0204	this work
pGWS1058	pIJ2587 harbouring the -211/+74region relative to the start of SCO0200	this work
pGWS1060	pIJ2587 harbouring the -341/+60 region relative to the start of SCO0207	this work
pGWS376	pWHM3 with 4 kb fragment for SCO0204 replacement by *aacC4*	this work
pGWS377	pWHM3 with 3 kb fragment for SCO0204 in-frame deletion	this work
pGWS378	pWHM3 with 3.4 kb fragment for SCO0203 replacement by *aacC4*	this work
pGWS380	pWHM3 with 2.7 kb fragment for SCO0203-0204 in-frame deletion	this work
pET0203	pET28b (Novagen) protein expression vector for SCO0203	(8)
pET0204	pET28b (Novagen) protein expression vector for SCO0204	(8)

REFERENCES

1. T. Kieser, M.J. Bibb, M.J. Buttner and K.F. Chater, Hopwood DA. Practical *Streptomyces* genetics. John Innes Foundation, Norwich, U.K., 2000.
2. B. Floriano and M.J. Bibb, Mol Microbiol **21:** 385-396, 1996.
3. J. Sambrook, E.F. Fritsch, and T. Maniatis, Molecular cloning: a laboratory manual, 2nd ed. Cold Spring Harbor laboratory press, Cold Spring harbor, N.Y., 1989.

4. D.J. MacNeil, K.M. Gewain, C.L. Ruby, G. Dezeny, P.H. Gibbons, and T. MacNeil, Gene **111:** 61-68, 1992.
5. J. Vara, M. Lewandowska-Skarbek, Y.G. Wang, S. Donadio, and C.R. Hutchinson, J Bacteriol 171:5872-5881, 1989.
6. J.L. Larson and C.L. Hershberger, Plasmid **15:** 199-209, 1986.
7. G.P. van Wezel, J. White, G. Hoogvliet, and M.J. Bibb, J Mol Microbiol Biotechnol **2:** 551-556, 2000.
8. W. Wang, D. Shu, L. Chen, W. Jiang, and Y. Lu, FEMS Microbiol Lett **294:** 150-156, 2009.
