# Supplementary material for: OsdR of Streptomyces coelicolor and the Dormancy Regulator DevR of Mycobacterium tuberculosis Control Overlapping Regulons
Source: mSystems. 2016 May 3;1(3):e00014-16. doi: 10.1128/mSystems.00014-16 (PMC5069765; doi:10.1128/mSystems.00014-16)
Supplement: Table S2 [file sys001162018st2.pdf]

Table S2. Oligonucleotides

Primers for cloning and RT-qPCR reactions

	Target *	Specification ^	Sequence(5'-> 3') #
Construction of mutants	SCO0203L	F-1162	GTACGAATTCTGGATCAGGTACGTCCTGGTGC
		R+16	CGATTGGCTGAGCTCATGAAGTTCTAGACACCCTGGCCTCGCGGGTCTC
	SCO0203R	F+1720	CTGCATAACCCTGCTTCGGGGTCTAGATCCCCGACAGGGCGGAAGGC
		R+2931	GTACAAGCTTACCCGACGCCGAAAGTCCGCACC
	SCO0204L	F-1439	GTACGAATTCGCGGTATCACCTTGGTGCACGTC
		R+16	CGATTGGCTGAGCTCATGAAGTTCTAGAGCTTCCCGTGTTGTCGCCCTG
	SCO0204R	F+670	CTGCATAACCCTGCTTCGGGGTCTAGACGGGACCGGATGCGCACCGAA
		R+2120	GTACAAGCTTAGCGGAGGCCGAAGATCGTCGAC
Verification of mutants	SCO0203	F-1253	CGACGTCCCCATGTACCGGAAGC
		R+3037	CCGAACCACGATGCGTGGACTGG
	SCO0204	F-1529	TGGAGTACGACATCGCGGTGG
		R+2241	ACGTGCGTGTCGAGCAGGCCCTC
	aacC4	F+783	TGCACGACATTGCACTCCAC
		R+219	TCTCGAGAATGACCACTGCTG
Promoter probing	pSCO0200	F-250	GTCAGAATTCTGATCGCGCTGGCCGCTGTCT
		R+38	GTCAGGATCCGGCGAGCCGTCGAGTCCTACGGT
	pSCO0204	F-211	GTCAGAATTCCCGCCAAGTACGACGCCCTG
		R+74	GTCAGGATCCACCACTTCGTGGTCGTCCAGGAG
	pSCO0207	F-341	GTCAGAATTCCGTGCACCAAGGTGATACCGCTC
		R+60	GTCAGGATCCACCGAGAAGTCCGGCCATGTC
RT-qPCR primers	SCO0200	F+387	CACCGAGACGCCGGTGATCCT
		R+461	ATGCCGGTGGGGTCCTTGAG
	SCO0204	F+331	GCCGGCTACGTCCTGAAGCA
		R+481	TGGCGTCCAGCAGCGACTGG
	SCO2637	F+1015	CAGGTCGGGTACTTCGGCAC
		R+1121	TTGGCACCGGCGGAGTTGTA
	SCO5320	F+359	AACCGCTGGCGACCTGCGTC
		R+489	GGTGAGGGCGTGCCGTACGA
	SCO5321	F+1017	CCACAACCTCGCCTGGAAGC
		R+1090	TGTCGTACGTGTCCAGCAGG
	SCO1541	F+205	GAGACCGTCGAGTGGGTCTT
		R+337	GGGAGCTGAGAGCGATGCAC
	SCO3323	F+313	CTCTACGACCAGTACAGCGA
		R+407	AGAAAGGTCTCGCTGGTGAG
	SCO4735	F+227	ACTACTTCCCGAACAAGGTGC
		R+306	GACGTCGTAGCGGTTGTCCAG

(continued on the next page)

Oligonucleotides and primers for EMSA experiments

	Target *	Location ^	Sequence (5'-> 3') #
50mers	pSCO0200 wt	50F	CCCATCACCCTGCGGGCAGGGACGGTCGGCCCCGTCCCGGGACCACAGGC
		50R	GCCTGTGGTCCCGGGACGGGGCCGACCGTCCCTGCCCGCAGGGTGATGGG
	pSCO0200 50a	50aF	CCCATCACCCTGCGGGCAGGGACGGTCGGCACCGTCCCGGGACCACAGGC
		50aR	GCCTGTGGTCCCGGGACGGTGCCGACCGTCCCTGCCCGCAGGGTGATGGG
	pSCO0200 50b	50bF	CCCATCACCCTGCGGGCAGGGAAGGTCGGCCCCGTCCCGGGACCACAGGC
		50bR	GCCTGTGGTCCCGGGACGGGGCCGACCTTCCCTGCCCGCAGGGTGATGGG
	pSCO0200 50ab	50abF	CCCATCACCCTGCGGGCAGGGAAGGTCGGCACCGTCCCGGGACCACAGGC
		50abR	GCCTGTGGTCCCGGGACGGTGCCGACCTTCCCTGCCCGCAGGGTGATGGG
	pSCO0200 50x	50xF	CCCATCACCCTGCGGGCAGTGAATGTAGGCACCGTCCCGGGACCACAGGC
		50xR	GCCTGTGGTCCCGGGACGGTGCCTACATTCACTGCCCGCAGGGTGATGGG
S. coelicolor targets	pSCO0200	F-250	GTCAGAATTCTGATCGCGCTGGCCGCTGTCT
		R+38	GTCAGGATCCGGCGAGCCGTCGAGTCCTACGGT
	pSCO0204	F-211	GTCAGAATTCCCGCCAAGTACGACGCCCTG
		R+74	GTCAGGATCCACCACTTCGTGGTCGTCCAGGAG
	pSCO0207	F-341	GTCAGAATTCCGTGCACCAAGGTGATACCGCTC
		R+60	GTCAGGATCCACCGAGAAGTCCGGCCATGTC
	pSCO1541	F-355	GCCGACGACGAAAACGCCGACC
		R+98	TCGGCCGTGTCGTACCGCAG
	pSCO2637	F-182	GTCAGAATTCTGTGTCCCCGATGGCGCATGG
		R+36	GTCAGGATCCGCCCGGTATCGGTTCGCGCT
	pSCO2967	F-218	GTCAGAATTCACGAAGCTGCCGCTCATCCTGG
		R+11	GTCAGGATCCCTCGCGAACACCAAGGTCAG
	pSCO5314	F-109	GCAGATGGCGCTGATCCGGGACC
		R+82	CGCTGTCGGACTCCGCGAACACC
	pSCO5316	F-139	GCGCGGCCTCATGGGGTCGAACT
		R+68	GCGGTGCGCTTCATCAGCGCCGA
	pSCO5319	F-190	TCCGTATGACGCCCCTGACCGA
		R+50	TTCGGGGCGATGTCGCCGAG
	pSCO5321	F-374	CATCCGTCGCTACGACGGGAGAG
		R+106	AGGTCGACAGGCCCACCAGGGAC
	pSCO5979	F-293	GTCAGAATTCCGTGTGCTCCAGCTCGGCCA
		R+50	GTCAGGATCCAGGTGCCGCCACTCGGGGGT
	pSCO6041	F-300	GTCAGAATTCTGCCGTCGTGCCTGCATCATGG
		R+56	GTCACTGCAGTGTCCGGGTTCCTGTTCCGGTGG
	pdasR	F+479	GCGCGAAGCGCTTCCCCGCCCTG
		R+675	GTCCTGGGAGTGCCGGGAGAGCATCAGCATG
M. tuberculosis targets	Rv3134c	R8	CCACCCGTGCGATAGGTGAGATTC
		R9c	CTCATCGACCGCCCACAACG
	devR	53R1	GTCAGCGCGGTTGTCGCGGAG
		R3	GACCTTTACCACCAGGGCACC
	hspX	hspXF2	TCTGAACGGCGGTTGGCAGACA
		hspXR	CGGGAAGGGTGGTGGCCATTTG

* target gene given as gene or SCO database number; nucleotide position is given relative to first nucleotide of the relevant gene.

p refers to promoter; 50mers denoted with '50'; primers for *M. tuberculosis* genes were described previously (Chauhan et al)

^ Forward (F) or reverse (R) primer; nucleotide position relative to first nucleotide of target gene.

\# Restriction sites underlined: GGATCC, BamHI; GAATTC, EcoRI; AAGCTT, HindIII; CTGCAG, PstI; GAGCTC, SacI; TCTAGA, XbaI.
