# Supplementary material for: OsdR of Streptomyces coelicolor and the Dormancy Regulator DevR of Mycobacterium tuberculosis Control Overlapping Regulons
Source: mSystems. 2016 May 3;1(3):e00014-16. doi: 10.1128/mSystems.00014-16 (PMC5069765; doi:10.1128/mSystems.00014-16)
Supplement: Table S3 [file sys001162018st3.pdf]

Table S3. Position weight matrix for the OsdR binding site.

OsdR Position weight matrix

(Minimum score : -39.68; Maximum score : 16.45)

	1	2	3	4	5	6	7	8	9	10	11	12	13	14	15	16
A	0.91	-2.48	-2.48	-2.48	0.25	-2.48	-2.48	1.12	-0.38	-2.48	-2.48	-2.48	-2.48	-2.48	-2.48	-2.48
C	-1.16	-2.48	-2.48	-1.16	0.77	0.97	-1.16	-2.48	-0.61	0.97	-2.48	-2.48	0.97	0.97	0.97	-1.16
G	0.39	0.97	0.97	0.77	-2.48	-2.48	0.87	-0.61	-2.48	-2.48	0.97	0.87	-2.48	-2.48	-2.48	-2.48
T	-2.48	-2.48	-2.48	-0.38	-2.48	-2.48	-2.48	0.91	1.58	-2.48	-2.48	-0.38	-2.48	-2.48	-2.48	1.80
