# Supplementary material for: OsdR of Streptomyces coelicolor and the Dormancy Regulator DevR of Mycobacterium tuberculosis Control Overlapping Regulons
Source: mSystems. 2016 May 3;1(3):e00014-16. doi: 10.1128/mSystems.00014-16 (PMC5069765; doi:10.1128/mSystems.00014-16)
Supplement: Table S4 [file sys001162018st4.pdf]

Supplementary Table S4: Overview microarray results

SCO #	Fold Change*					pfp	Protein Annotation
	24 h	*30 h*	*36 h*	*42 h*	*54 h*		
SCO0001	0,49	0,87	0,91	1,92	1,45	1,0966	Uncharacterized protein
SCO0008	1,69	1,84	2,44	1,07	2,26	0,0011	Uncharacterized protein
SCO0016	2,12	0,84	1,47	0,77	1,16	0,4867	Uncharacterized protein
SCO0036	2,48	1,49	1,35	1,03	1,32	0,0818	Uncharacterized protein
SCO0038	0,65	1,07	0,39	0,84	0,67	0,0824	Putative sigma factor
SCO0056	0,37	1,24	0,59	2,26	1,95	0,2743	Uncharacterized protein
SCO0057	0,74	0,46	1,02	1,26	1,51	0,4548	Uncharacterized protein
SCO0060	0,99	1,04	1,58	2,63	4,07	0,0020	Uncharacterized protein
SCO0097	0,41	0,82	1,89	1,15	1,50	0,9002	Putative integral membrane protein
SCO0112	0,26	1,05	0,91	1,85	1,49	0,2280	Uncharacterized protein
SCO0117	1,33	0,91	0,32	0,97	0,56	0,1500	Putative oxidoreductase
SCO0128	0,77	1,09	2,48	1,14	1,48	NA	Uncharacterized protein
SCO0129	2,05	1,40	1,72	1,17	1,59	0,0204	Uncharacterized protein
SCO0146	2,66	1,65	1,14	1,12	1,60	0,0314	Uncharacterized protein
SCO0156	0,73	0,75	1,20	2,18	2,05	0,1042	Uncharacterized protein
SCO0160	0,80	0,73	1,33	1,91	2,19	0,1442	Possible DNA-binding protein
SCO0162	1,02	0,74	0,46	0,76	1,20	0,0187	Uncharacterized protein
SCO0167	0,72	0,66	0,32	0,64	0,87	0,0059	Uncharacterized protein
SCO0168	0,49	0,43	0,10	0,54	0,90	0,0000	Possible regulator protein
SCO0169	0,69	0,43	0,13	0,37	0,55	0,0000	Uncharacterized protein
SCO0170	1,12	0,83	0,44	0,65	0,78	0,0658	Uncharacterized protein
SCO0171	1,10	0,82	0,63	0,81	0,98	0,2394	Nicotinate phosphoribosyltransferase (EC 6.3.4.21)
SCO0172	1,10	0,46	1,46	1,22	2,06	0,9469	Uncharacterized protein
SCO0174	0,75	0,72	0,43	0,86	1,30	0,0304	Putative DNA-binding protein
SCO0177	0,96	0,66	0,39	0,63	0,82	0,0195	Putative membrane protein SCJ1.26
SCO0181	0,91	0,44	0,39	0,56	0,94	0,0068	Uncharacterized protein
SCO0185	2,57	3,38	0,93	2,14	2,30	0,0000	Putative geranylgeranyl pyrophosphate synthase
SCO0186	2,18	3,62	1,07	2,69	2,82	0,0000	Putative phytoene dehydrogenase
SCO0187	1,61	2,58	1,20	2,19	2,24	0,0006	Putative phytoene synthase
SCO0188	2,24	3,43	1,31	3,22	2,89	0,0000	Putative methylesterase
SCO0189	2,05	2,80	0,89	2,01	1,42	0,0043	Putative dehydrogenase
SCO0190	1,98	3,16	0,87	1,86	1,37	0,0033	Putative methyltransferase
SCO0191	2,80	3,04	0,89	2,13	1,49	0,0008	Putative lycopene cyclase
SCO0194	1,16	2,20	0,83	0,86	1,92	0,1441	RNA polymerase sigma factor
SCO0195	1,42	2,56	0,99	1,19	2,61	0,0097	Putative lipoprotein
SCO0196	1,82	2,03	0,89	1,53	2,14	0,0242	Uncharacterized protein
SCO0199	0,95	0,79	0,32	0,64	0,93	0,0233	Putative alcohol dehydrogenase
SCO0200	0,68	0,61	0,25	0,48	0,65	0,0029	Uncharacterized protein
SCO0201	0,64	0,46	0,30	0,50	0,64	0,0008	Putative integral membrane protein SCJ12.13c
SCO0204	0,45	0,39	0,18	0,34	0,45	0,0000	OsdR, Two-component response regulator
SCO0214	1,45	0,53	0,33	0,46	0,74	0,0083	Uncharacterized protein
SCO0216	0,72	0,66	0,31	0,88	0,94	0,0275	Nitrate reductase alpha chain NarG2
SCO0217	0,91	0,80	0,44	0,89	1,01	0,2193	Nitrate reductase beta chain NarH2
SCO0218	0,69	0,72	0,33	0,80	1,01	0,0664	Putative nitrate reductase delta chain NarJ2
SCO0219	1,00	0,53	0,24	0,65	0,83	0,0091	Putative nitrate reductase delta chain NarI2
SCO0223	0,95	1,51	0,48	1,62	1,55	1,2206	Uncharacterized protein
SCO0224	2,22	1,31	1,02	1,00	1,12	0,4358	Uncharacterized protein
SCO0226	1,61	1,82	1,36	1,69	1,95	0,0085	Putative membrane protein
SCO0251	0,31	0,78	0,95	1,67	1,72	0,4366	Uncharacterized protein
SCO0252	2,07	1,16	1,21	1,11	1,18	0,2924	Putative oxidoreductase
SCO0355	0,90	1,09	1,00	2,39	1,69	0,0831	Uncharacterized protein
SCO0375	0,66	0,55	1,00	0,46	0,49	0,0764	Putative integral membrane transport protein
SCO0379	0,41	0,89	0,53	0,29	0,39	0,0016	Catalase (EC 1.11.1.6)
SCO0380	0,49	0,91	1,05	0,58	0,71	0,2589	Uncharacterized protein
SCO0381	0,42	0,78	1,05	0,70	0,63	0,2491	Putative glycosyl transferase
SCO0382	0,42	0,73	0,86	0,79	0,63	0,2125	UDP-glucose/GDP-mannose family dehydrogenase
SCO0383	0,44	0,71	0,82	0,63	0,56	0,0827	Uncharacterized protein
SCO0392	0,37	0,71	1,05	0,77	0,70	0,1683	Putative methyltransferase

SCO0393	0,39	0,80	0,82	0,70	0,59	0,0869	Putative transferase
SCO0415	0,64	0,80	2,14	1,61	2,83	0,0360	Putative membrane protei
SCO0426	2,66	1,37	1,15	1,13	1,27	0,0639	Uncharacterized protein
SCO0436	0,29	0,22	2,28	0,58	0,42	0,0009	RpmF2, 50S ribosomal protein L32-2
SCO0437	0,49	0,58	0,83	0,56	0,57	0,0728	Uncharacterized protein
SCO0446	1,02	1,72	0,83	2,06	1,67	0,1277	Uncharacterized protein
SCO0460	0,76	0,73	0,94	0,43	0,45	0,0868	Uncharacterized protein
SCO0463	0,47	0,46	0,89	0,51	0,38	0,0168	Uncharacterized protein
SCO0472	0,15	0,11	0,94	0,10	0,13	0,0000	Putative secreted protein
SCO0473	0,27	0,15	1,12	0,13	0,18	0,0000	Putative solute-binding lipoprotein
SCO0474	0,25	0,21	1,01	0,18	0,21	0,0004	Putative lipoprotein
SCO0475	0,30	0,13	0,95	0,17	0,19	0,0000	ABC transporter protein, integral membrane subunit
SCO0476	0,32	0,13	1,01	0,18	0,27	0,0000	ABC transport protein, ATP-binding subunit
SCO0477	0,41	0,34	1,13	0,28	0,32	0,0096	Uncharacterized protein
SCO0481	0,93	0,95	1,22	2,00	1,24	0,4732	Putative secreted chitin binding protein
SCO0502	0,50	0,88	0,95	2,32	0,82	0,6494	Putative membrane protein
SCO0509	1,02	1,47	1,22	2,50	2,53	0,0067	GlpK, Glycerol kinase 1
SCO0518	0,66	0,73	0,77	0,46	0,52	0,0662	Uncharacterized protein (Fragment)
SCO0530	0,97	1,13	0,47	0,98	0,93	0,7173	Putative transcriptional regulator
SCO0569	0,33	0,22	0,72	0,25	0,22	0,0000	RpmJ2, 50S ribosomal protein L36 2
SCO0570	0,08	0,12	0,72	0,14	0,11	0,0000	RpmG3, 50S ribosomal protein L33 3
SCO0591	1,06	1,78	1,26	2,42	3,29	0,0007	Lysozyme (EC 3.2.1.17)
SCO0598	0,39	0,76	1,19	0,91	0,88	0,5884	ArsJ, Putative anti anti sigma factor
SCO0607	0,73	1,09	1,30	1,25	2,13	0,1297	Hypothetical lipoportein SCF55.31
SCO0641	0,40	0,74	0,70	0,50	0,53	0,0228	Tellurium resistance protein
SCO0649	1,34	1,35	0,99	2,25	2,39	0,0063	Putative gas vesicle synthesis protein
SCO0650	1,91	2,21	1,20	2,86	3,15	0,0000	GvpA2, Probable gas vesicle structural protein 2 (GVP)
SCO0651	1,24	1,63	1,25	1,87	2,30	0,0089	Putative gas vesicle synthesis protein
SCO0653	0,88	0,99	1,43	2,07	1,99	0,1039	Uncharacterized protein
SCO0665	1,67	1,28	1,13	2,21	2,74	0,0034	Putative secreted protein
SCO0666	1,29	1,74	1,21	1,85	2,13	0,0191	Catalase (EC 1.11.1.6)
SCO0678	2,56	1,99	1,53	2,26	2,42	0,0000	UPF0337 protein SCO0678
SCO0679	1,54	1,79	1,14	2,69	3,00	0,0008	Uncharacterized protein
SCO0682	0,40	0,62	0,68	0,30	0,44	0,0077	Uncharacterized protein
SCO0683	0,51	0,74	0,94	0,49	0,70	0,1044	Uncharacterized protein
SCO0684	0,43	0,84	0,73	0,61	0,64	0,1165	Uncharacterized protein
SCO0685	0,47	0,81	0,73	0,61	0,70	0,1484	Uncharacterized protein
SCO0705	0,36	0,60	0,64	0,42	0,40	0,0047	Uncharacterized protein
SCO0721	1,08	1,33	0,94	2,04	1,51	0,2489	Putative glycosyl transferase
SCO0743	1,68	2,29	0,78	1,85	2,26	0,0041	Putative secreted protein
SCO0750	0,51	0,55	0,63	0,26	0,32	0,0006	Uncharacterized protein
SCO0753	0,34	0,81	1,44	0,77	0,98	0,2874	Uncharacterized protein
SCO0777	0,95	1,27	0,99	1,79	2,11	0,2010	Uncharacterized protein
SCO0778	1,56	1,67	1,24	1,96	2,35	0,0070	Putative integral membrane protein
SCO0779	1,11	1,20	1,27	1,32	2,15	0,1211	Uncharacterized protein
SCO0781	1,14	1,69	1,16	1,68	2,01	0,0740	Anti-sigma factor antagonist
SCO0832	1,72	1,40	1,05	2,45	2,97	0,0006	Uncharacterized protein
SCO0833	1,48	1,04	1,32	2,91	3,58	0,0007	Uncharacterized protein
SCO0834	1,18	1,60	0,93	2,08	1,84	0,0012	Uncharacterized protein
SCO0850	1,29	1,29	1,20	2,84	2,74	0,0012	Putative membrane protein
SCO0861	1,61	0,68	0,85	0,50	0,68	0,3720	Putative secreted protein
SCO0867	0,81	0,73	0,82	0,48	0,47	0,1618	Putative integral-membrane protein
SCO0876	0,69	1,03	0,39	1,14	1,04	0,4689	Uncharacterized protein
SCO0919	1,16	1,15	0,94	2,07	2,21	0,0848	Uncharacterized protein
SCO0930	0,52	1,02	0,54	0,47	0,55	0,0106	Putative lipoprotein
SCO0931	0,76	0,86	0,50	1,00	1,05	0,4654	Putative secreted proline-rich protein
SCO0933	0,88	0,91	1,45	2,16	2,13	0,0638	Putative lipoprotein
SCO0934	2,16	7,64	1,53	9,20	11,38	0,0000	Putative integral membrane protein
SCO0935	1,15	1,34	1,42	1,91	2,62	0,0174	Putative secreted protein
SCO0936	1,44	2,04	1,25	1,85	2,44	0,0042	Putative oligosaccharide deacetylase
SCO0939	1,35	0,93	0,39	1,04	0,88	0,6534	Putative hydrolase
SCO0941	1,56	1,56	1,06	2,22	2,25	0,0046	Putative lipoprotein

SCO0955	0,47	0,58	0,51	0,45	0,51	0,0087	Uncharacterized protein
SCO0985	0,91	0,71	0,90	0,53	0,44	0,2399	MetE
SCO0986	0,59	0,80	0,63	0,62	0,49	0,0726	Putative integral membrane protein
SCO0989	0,76	0,50	1,07	1,14	1,09	0,5637	Uncharacterized protein
SCO0995	0,60	0,58	0,68	0,48	0,56	0,0273	Putative methyltransferase
SCO0996	0,53	0,59	0,52	0,46	0,43	0,0057	Putative lipoprotein
SCO0999	0,73	0,58	1,16	0,34	0,50	0,0246	Superoxide dismutase (EC 1.15.1.1)
SCO1023	0,42	0,76	0,92	0,61	0,74	0,2287	Putative membrane protein
SCO1053	2,07	0,79	1,45	1,23	1,11	0,2838	Uncharacterized protein
SCO1088	1,44	2,12	1,19	1,94	1,90	0,0050	Putative oxidoreductase
SCO1089	1,60	2,11	0,89	2,18	2,59	0,0046	Uncharacterized protein
SCO1105	0,49	0,99	0,44	0,78	0,60	0,0826	Putative secreted protein
SCO1107	2,11	1,56	1,32	2,41	1,99	0,0012	Uncharacterized protein
SCO1108	0,91	1,15	0,82	2,02	0,94	0,5799	Putative regulatory protein
SCO1143	1,94	2,37	1,51	3,41	3,53	0,0000	Uncharacterized protein
SCO1150	1,83	1,15	1,86	1,48	2,54	0,0103	RpmE2, 50S ribosomal protein L31 type B 1
SCO1158	0,90	0,58	1,30	0,48	0,59	0,2146	Uncharacterized protein
SCO1161	0,45	0,59	0,83	0,59	0,53	0,0742	Putative integral membrane protein
SCO1172	1,69	2,18	0,89	1,78	1,75	0,0054	Putative amidase (Putative secreted protein)
SCO1178	2,33	1,74	1,14	1,05	1,07	0,0519	Uncharacterized protein
SCO1195	1,33	1,82	1,33	2,27	2,69	0,0008	Putative membrane protein
SCO1197	0,49	0,65	1,38	0,68	0,56	0,2791	Putative acyl-CoA dehydrogenase
SCO1200	0,52	0,46	0,84	0,51	0,40	0,0225	Putative regulatory protein
SCO1205	0,52	0,43	0,80	2,28	1,76	0,3145	Uncharacterized protein
SCO1208	1,32	1,24	1,37	1,65	2,01	0,0652	Uncharacterized protein
SCO1216	0,39	0,77	1,48	0,86	0,83	0,6515	Putative integral membrane protein
SCO1235	1,60	2,17	0,47	0,91	1,01	0,1835	UreB, Urease subunit beta
SCO1236	1,65	1,95	0,43	0,92	0,84	0,4940	UreA, Urease subunit gamma
SCO1288	2,03	1,55	0,79	1,15	1,13	0,3279	Putative integral membrane protein
SCO1319	1,31	2,27	1,06	1,80	1,83	0,0061	Putative membrane protein
SCO1346	1,11	2,15	1,15	1,56	1,34	0,0219	Putative 3-oxoacyl-[acyl-carrier protein] reductase
SCO1357	0,42	0,76	0,69	0,52	0,68	0,0057	Uncharacterized protein
SCO1403	0,42	0,72	1,26	0,92	0,82	0,5162	Putative membrane protein
SCO1415	1,13	2,58	1,03	3,44	3,47	0,0000	Putative membrane protein
SCO1416	1,08	2,17	1,42	2,89	3,42	0,0003	Putative membrane protein
SCO1426	1,07	1,43	1,03	3,35	3,40	0,0009	Uncharacterized protein
SCO1431	0,85	1,30	1,09	2,14	2,03	0,0407	Putative membrane protein
SCO1451	0,67	0,69	0,79	0,50	0,48	0,0997	Glucanase (EC 3.2.1.-)
SCO1480	0,64	1,02	0,56	0,47	0,60	0,0981	Uncharacterized protein
SCO1485	0,83	0,42	0,83	0,94	0,77	0,3451	Putative integral membrane protein
SCO1488	0,66	0,46	0,64	1,23	0,61	0,1483	PyrR, Bifunctional protein PyrR
SCO1489	0,45	0,66	0,81	0,67	0,89	0,1745	Putative DNA-binding protein
SCO1540	0,45	0,82	0,89	0,93	1,13	0,5805	Putative membrane protein
SCO1541	1,42	3,46	1,15	4,52	6,78	0,0000	SsgB, Sporulation-specific cell division protein SsgB
SCO1550	0,35	0,32	0,90	0,45	0,67	0,0008	Putative small membrane protein
SCO1557	0,31	0,57	0,86	0,31	0,32	0,0068	Lipoprotein
SCO1558	0,57	0,94	0,89	0,37	0,49	0,0944	Putative ABC transporter permease protein
SCO1571	0,55	0,82	0,48	0,51	0,58	0,0383	Uncharacterized protein
SCO1575	0,47	0,79	0,61	1,28	1,29	0,2031	Putative thiamine biosynthesis lipoprotein
SCO1599	0,77	1,08	0,50	0,38	0,59	0,0392	RpmI, 50S ribosomal protein L35
SCO1627	0,66	0,68	0,68	0,38	0,43	0,0180	Putative ATP-GTP binding protein
SCO1628	0,64	0,64	0,67	0,55	0,47	0,0490	Uncharacterized protein
SCO1629	0,57	0,81	0,68	0,47	0,50	0,0546	Uncharacterized protein
SCO1630	0,63	0,69	0,74	0,45	0,50	0,0523	Putative integral membrane protein
SCO1643	0,39	0,74	0,98	0,74	0,90	0,4048	PrcA, 20S proteasome alpha subunit PrcA
SCO1644	0,45	0,80	0,98	0,76	1,09	0,6738	PrcB, 20S proteasome beta subunit PrcB
SCO1645	0,44	0,78	0,73	0,66	0,86	0,1874	Uncharacterized protein
SCO1659	0,58	1,54	1,09	2,95	2,11	0,0288	GlpF, Probable glycerol uptake facilitator protein
SCO1660	0,48	1,54	0,97	2,09	1,68	0,8755	GlpK2, Glycerol kinase 2
SCO1661	0,33	0,97	1,27	1,81	1,41	0,9285	Putative glycerol-3-phosphate dehydrogenase
SCO1662	0,45	0,83	1,06	1,18	1,01	1,0299	Uncharacterized protein
SCO1668	1,62	1,13	0,91	2,19	2,44	0,0140	Uncharacterized protein

SCO1675	0,73	0,88	0,82	0,41	0,86	0,1245	Putative small membrane protein
SCO1700	1,22	2,91	0,79	0,73	1,51	0,0159	Putative membrane protein
SCO1708	0,36	0,72	1,07	1,07	1,00	0,4819	Putative membrane protein
SCO1749	1,09	2,02	1,07	2,04	2,85	0,0021	SepF1, Cell division protein SepF 1
SCO1781	0,49	0,85	0,83	0,79	0,85	0,2976	NadK2, NAD kinase 2
SCO1792	2,01	1,79	1,03	0,62	0,91	0,3302	Putative 3-methyladenine DNA glycosylase
SCO1800	0,44	0,61	0,87	0,26	0,48	0,0054	Putative small secreted protein
SCO1811	0,52	0,75	0,72	0,52	0,37	0,0161	Putative membrane protein
SCO1828	0,91	2,83	1,21	2,33	2,62	0,0000	Uncharacterized protein
SCO1860	0,76	1,07	0,84	0,66	0,43	0,2149	Putative secreted protein
SCO1862	0,45	0,98	1,26	0,93	0,97	1,1326	Putative integral membrane protein
SCO1874	0,67	1,35	1,04	2,76	2,14	0,0299	Uncharacterized protein
SCO1913	0,46	0,85	1,11	1,01	0,93	0,9808	Putative small membrane protein
SCO1919	0,51	0,88	0,73	0,36	0,46	0,0250	Uncharacterized protein
SCO1920	0,60	0,93	0,81	0,42	0,47	0,1066	Uncharacterized protein
SCO1921	0,75	1,14	0,59	0,50	0,53	0,1849	Csd, Probable cysteine desulfurase
SCO1922	0,69	1,09	0,60	0,39	0,40	0,0415	Putative ABC transporter ATP-binding subunit
SCO1923	0,70	0,94	0,50	0,32	0,35	0,0087	Putative dioxygenase ferredoxin subunit
SCO1925	0,52	0,80	0,69	0,50	0,52	0,0737	Uncharacterized protein
SCO1926	0,49	0,59	0,76	0,45	0,48	0,0243	Putative DNA-binding protein
SCO1934	0,43	0,80	0,79	0,82	0,84	0,4238	CtaB, Protoheme IX farnesyltransferase
SCO1943	0,52	0,76	0,54	0,53	0,47	0,0213	RbpA, RNA polymerase-binding protein RbpA
SCO1955	0,44	1,32	1,45	2,00	1,14	1,2242	Putative iron sulphur binding protein
SCO1978	0,53	1,31	1,23	4,36	1,79	0,0033	Putative AbaA-like protein
SCO1980	0,43	0,61	0,98	0,65	0,62	0,1317	Uncharacterized protein
SCO1982	0,64	1,15	0,97	1,03	2,01	0,7197	Uncharacterized protein
SCO2008	0,55	0,83	0,43	0,37	0,37	0,0045	Putative branched chain amino acid binding protein
SCO2009	0,63	0,91	0,48	0,67	0,64	0,1000	Putative branched chain amino acid transport permease
SCO2025	1,34	1,51	0,87	1,73	2,19	0,0496	Putative glutamate synthase small subunit
SCO2026	1,17	1,43	0,73	1,63	2,19	0,0813	Putative glutamate synthase large subunit
SCO2041	0,51	0,64	0,85	2,01	0,84	1,1979	Putative membrane protein
SCO2078	0,38	0,58	0,52	0,34	0,45	0,0008	Putative membrane protein
SCO2097	0,45	0,72	0,87	0,77	0,76	0,2366	Putative membrane protein
SCO2113	1,63	3,31	0,76	1,50	1,41	0,0070	Bfr, Bacterioferritin (BFR) (EC 1.16.3.1)
SCO2141	0,45	1,15	1,00	1,44	0,77	0,9741	Putative small secreted hydrophilic protein
SCO2153	0,83	1,11	1,21	1,80	2,11	0,0995	Putative secreted protein
SCO2161	0,48	0,62	0,60	0,35	0,42	0,0083	Uncharacterized protein
SCO2167	0,45	0,82	0,96	0,90	0,74	0,6207	Uncharacterized protein
SCO2172	0,50	0,74	0,74	0,58	0,47	0,0322	Uncharacterized protein
SCO2195	0,78	1,20	0,42	0,58	0,60	0,1804	Uncharacterized protein
SCO2199	0,45	0,98	0,72	1,47	1,71	0,8615	Putative integral membrane protein
SCO2207	0,37	0,73	0,57	0,43	0,46	0,0069	Hypothetical secreted protein
SCO2209	0,48	0,84	0,81	0,94	1,08	0,4695	Putative transcriptional regulator
SCO2210	2,82	3,55	0,43	1,93	1,50	0,0005	Glutamine synthetase (EC 6.3.1.2)
SCO2211	2,51	2,88	0,83	1,68	1,38	0,0009	Uncharacterized protein
SCO2212	0,36	0,54	0,41	0,28	0,22	0,0000	Uncharacterized protein
SCO2213	0,45	0,52	0,76	0,62	0,41	0,0193	Putative regulatory protein
SCO2217	0,52	0,87	0,42	0,28	0,32	0,0000	Putative secreted protein
SCO2247	0,39	0,77	1,03	0,87	0,80	0,5545	Uncharacterized protein
SCO2260	0,40	0,78	0,76	1,20	1,19	0,5184	Putative membrane protein
SCO2261	0,48	0,79	0,63	3,61	2,17	0,0119	Putative membrane protein
SCO2288	1,33	1,94	1,28	1,82	2,01	0,0054	Uncharacterized protein
SCO2314	0,47	0,91	1,04	0,93	1,10	1,0254	Putative integral membrane protein
SCO2354	0,43	0,83	0,86	1,20	1,36	0,4699	Uncharacterized protein
SCO2357	0,48	0,71	0,86	0,54	0,60	0,1167	Uncharacterized protein
SCO2367	0,49	0,86	0,70	0,58	0,62	0,1191	Uncharacterized protein
SCO2368	0,48	0,83	0,78	0,62	0,60	0,1381	Uncharacterized protein
SCO2389	0,49	0,71	0,58	0,47	0,83	0,0665	AcpP, Acyl carrier protein (ACP)
SCO2447	0,50	0,67	1,18	0,86	0,88	0,7015	Uncharacterized protein
SCO2451	1,48	1,78	1,36	2,29	2,70	0,0009	Putative rod shape-determining protein
SCO2478	3,11	1,95	1,29	1,00	1,22	0,0162	Putative reductase
SCO2486	2,00	2,48	0,66	1,71	1,32	0,0013	Putative nitrite reductase NirB

SCO2487	2,27	3,64	0,47	1,67	1,07	0,0007	Putative nitrite reductase large subunit NirB
SCO2488	1,63	3,25	0,67	2,45	2,18	0,0003	Putative nitrite reductase small subunit NirC
SCO2489	1,36	2,31	0,63	1,08	0,76	0,0376	Putative tetR-family transcriptional regulator
SCO2504	0,56	0,74	0,74	0,45	0,43	0,0341	GlyQS, Glycine-tRNA ligase GlyRS
SCO2505	0,14	0,12	0,76	0,13	0,13	0,0000	Putative ABC-transporter metal-binding lipoprotein
SCO2506	0,22	0,30	0,78	0,24	0,25	0,0003	Putative metal transport ABC transporter
SCO2507	0,32	0,33	0,92	0,27	0,29	0,0029	Putative metal transport ABC transporter
SCO2512	0,62	0,53	1,07	0,41	0,53	0,0767	Uncharacterized protein
SCO2513	0,40	0,51	0,93	0,44	0,34	0,0176	Uncharacterized protein
SCO2514	0,51	0,50	0,80	0,31	0,22	0,0037	Uncharacterized protein
SCO2550	0,46	0,89	0,94	0,96	1,03	0,7674	Putative lipoprotein
SCO2554	0,50	0,78	0,87	0,76	0,67	0,3948	DnaJ2, Chaperone protein DnaJ 2
SCO2555	0,47	0,67	0,72	0,77	0,70	0,1445	HrcA, Heat-inducible transcription repressor HrcA
SCO2573	0,44	0,79	0,68	0,53	0,53	0,0245	Putative oxidoreductase
SCO2574	0,40	0,71	0,41	0,44	0,41	0,0017	Uncharacterized protein
SCO2580	0,31	0,44	1,00	0,44	0,46	0,0192	Putative membrane protein
SCO2581	0,43	0,92	1,14	0,78	0,74	0,6480	Putative membrane protein
SCO2591	0,43	0,71	0,84	0,58	0,65	0,0523	Putative secreted protein
SCO2592	0,62	0,76	0,64	0,51	0,49	0,0443	Exopolysaccharide phosphotransferas
SCO2597	0,59	0,85	0,56	0,36	0,46	0,0095	RplU, 50S ribosomal protein L21
SCO2629	0,55	0,70	3,47	1,44	1,13	0,3701	Putative membrane protein
SCO2633	0,55	0,44	0,99	0,26	0,40	0,0051	Sod2, Superoxide dismutase
SCO2634	0,58	0,67	1,15	0,35	0,39	0,0327	Uncharacterized protein
SCO2636	1,21	1,64	1,22	1,90	2,91	0,0046	Uncharacterized protein
SCO2637	1,03	1,42	0,98	1,57	2,16	0,1900	Putative serine protease
SCO2641	0,45	0,66	0,92	0,62	0,58	0,1024	Putative resistance protein
SCO2648	0,36	0,71	0,81	1,30	1,08	0,6975	Putative membrane protein
SCO2683	1,16	1,09	1,60	1,64	2,01	0,0692	Ssb1, Single-stranded DNA-binding protein 1 (SSB 1)
SCO2684	1,02	1,60	1,30	2,37	2,22	0,0102	Putative ATP-binding membrane protein
SCO2699	0,89	2,05	1,02	1,71	2,38	0,0048	Putative small membrane protein
SCO2704	1,10	2,37	1,33	3,16	3,51	0,0000	Putative secreted protein
SCO2705	1,87	2,37	1,31	3,53	4,54	0,0000	Putative membrane protein
SCO2716	3,15	3,69	1,29	4,79	7,68	0,0000	Putative secreted protein
SCO2718	3,62	2,61	0,91	2,25	6,21	0,0000	Putative secreted protein
SCO2719	2,57	2,12	0,79	1,63	3,74	0,0002	Putative secreted protein
SCO2725	0,48	0,78	1,09	1,11	0,86	0,9094	Putative lipoprotein SCO2725
SCO2728	1,12	1,75	1,17	1,48	2,10	0,0582	Uncharacterized protein
SCO2729	0,73	1,31	0,90	2,03	2,33	0,1468	Putative acetyltransferase
SCO2730	0,48	0,97	1,23	1,27	1,47	1,0258	Putative regulator
SCO2768	0,46	0,75	0,88	0,69	0,75	0,2787	Uncharacterized protein
SCO2774	1,59	2,57	0,89	1,71	1,56	0,0018	Acyl-CoA dehydrogenase
SCO2779	1,34	0,93	1,24	2,07	1,76	0,0379	Acyl-CoA dehydrogenase
SCO2795	1,79	2,40	0,89	1,75	2,27	0,0073	Putative sugar binding secreted protein
SCO2797	1,30	1,61	1,13	1,56	2,06	0,0641	Putative cellobiose transport permease
SCO2819	0,38	0,42	0,87	0,29	0,35	0,0054	Uncharacterized protein
SCO2830	0,57	0,91	1,04	2,37	1,52	0,4533	Probable amino acid ABC transporter protein
SCO2838	0,45	0,75	0,90	0,71	0,69	0,2338	Glucanase (EC 3.2.1.-)
SCO2845	2,41	1,18	0,82	0,98	1,15	0,4459	Putative GntR-family transcriptional regulatory protein
SCO2858	0,50	0,78	1,11	1,15	0,94	1,1319	Uncharacterized protein
SCO2882	0,77	0,81	0,68	0,49	0,53	0,0721	Putative conserved ATP/GTP-binding protein
SCO2887	0,43	0,96	1,20	0,96	0,96	1,1095	Putative membrane protein
SCO2905	0,26	0,46	0,89	0,71	0,54	0,0086	Uncharacterized protein
SCO2910	0,35	0,50	0,80	0,41	0,42	0,0066	Putative cysteine synthase
SCO2911	0,43	0,35	0,82	0,29	0,31	0,0044	Uncharacterized protein
SCO2912	0,42	0,44	0,59	0,32	0,32	0,0014	Uncharacterized protein
SCO2924	1,86	3,66	1,11	3,92	4,85	0,0000	Putative regulator
SCO2928	2,05	1,30	1,13	1,80	1,87	0,0251	Putative asnC-family transcriptional regulator
SCO2950	1,07	2,13	0,67	0,81	0,99	0,1829	Hup1, DNA-binding protein HU 1 (HSl)
SCO2953	0,51	0,73	0,59	0,48	0,64	0,0182	Putative membrane protein
SCO2954	0,59	0,84	0,57	0,47	0,52	0,0109	RNA polymerase sigma factor
SCO2959	1,85	2,91	0,39	1,13	1,09	0,0073	Putative nitrate extrusion protein
SCO2970	0,33	0,81	0,97	0,74	0,59	0,1501	Putative membrane protein

SCO2971	0,45	0,77	0,72	0,35	0,45	0,0109	Putative secreted protein
SCO2976	0,38	0,66	0,58	0,58	0,62	0,0159	Uncharacterized protein
SCO3013	0,33	0,68	0,87	0,52	0,64	0,0503	Putative two-component system response regulator
SCO3023	0,51	0,76	0,76	0,32	0,40	0,0180	AhcY, Adenosylhomocysteinase
SCO3035	0,69	0,60	1,14	0,47	0,46	0,1226	Uncharacterized protein
SCO3047	1,21	1,96	1,07	1,98	2,34	0,0095	Putative membrane protein
SCO3048	0,87	2,06	0,87	1,27	1,72	0,0383	Putative membrane protein
SCO3051	1,44	2,29	0,86	1,36	1,51	0,0110	Acyl-CoA dehydrogenase
SCO3063	0,40	0,81	0,83	0,84	0,79	0,3220	Putative two-component system reponse regulator
SCO3067	0,41	0,70	0,91	1,02	1,26	0,4227	ArsI, Anti-sigma factor antagonist
SCO3069	0,48	0,94	0,87	1,46	1,17	0,9121	Putative integral membrane protein
SCO3086	1,18	3,00	1,02	1,14	1,82	0,0045	Putative lipoprotein
SCO3087	0,47	1,01	0,99	1,20	1,13	1,0871	Uncharacterized protein
SCO3088	1,10	2,21	0,72	0,89	1,00	0,1444	Uncharacterized protein
SCO3098	0,60	0,64	0,95	0,49	0,51	0,1253	Putative secreted protein
SCO3105	0,87	0,93	0,44	0,88	0,37	0,0292	Uncharacterized protein
SCO3106	0,91	0,91	0,47	0,91	0,63	0,0871	Putative lipoprotein
SCO3108	0,72	2,24	1,12	2,27	1,56	0,0269	Uncharacterized protein
SCO3112	0,57	0,87	0,99	0,57	0,49	0,2392	SCO3112 protein (Fragment)
SCO3121	0,21	0,36	0,95	0,57	0,51	0,0069	Uncharacterized protein
SCO3124	0,40	0,76	0,79	0,51	0,57	0,0609	RplY, 50S ribosomal protein L25
SCO3152	1,71	1,42	1,42	1,84	2,67	0,0047	Uncharacterized protein
SCO3167	1,92	1,14	0,57	2,35	2,41	0,0068	Putative tetR-family transcriptional regulator
SCO3169	0,66	1,30	1,39	2,16	1,71	0,0223	Uncharacterized protein
SCO3182	0,62	1,05	0,64	0,49	0,48	0,0989	UTP--glucose-1-phosphate uridylyltransferase
SCO3187	0,32	0,46	0,59	0,51	0,49	0,0027	Uncharacterized protein
SCO3206	0,45	0,30	1,13	0,20	0,16	0,0015	Putative transmembrane efflux protein
SCO3207	0,38	0,27	0,96	0,21	0,17	0,0003	Putative tetR-family transcriptional regulator (Fragment)
SCO3218	0,24	1,54	0,77	0,72	0,69	0,1571	Uncharacterized protein
SCO3254	0,96	1,89	0,35	1,09	0,99	0,5121	SpdC protein
SCO3255	0,42	0,80	1,08	1,14	1,39	0,4499	SpdB protein
SCO3266	0,73	0,82	1,50	2,24	2,10	0,0522	Uncharacterized protein
SCO3270	0,45	0,53	1,39	1,52	2,07	0,4737	Uncharacterized protein
SCO3278	0,63	2,02	1,45	2,89	3,54	0,0003	Uncharacterized protein
SCO3290	0,35	0,59	0,55	0,48	0,70	0,0137	Uncharacterized protein
SCO3309	0,92	1,17	2,04	1,17	1,58	0,0638	Uncharacterized protein
SCO3323	0,54	0,74	0,60	0,36	0,47	0,0128	Putative RNA polymerase sigma factor
SCO3327	0,48	1,65	0,60	1,12	0,95	0,5353	Uncharacterized protein
SCO3328	0,40	0,92	1,14	0,72	1,07	0,4430	Uncharacterized protein
SCO3342	0,78	2,23	1,02	1,38	1,76	0,0312	Putative glycine-rich secreted protein
SCO3352	0,44	0,68	0,95	0,90	0,88	0,4148	DisA, DNA integrity scanning protein DisA (Cyclic di-AMP synthase)
SCO3379	1,12	2,02	0,85	2,03	1,95	0,0125	Uncharacterized protein
SCO3386	0,39	0,85	1,34	0,93	1,10	1,0248	Uncharacterized protein
SCO3409	0,42	0,65	0,95	0,48	0,52	0,0560	Ppa, Inorganic pyrophosphatase
SCO3423	0,48	0,65	0,69	0,66	0,59	0,0623	Putative regulator
SCO3424	0,40	0,54	0,73	0,39	0,51	0,0077	Putative regulator
SCO3425	0,16	0,28	0,85	0,30	0,24	0,0004	RpsR2, 30S ribosomal protein S18 2
SCO3426	0,17	0,19	0,92	0,18	0,17	0,0000	Uncharacterized protein
SCO3427	0,09	0,09	1,27	0,06	0,06	0,0000	RpmE2, 50S ribosomal protein L31 type B 2
SCO3428	0,10	0,12	0,65	0,09	0,08	0,0000	RpmG1 , 50S ribosomal protein L33 1
SCO3429	0,20	0,23	1,15	0,20	0,24	0,0008	RpmB2, 50S ribosomal protein L28-2
SCO3430	0,26	0,15	1,46	0,13	0,17	0,0000	RpsN, 30S ribosomal protein S14
SCO3431	0,25	0,28	1,11	0,22	0,24	0,0028	Possible membrane protein
SCO3437	0,67	0,55	1,22	0,42	0,34	0,0744	Putative integral membrane protein
SCO3438	0,93	0,43	1,24	0,24	0,17	0,0160	Uncharacterized protein
SCO3442	0,90	0,77	0,97	0,53	0,40	0,3127	Uncharacterized protein
SCO3444	0,83	0,84	0,99	0,57	0,46	0,4636	Uncharacterized protein
SCO3445	0,46	0,75	0,79	0,25	0,20	0,0036	Putative small membrane protein
SCO3447	0,70	0,79	0,77	0,33	0,25	0,0192	Putative transcriptional regulatory protein
SCO3448	0,93	0,82	0,73	0,38	0,28	0,0575	Uncharacterized protein
SCO3449	0,87	0,92	0,97	0,40	0,31	0,0849	Uncharacterized protein
SCO3450	0,61	0,71	0,79	0,29	0,26	0,0137	RNA polymerase sigma factor

SCO3452	0,85	1,15	0,67	0,55	0,47	0,2497	Putative methyltransferase
SCO3455	0,94	0,85	0,81	0,49	0,47	0,3086	Putative ABC-transporter transport protein
SCO3456	0,69	0,76	0,92	0,46	0,37	0,1223	Putative secreted protein
SCO3460	1,04	0,94	0,96	0,52	0,44	0,4867	Putative oxidoreductase
SCO3461	0,69	0,76	0,87	0,74	0,37	0,1118	Putative integral membrane protein
SCO3462	0,86	0,98	0,74	0,52	0,42	0,2740	Uncharacterized protein
SCO3463	1,08	1,13	0,77	0,45	0,36	0,2365	Putative phosphorylase
SCO3465	1,03	0,99	0,63	0,50	0,41	0,2401	Uncharacterized protein
SCO3471	0,85	1,50	1,69	0,61	1,11	0,0038	DagA, Extracellular agarase (EC 3.2.1.81)
SCO3472	1,39	1,83	3,29	1,30	3,82	0,0000	Putative transposase remnant
SCO3473	0,36	0,08	0,20	0,08	0,12	0,0000	Putative aldolase
SCO3474	0,18	0,03	0,07	0,02	0,06	0,0000	Putative sugar kinase
SCO3475	0,24	0,05	0,08	0,04	0,05	0,0000	Putative isomerase
SCO3476	0,26	0,11	0,16	0,08	0,12	0,0000	Putative short-chain dehydrogenase
SCO3477	0,42	0,22	0,34	0,18	0,22	0,0000	Putative dehydrogenase
SCO3478	0,56	0,88	1,00	0,44	1,12	0,3147	Putative dehydrogenase
SCO3481	0,69	1,17	0,80	0,42	1,13	0,3472	Uncharacterized protein
SCO3482	0,55	1,01	0,66	0,40	0,98	0,0839	Putative sugar-permease
SCO3483	0,50	1,07	0,89	0,45	1,15	0,3067	Putative integral membrane transport protein
SCO3484	0,47	1,17	0,81	0,41	1,11	0,1508	Putative secreted sugar-binding protein
SCO3486	0,44	0,75	0,93	0,45	0,72	0,1129	Putative aldehyde dehydrogenase
SCO3487	0,63	1,29	0,90	0,48	1,17	0,3857	Putative hydrolase
SCO3529	1,46	0,89	2,22	1,22	1,39	0,1563	Uncharacterized protein
SCO3537	0,62	0,61	0,50	0,31	0,37	0,0026	Putative DNA-binding protein
SCO3538	0,55	0,60	0,42	0,31	0,37	0,0006	Uncharacterized protein
SCO3548	0,30	0,95	1,26	0,87	0,76	0,4561	Putative anti-sigma factor
SCO3549	0,57	1,12	0,73	0,48	0,73	0,2184	BldG, Anti-sigma-B factor antagonist
SCO3561	1,26	1,96	1,42	3,05	3,62	0,0000	Putative secreted protein
SCO3571	0,38	0,67	0,60	0,57	0,59	0,0189	Putative transcriptional regulator
SCO3575	0,47	1,01	0,88	0,78	0,87	0,7232	Uncharacterized protein
SCO3576	0,32	0,61	0,70	0,66	0,67	0,0304	Uncharacterized protein
SCO3579	0,41	0,73	1,05	0,53	0,87	0,1640	WblA, Transcriptional regulator WhiB
SCO3584	0,44	0,53	1,15	0,88	0,97	0,3722	Uncharacterized protein
SCO3591	0,35	0,80	1,05	1,35	1,17	0,5673	Putative small membrane protein
SCO3600	2,07	1,08	0,81	0,97	0,87	1,0349	Uncharacterized protein
SCO3604	0,50	1,00	1,05	1,01	0,92	1,1982	Putative membrane protein
SCO3607	0,99	1,69	0,94	1,45	2,23	0,1741	Putative secreted protein
SCO3608	1,01	1,09	1,28	1,51	2,05	0,2650	Uncharacterized protein
SCO3657	0,79	0,98	1,29	1,95	2,84	0,0451	Uncharacterized protein
SCO3662	0,49	0,43	0,54	0,42	0,42	0,0044	Uncharacterized protein
SCO3663	0,21	0,34	1,01	0,82	1,48	0,0143	Putative membrane protein
SCO3664	0,47	0,68	0,65	0,59	0,51	0,0304	Putative regulatory protein
SCO3668	0,52	0,73	0,41	0,75	0,73	0,0609	HspR, Putative heat shock protein HspR
SCO3671	0,64	0,80	0,36	0,90	1,08	0,1240	DnaK, Chaperone protein DnaK (HSP70)
SCO3701	0,85	0,86	2,86	0,68	0,89	0,3915	Uncharacterized protein
SCO3712	1,15	2,13	0,91	1,69	2,06	0,0050	Putative hydrolase
SCO3713	0,42	0,82	0,84	0,73	0,71	0,3350	Uncharacterized protein
SCO3714	0,53	0,83	0,71	0,41	0,46	0,0424	Putative transposase
SCO3731	0,74	1,43	0,82	0,49	0,76	0,3719	Cold-shock protein
SCO3732	1,64	2,43	0,91	1,04	1,20	0,0500	Putative DEAD-box RNA helicase
SCO3748	0,67	1,20	0,59	0,49	0,87	0,1230	Cold shock protein
SCO3762	0,50	0,40	0,63	1,35	2,92	0,4628	Uncharacterized protein
SCO3793	0,43	1,17	0,51	0,69	0,67	0,1327	Uncharacterized protein
SCO3800	1,48	2,22	0,87	1,98	2,08	0,0012	Putative acyl-CoA dehydrogenase
SCO3802	0,40	0,98	0,84	1,45	1,40	0,5939	Putative membrane protein
SCO3805	1,44	0,99	1,19	1,82	2,06	0,0999	Uncharacterized protein
SCO3814	0,50	0,58	0,67	0,41	0,44	0,0083	Putative DNA-binding protein
SCO3852	0,54	0,54	0,59	0,48	0,53	0,0107	Putative membrane protein
SCO3858	0,57	0,68	0,70	0,46	0,47	0,0274	UppS2, Isoprenyl transferase 2 (EC 2.5.1.-)
SCO3862	1,24	2,80	0,84	2,18	2,45	0,0004	Putative membrane protein
SCO3863	2,18	7,83	1,03	6,09	6,53	0,0000	Putative secreted protein
SCO3867	1,35	1,77	1,00	2,73	3,42	0,0007	Putative ferredoxin

SCO3870	0,47	0,59	0,49	0,37	0,30	0,0010	Putative integral membrane protein
SCO3871	0,48	0,96	0,81	1,51	1,11	0,7331	Putative decarboxylase
SCO3880	0,50	0,79	0,82	0,39	0,48	0,0275	RpmH, 50S ribosomal protein L34
SCO3881	0,48	0,85	1,09	0,62	0,73	0,4606	rnpA, Ribonuclease P protein component (RNase P)
SCO3889	0,43	0,76	0,75	0,53	0,47	0,0489	TrxA, Thioredoxin-1 (Trx-1)
SCO3890	0,63	0,67	0,79	0,38	0,35	0,0249	TrxB, Thioredoxin reductase (TRXR) (EC 1.8.1.9)
SCO3899	0,43	0,86	0,66	0,69	0,91	0,0913	Uncharacterized protein
SCO3906	0,64	0,82	0,64	0,33	0,52	0,0189	RpsF, 30S ribosomal protein S6
SCO3907	0,77	1,11	0,77	0,57	0,74	0,6768	ssb2, Single-stranded DNA-binding protein 2 (SSB 2)
SCO3908	0,56	0,95	0,51	0,30	0,40	0,0065	RpsR1, 30S ribosomal protein S18 1
SCO3909	0,73	1,34	0,81	0,50	0,78	0,6799	RplI, 50S ribosomal protein L9
SCO3918	1,83	1,74	1,18	1,63	2,13	0,0068	Uncharacterized protein
SCO3924	0,48	0,52	0,60	0,65	0,67	0,0303	Uncharacterized protein SCO3924
SCO3937	1,09	0,82	0,77	0,74	0,61	0,4147	Putative integrase /recombinase
SCO3938	0,71	0,43	1,20	0,84	0,90	0,3616	Putative thiamin biosynthesis protein ThiC (Fragment)
SCO3962	0,49	0,67	0,72	0,49	0,50	0,0321	Prephenate dehydratase
SCO3965	0,94	0,73	0,57	0,37	0,36	0,0197	Uncharacterized protein
SCO3966	0,75	0,78	0,62	0,44	0,45	0,0536	Putative secreted protein
SCO3967	0,66	0,62	0,96	0,40	0,39	0,0415	Conserved hypothetical membrane protein
SCO3968	0,64	0,70	0,83	0,39	0,40	0,0292	Putative integral membrane protein
SCO3975	1,10	1,33	1,19	2,39	2,86	0,0048	Putative regulator
SCO3976	0,49	0,59	1,09	1,30	1,35	0,6152	Putative phosphodiesterase
SCO3986	0,83	1,75	1,24	2,09	1,51	0,0313	Putative GntR-family transcriptional regulator
SCO3990	0,42	0,53	0,88	0,85	0,66	0,0509	Uncharacterized protein
SCO3991	0,65	0,59	0,86	0,45	0,64	0,0665	Uncharacterized protein
SCO3992	1,09	0,48	0,87	0,53	0,50	0,0522	Uncharacterized protein
SCO3993	0,48	0,66	0,93	0,70	0,54	0,1402	Uncharacterized protein
SCO3998	0,60	0,68	0,95	0,49	0,58	0,1445	Uncharacterized protein
SCO4032	1,07	0,72	0,39	1,56	1,59	0,1364	Putative marR regulatory protein
SCO4037	0,50	0,50	0,91	0,66	0,76	0,1043	Putative small membrane protein
SCO4057	1,37	3,16	1,55	5,87	7,30	0,0000	Putative transport integral membrane protein
SCO4060	1,44	1,42	1,54	1,32	2,32	0,0153	Uncharacterized protein
SCO4088	0,47	0,85	0,70	0,66	0,51	0,0723	Uncharacterized protein
SCO4091	0,48	1,02	1,08	0,54	0,76	0,3394	Putative DNA-binding protein
SCO4100	0,82	0,73	0,77	0,57	0,48	0,1718	Uncharacterized protein
SCO4112	0,45	0,76	0,79	1,09	0,85	0,6912	Uncharacterized protein
SCO4113	0,47	0,78	1,08	1,07	0,99	0,9039	Uncharacterized protein
SCO4118	0,50	1,00	1,13	1,08	0,81	1,1321	Putative tetR-family transcriptional regulator
SCO4131	0,49	0,95	0,58	0,67	0,72	0,1044	Putative integral membrane protein
SCO4142	1,70	1,94	0,33	1,06	0,82	0,6800	Phosphate-binding protein PstS
SCO4164	0,48	0,48	0,62	0,36	0,46	0,0029	Sulfurtransferase
SCO4165	0,35	0,60	1,31	0,83	1,20	0,2744	Uncharacterized protein
SCO4178	0,46	0,79	0,47	0,52	0,55	0,0107	Putative small membrane protein
SCO4187	1,12	1,55	0,63	1,20	2,48	0,1106	Putative membrane protein
SCO4200	0,41	0,74	0,48	0,70	0,69	0,0252	Putative membrane protein
SCO4211	3,11	0,90	0,98	1,00	1,07	0,3839	Putative integrase
SCO4212	0,42	2,29	1,78	2,31	2,29	0,0009	Uncharacterized protein
SCO4214	0,32	0,26	0,76	0,22	0,17	0,0000	Putative AbaA-like regulatory protein
SCO4223	1,87	2,70	1,22	1,21	1,17	0,0160	Putative AraC-family transcriptional regulator
SCO4225	0,99	3,03	1,20	1,37	1,86	0,0050	Putative integral membrane protein
SCO4231	0,47	1,01	1,10	1,04	0,96	1,1755	Putative lipoprotein
SCO4232	0,31	0,79	0,58	0,50	0,56	0,0108	Putative transcriptional factor regulator
SCO4240	0,49	0,57	0,82	0,62	0,70	0,1214	ABC transporter ATP-binding protein
SCO4266	3,58	2,09	1,48	0,70	0,95	0,0079	EcaD1, Putative oxidoreductase
SCO4277	0,40	0,92	0,64	0,51	0,56	0,0372	Putative tellurium resistance protein
SCO4289	1,24	3,13	1,53	2,10	2,26	0,0003	Putative secreted protein
SCO4294	0,30	0,48	0,66	0,35	0,35	0,0006	Uncharacterized protein
SCO4295	0,40	0,69	0,44	0,40	0,54	0,0045	Cold shock protein
SCO4296	0,54	0,58	0,61	0,44	0,55	0,0103	GroEL2, 60 kDa chaperonin 2
SCO4297	0,74	0,73	0,96	0,45	0,39	0,1257	Putative oxidoreduxtase
SCO4300	0,28	0,72	1,06	0,82	0,89	0,1335	Uncharacterized protein
SCO4340	0,57	0,68	0,78	0,33	0,32	0,0156	Putative integrase

SCO4344	0,72	0,80	1,57	1,35	2,17	0,1901	Putative transposase
SCO4346	0,80	0,81	0,73	0,64	0,50	0,1255	Uncharacterized protein
SCO4347	0,70	0,99	0,52	0,75	0,49	0,1473	Uncharacterized protein
SCO4408	0,40	0,65	1,19	0,61	0,64	0,1567	Uncharacterized protein
SCO4409	0,51	0,71	0,77	0,52	0,49	0,0699	Putative RNA polymerase sigma factor
SCO4438	0,49	0,82	1,18	1,15	0,99	1,0205	Putative integral membrane protein
SCO4441	0,45	0,64	0,88	0,52	0,61	0,1041	Putative DNA-binding protein
SCO4442	0,51	0,48	0,78	0,24	0,55	0,0010	Uncharacterized protein
SCO4486	0,38	1,19	1,05	0,84	0,86	0,7324	Uncharacterized protein
SCO4496	0,71	0,79	0,96	0,49	0,46	0,2376	Putative acetyltransferase
SCO4497	0,61	0,73	0,52	0,53	0,39	0,0135	Putative membrane protein
SCO4498	0,39	0,48	0,87	0,48	0,41	0,0157	Putative proton transport protein
SCO4509	0,46	0,80	0,75	0,49	0,41	0,0244	Uncharacterized protein
SCO4520	2,20	1,13	1,32	1,70	1,23	0,0560	Uncharacterized protein
SCO4531	0,65	0,50	1,06	1,26	1,40	0,8160	Putative septum determining protein
SCO4540	0,84	0,99	0,71	0,65	0,45	0,1484	Uncharacterized protein
SCO4544	0,46	0,71	0,78	0,69	0,54	0,0973	Uncharacterized protein
SCO4557	0,42	0,68	1,13	1,06	1,64	0,4588	Uncharacterized protein
SCO4561	0,44	0,95	0,58	0,41	0,48	0,0106	Putative NLP/P60 family protein
SCO4581	0,41	0,61	0,76	0,50	0,55	0,0238	Uncharacterized protein
SCO4582	0,47	0,86	0,71	0,92	0,90	0,5343	Uncharacterized protein
SCO4596	0,48	0,61	0,93	1,02	0,93	0,2946	Putative response regulator
SCO4616	1,09	0,82	0,13	0,58	0,33	0,0047	Excisionase
SCO4619	0,98	1,00	0,42	0,83	0,57	0,3773	Putative integral membrane protein
SCO4623	1,36	0,50	0,91	0,90	0,77	0,2844	Uncharacterized protein
SCO4626	1,41	1,00	0,44	0,75	0,71	0,4101	Uncharacterized protein
SCO4633	0,82	0,86	1,07	2,10	1,17	0,3885	Uncharacterized protein
SCO4635	0,73	1,05	0,66	0,41	0,57	0,0720	RpmG2 , 50S ribosomal protein L33 2
SCO4636	0,82	0,88	0,65	0,47	0,65	0,1418	UPF0336 protein SCO4636
SCO4640	1,43	1,07	0,48	0,84	0,85	0,4320	Putative tetR-family transcriptional regulator
SCO4642	1,40	0,83	0,37	0,81	0,77	0,1191	Uncharacterized protein
SCO4646	0,32	0,56	0,86	0,36	0,45	0,0051	SecE, Protein translocase subunit SecE
SCO4648	0,75	1,06	0,57	0,38	0,55	0,0745	RplK, 50S ribosomal protein L11
SCO4649	0,65	0,98	0,55	0,41	0,63	0,0723	RplA, 50S ribosomal protein L1
SCO4652	0,65	0,92	0,81	0,48	0,97	0,4591	RplJ, 50S ribosomal protein L10
SCO4660	1,03	1,43	0,52	0,45	0,71	0,2897	RspG, 30S ribosomal protein S7
SCO4662	0,67	0,97	0,67	0,41	0,72	0,1416	Tuf1, Elongation factor Tu-1 (EF-Tu-1)
SCO4672	0,50	0,62	0,80	0,67	0,69	0,1357	Putative secreted protein
SCO4677	0,33	0,46	0,64	0,27	0,51	0,0014	Putative regulatory protein
SCO4678	0,49	0,45	0,71	0,33	0,42	0,0085	Uncharacterized protein
SCO4679	0,39	0,51	0,48	0,29	0,29	0,0000	Uncharacterized protein
SCO4683	0,48	0,59	1,03	0,64	0,68	0,2101	Glutamate dehydrogenase
SCO4684	0,47	1,23	0,78	0,52	0,53	0,0723	Cold shock protein
SCO4687	0,37	0,49	0,67	0,37	0,38	0,0016	Uncharacterized protein
SCO4701	0,47	0,66	0,57	0,31	0,48	0,0036	RpsJ, 30S ribosomal protein S10
SCO4702	0,82	0,98	0,58	0,36	0,64	0,0794	RplC, 50S ribosomal protein L3
SCO4704	0,82	1,21	0,85	0,46	0,79	0,5726	RplW, 50S ribosomal protein L23
SCO4706	0,57	0,68	0,44	0,35	0,50	0,0030	RpsS, 30S ribosomal protein S19
SCO4707	0,74	1,11	0,47	0,38	0,61	0,0534	RplV, 50S ribosomal protein L22
SCO4709	0,69	1,02	0,66	0,40	0,75	0,1627	RplP, 50S ribosomal protein L16
SCO4711	0,84	1,19	0,64	0,40	0,71	0,2302	RpsQ, 30S ribosomal protein S17
SCO4712	0,53	0,74	0,68	0,34	0,63	0,0212	RplN, 50S ribosomal protein L14
SCO4713	0,78	1,04	0,75	0,43	0,79	0,3282	RplX, 50S ribosomal protein L24
SCO4714	0,76	0,97	0,63	0,41	0,80	0,1974	RplE, 50S ribosomal protein L5
SCO4715	0,77	1,01	0,90	0,48	0,87	0,6632	RpsZ, 30S ribosomal protein S14 type Z
SCO4716	0,73	0,98	0,63	0,43	0,69	0,1663	RpsH, 30S ribosomal protein S8
SCO4717	0,74	0,91	0,76	0,48	0,85	0,4317	RplF, 50S ribosomal protein L6
SCO4718	0,73	1,02	0,60	0,42	0,75	0,1871	RplR, 50S ribosomal protein L18
SCO4720	0,64	0,96	0,75	0,47	0,80	0,2823	RpmD, 50S ribosomal protein L30
SCO4721	0,57	0,89	0,64	0,40	0,72	0,0842	RplO, 50S ribosomal protein L15
SCO4725	0,92	1,07	0,63	0,42	0,71	0,2135	InfA, Translation initiation factor IF-1
SCO4727	1,05	1,26	0,52	0,48	0,84	0,2907	RpsM, 30S ribosomal protein S13

SCO4730	0,89	1,36	0,55	0,48	0,88	0,4653	RplQ, 50S ribosomal protein L17
SCO4734	0,80	1,03	0,56	0,43	0,54	0,0645	RplM, 50S ribosomal protein L13
SCO4759	1,13	1,70	0,84	2,15	2,05	0,0159	Putative secreted protein
SCO4761	0,65	0,63	0,48	0,34	0,49	0,0028	GroES, 10 kDa chaperonin (GroES protein)
SCO4762	0,45	0,51	0,59	0,45	0,50	0,0045	GroEL1, 60 kDa chaperonin 1 (HSP58)
SCO4768	0,43	0,77	0,68	0,54	0,91	0,0995	Putative two-component regulator
SCO4779	1,01	2,09	0,87	1,11	1,45	0,1066	Serine/threonine protein kinase
SCO4789	0,48	0,73	0,43	0,49	0,52	0,0077	Putative integral membrane protein
SCO4793	0,37	0,49	0,91	0,30	0,36	0,0085	Putative NPL/P60 family secreted protein
SCO4822	0,46	0,91	0,63	1,04	0,98	0,6163	Putative integral membrane protein
SCO4858	0,62	0,89	0,44	0,58	0,72	0,0766	Putative succinate dehydrogenase membrane subunit
SCO4883	0,47	0,79	0,65	0,51	0,52	0,0417	Putative peptidase
SCO4895	0,47	0,73	0,89	0,95	0,73	0,4146	Putative ECF sigma factor
SCO4908	0,87	3,05	0,81	1,60	2,57	0,0018	Putative RNA polymerase sigma factor
SCO4920	0,47	0,88	0,83	0,54	0,72	0,2337	Putative deoR-family transcriptional regulator
SCO4956	0,54	0,52	0,66	0,36	0,33	0,0060	MsrA, Peptide methionine sulfoxide reductase MsrA
SCO4967	0,51	0,69	0,73	0,48	0,40	0,0310	Mca, Mycothiol S-conjugate amidase
SCO4983	0,36	0,76	0,87	0,64	0,66	0,1349	Uncharacterized protein
SCO5000	2,11	1,30	1,13	1,31	1,80	0,0403	Uncharacterized protein
SCO5007	0,37	0,56	0,97	0,91	0,97	0,1778	Putative septum site-determining protein
SCO5028	1,31	1,52	1,10	2,46	3,30	0,0017	Putative ATP-binding protein
SCO5029	0,67	1,00	0,56	0,31	0,47	0,0192	Putative secreted protein
SCO5032	1,70	2,27	0,51	1,07	1,02	0,1761	Alkyl hydroperoxide reductase
SCO5060	0,48	0,81	1,43	1,37	1,26	1,1995	Putative integral membrane protein
SCO5071	1,28	0,57	1,86	0,53	0,34	0,0895	Hydroxylacyl-CoA dehydrogenase
SCO5074	1,56	0,79	1,22	0,37	0,20	0,0542	Putative dehydratase
SCO5075	1,91	1,14	1,04	0,47	0,26	0,2395	Putative oxidoreductase
SCO5078	1,30	0,91	1,08	0,51	0,30	0,2959	Uncharacterized protein
SCO5079	1,37	0,79	1,25	0,63	0,37	0,5422	Uncharacterized protein
SCO5080	1,47	1,10	1,10	0,79	0,42	0,9473	Putative hydrolase
SCO5081	2,07	1,14	1,08	0,54	0,34	0,5452	Uncharacterized protein
SCO5083	3,83	1,92	1,32	0,99	1,26	0,0073	ActII-2, Probable actinorhodin transporter
SCO5084	5,06	1,93	1,35	0,97	1,50	0,0006	ActII-3, Putative membrane protein ActII-3
SCO5085	2,27	1,46	1,72	0,55	0,45	0,1122	ActII-4, Probable actinorhodin operon activatory protein
SCO5086	1,16	0,87	1,43	0,64	0,44	0,3573	ActIII, Putative ketoacyl reductase (EC 1.3.1.-)
SCO5088	0,88	0,67	0,97	0,74	0,36	0,1870	Actinorhodin PKS beta-ketoacyl synthase 2
SCO5089	2,12	0,73	1,61	0,30	0,14	0,0192	Actinorhodin PKSe acyl carrier protein
SCO5090	0,89	1,02	0,87	0,69	0,45	0,5340	Actinorhodin PKS bifunctional cyclase/dehydratase (ACTVII)
SCO5091	1,30	0,93	1,19	0,77	0,50	1,0974	Cyclase
SCO5092	1,52	1,11	0,98	0,70	0,45	0,8615	ActVB, Actinorhodin polyketide dimerase (EC 1.-.-.-) (actI ORF6)
SCO5117	0,97	1,59	1,00	1,98	2,14	0,0192	Putative secreted peptide-binding protein
SCO5123	0,29	0,72	0,44	0,63	1,07	0,0108	Putative small membrane protein
SCO5135	0,35	0,61	0,64	0,42	0,57	0,0085	Ferredoxin
SCO5137	0,48	0,59	0,94	0,63	0,65	0,1448	Putative ATP-binding protein
SCO5145	0,36	0,92	0,61	0,66	0,40	0,0157	Uncharacterized protein
SCO5169	0,36	0,67	1,13	1,07	0,57	0,2905	Putative ATP-binding protein
SCO5170	0,42	0,66	0,88	0,59	0,73	0,1492	Putative tetR-family transcriptional regulator
SCO5172	0,54	0,54	0,86	0,46	0,52	0,0497	Putative hydrolase
SCO5175	0,92	0,85	0,82	0,42	0,56	0,2426	Putative integral membrane protein
SCO5177	0,97	0,97	0,73	0,43	0,52	0,2194	Uncharacterized protein
SCO5178	0,47	0,63	0,74	0,32	0,37	0,0086	Putative sulfurylase
SCO5187	0,67	0,81	0,85	0,51	0,49	0,1035	Putative glutaredoxin-like protein
SCO5190	0,73	1,42	0,58	1,06	2,24	0,0453	WblC, Transcriptional regulator WhiB
SCO5191	0,78	1,81	0,66	0,98	2,58	0,0213	Uncharacterized protein
SCO5206	0,49	0,73	0,72	0,48	0,46	0,0244	Hydrogen peroxide sensitive repressor
SCO5216	0,65	0,83	0,70	0,50	0,55	0,1211	SigR, ECF RNA polymerase sigma factor SigR
SCO5217	0,54	0,56	0,74	0,45	0,51	0,0259	RsrA, Anti-sigma factor RsrA (Regulator of SigR)
SCO5221	0,43	0,75	0,75	0,75	0,66	0,1853	Def4, Peptide deformylase 4 (PDF 4)
SCO5227	0,44	0,78	1,81	0,72	1,33	0,5903	Putative redoxin
SCO5230	0,49	0,58	0,62	0,30	0,39	0,0027	Putative integral membrane protein
SCO5240	0,73	0,82	0,57	0,35	0,56	0,0133	WblE, Transcriptional regulator WhiB
SCO5245	0,54	0,72	0,49	0,55	0,46	0,0109	UshY, Putative uncharacterized protein ushY

SCO5249	1,45	1,62	0,76	1,77	2,21	0,0046	Putative nucleotide-binding protein
SCO5250	1,11	1,22	0,98	1,79	2,04	0,0469	Polyprenyl synthetase
SCO5257	0,85	0,75	0,97	0,44	0,34	0,1476	Methyltransferase
SCO5268	1,38	1,05	1,31	1,75	2,44	0,0606	Uncharacterized protein
SCO5269	0,90	1,16	0,98	2,02	2,28	0,0873	Uncharacterized protein
SCO5281	0,54	0,84	0,62	0,53	0,49	0,0611	Putative 2-oxoglutarate dehydrogenase
SCO5286	1,24	1,19	1,16	1,66	2,05	0,1351	Putative secreted hydrolase
SCO5303	1,21	1,59	1,47	2,50	3,41	0,0008	Putative membrane protein
SCO5314	1,08	2,36	1,32	2,98	4,75	0,0000	Uncharacterized protein SCO5314 (WhiE ORF VII)
SCO5315	1,73	3,46	0,91	4,10	6,48	0,0000	Putative polyketide cyclase (WhiE ORF VI)
SCO5318	1,09	1,77	1,21	2,24	2,46	0,0009	Putative polyketide beta-ketoacyl synthase 1 (WhiE ORF III)
SCO5319	1,21	2,57	1,11	2,43	2,86	0,0005	16.7 kDa protein in whiE locus (WhiE ORF II)
SCO5320	0,79	1,27	1,43	1,89	2,13	0,0521	42.8 kDa protein in whiE locus (WhiE ORF I)
SCO5321	1,07	1,56	1,33	2,09	2,33	0,0095	Putative polyketide hydroxylase (WhiE ORF VIII)
SCO5329	0,56	0,60	0,59	0,37	0,30	0,0035	Uncharacterized protein
SCO5351	0,35	0,52	0,90	0,49	0,61	0,0209	Putative regulatory protein
SCO5359	0,60	0,97	0,52	0,47	0,49	0,0251	RpmE, 50S ribosomal protein L31
SCO5374	0,50	0,98	0,76	0,66	0,78	0,4592	AtpC, F-ATPase epsilon subunit
SCO5389	0,64	1,52	0,45	0,55	0,70	0,2032	Uncharacterized protein
SCO5390	0,49	0,71	0,92	0,60	0,62	0,2026	Putative alkanal monooxygenase (Luciferase)
SCO5396	0,48	0,75	0,60	0,54	0,66	0,0731	Putative cellulose-binding protein
SCO5405	0,59	0,82	0,55	0,56	0,50	0,0417	Putative transcriptional regulator
SCO5409	0,41	0,73	0,78	1,06	0,99	0,4922	Putative membrane protein
SCO5439	0,98	2,10	0,77	1,27	1,10	0,0691	DNA helicase (EC 3.6.4.12)
SCO5461	1,07	2,18	0,73	1,78	1,70	0,0057	Putative secreted protein
SCO5464	0,55	0,59	0,70	0,48	0,39	0,0141	SCO5464 protein
SCO5484	1,96	1,84	0,37	3,03	3,55	0,0000	Small hydrophobic membrane protein
SCO5485	1,18	1,01	0,68	2,21	1,95	0,0138	Small hydrophobic membrane protein
SCO5514	0,57	0,96	0,65	0,50	0,65	0,1679	IlvC1, Ketol-acid reductoisomerase 1
SCO5521	0,91	0,79	0,48	0,68	0,85	0,2156	Uncharacterized protein
SCO5537	0,53	0,82	0,77	0,50	0,50	0,0638	Putative ATP/GTP binding protein
SCO5539	0,51	0,56	0,92	0,62	0,48	0,0416	Uncharacterized protein
SCO5543	0,77	0,98	0,65	0,62	0,46	0,1866	Uncharacterized protein
SCO5550	0,47	0,75	1,15	0,89	0,92	0,8830	Putative transcriptional regulator
SCO5556	1,16	2,65	1,20	2,29	2,26	0,0004	Hup2, DNA-binding protein HU 2
SCO5571	0,68	1,09	0,69	0,48	0,65	0,2441	RpmF1, 50S ribosomal protein L32-1
SCO5578	1,31	1,56	1,14	3,19	2,75	0,0010	Putative sugar transporter
SCO5583	2,99	2,97	0,48	1,63	1,35	0,0012	Ammonium transporter
SCO5584	3,18	3,27	0,52	1,51	1,15	0,0008	Nitrogen regulatory protein P-II
SCO5585	1,80	2,13	0,74	1,29	1,05	0,0540	GlnD, Uridylyltransferase/uridylyl-removing enzyme
SCO5590	0,47	0,72	0,89	0,38	0,60	0,0745	Uncharacterized protein
SCO5601	0,94	0,49	1,19	0,76	0,92	0,5380	Uncharacterized protein
SCO5605	0,29	0,66	0,73	0,52	0,50	0,0109	Uncharacterized protein
SCO5609	0,30	0,68	0,45	1,74	1,23	0,0327	Uncharacterized protein
SCO5611	0,80	0,56	0,42	0,69	0,51	0,0125	Putative transcriptional regulator
SCO5614	2,07	1,62	1,13	0,88	1,32	0,1348	Putative transcriptional regulator
SCO5630	0,60	0,53	0,80	0,43	0,61	0,0327	Uncharacterized protein
SCO5640	2,07	0,52	0,75	0,67	0,66	0,9216	Uncharacterized protein
SCO5642	0,73	0,72	0,83	0,48	0,45	0,0585	Uncharacterized protein
SCO5650	0,74	0,86	0,57	0,48	0,66	0,0784	Putative membrane protein
SCO5652	1,26	1,64	1,12	1,42	2,15	0,0482	Uncharacterized protein
SCO5653	1,25	1,59	1,09	2,00	1,84	0,0128	Putative integral membrane protein
SCO5654	1,21	1,62	1,26	2,15	2,07	0,0050	ABC transporter ATP-binding protein
SCO5672	1,30	2,69	1,26	3,44	4,18	0,0000	Uncharacterized protein
SCO5724	0,39	0,68	0,63	0,34	0,36	0,0066	Uncharacterized protein
SCO5725	0,44	0,78	0,60	0,30	0,29	0,0031	ESAT-6-like protein
SCO5729	0,59	0,69	0,61	0,43	0,39	0,0135	Uncharacterized protein
SCO5741	0,46	0,87	1,13	1,15	1,30	1,1290	Putative membrane protein
SCO5758	0,49	0,79	0,83	0,80	0,95	0,4542	Putative transcriptional regulator
SCO5771	0,39	0,32	1,12	0,25	0,30	0,0087	Uncharacterized protein
SCO5772	0,32	0,36	0,84	0,32	0,34	0,0034	Uncharacterized protein
SCO5777	0,68	2,61	1,04	1,46	1,77	0,0117	Glutamate uptake system ATP-binding protein

SCO5790	1,04	1,42	1,77	1,75	1,78	0,0073	Uncharacterized protein
SCO5809	0,49	0,76	0,86	0,89	0,83	0,5532	Putative hydrolase
SCO5864	0,30	0,44	0,66	0,41	0,68	0,0035	Uncharacterized protein
SCO5877	0,75	0,50	0,83	0,40	0,30	0,0136	RedD, Transcriptional regulator RedD
SCO5880	1,03	0,71	0,94	0,47	0,44	0,0806	RedY protein
SCO5885	1,02	0,60	0,88	0,74	0,31	0,1379	Putative membrane protein
SCO5887	0,37	0,46	0,93	1,03	0,99	0,0765	Acyl carrier protein
SCO5889	1,22	0,43	0,73	0,44	0,28	0,0106	Uncharacterized protein
SCO5895	1,13	0,68	0,76	0,57	0,48	0,1493	Putative methyltransferase
SCO5898	0,71	0,82	0,84	0,51	0,46	0,1665	Probable membrane protein
SCO5907	1,16	1,54	2,34	4,23	6,33	0,0000	Uncharacterized protein
SCO5918	1,86	2,89	1,51	1,98	2,57	0,0000	Uncharacterized protein
SCO5919	2,84	2,87	1,25	1,39	1,80	0,0006	Uncharacterized protein
SCO5920	1,89	3,44	1,14	1,18	1,58	0,0014	Probable DEAD-box RNA helicase
SCO5921	0,58	1,24	0,66	0,49	0,66	0,1002	Cold-shock domain protein
SCO5936	2,22	1,27	0,95	0,99	0,97	0,4964	Uncharacterized protein
SCO5955	0,49	0,93	1,08	1,19	1,02	1,2158	Putative membrane protein
SCO5957	1,12	0,91	1,05	2,40	2,00	0,0824	Putative transport protein
SCO5961	0,59	0,50	0,78	0,94	0,88	0,1962	CbiM, Cobalt transport protein CbiM
SCO5987	1,14	2,70	1,07	1,59	2,61	0,0010	Uncharacterized protein
SCO5989	1,75	2,01	1,05	1,86	2,28	0,0058	Putative integral membrane protein
SCO5990	2,60	3,69	1,21	2,83	4,21	0,0000	Uncharacterized protein
SCO5995	0,48	0,64	0,83	0,77	0,81	0,3571	Putative secreted protein
SCO6000	0,39	0,75	0,64	0,65	0,74	0,0789	Uncharacterized protein
SCO6001	0,48	0,83	0,82	0,95	0,69	0,5177	Putative membrane protein
SCO6010	1,19	2,06	0,89	1,76	1,76	0,1038	Probable ABC-transport system ATP binding protein
SCO6014	0,91	1,49	0,93	2,55	2,55	0,0092	Putative cationic amino acid transporter
SCO6029	1,60	2,12	0,92	2,61	3,82	0,0003	Two-component regulator
SCO6045	0,93	1,20	1,20	2,20	2,66	0,0112	UPF0276 protein SCO6045
SCO6053	0,87	1,17	1,11	2,05	1,74	0,1530	Putative membrane protein
SCO6061	0,48	0,68	0,66	0,46	0,42	0,0125	Putative oxidoreductase
SCO6067	0,38	1,40	0,64	1,73	0,86	0,5430	Uncharacterized protein
SCO6079	0,43	0,71	0,89	0,60	0,75	0,1825	Uncharacterized protein
SCO6102	0,54	0,44	0,67	0,57	0,56	0,0199	Putative nitrite/sulphite reductase
SCO6124	0,99	3,01	0,67	1,38	1,40	0,0335	Uncharacterized protein
SCO6126	0,78	2,00	0,84	1,39	1,36	0,5584	Uncharacterized protein
SCO6149	1,13	1,49	0,87	1,33	2,12	0,0348	RsgA, Putative ribosome biogenesis GTPase RsgA
SCO6158	0,44	0,84	1,62	1,20	1,21	1,1132	Uncharacterized protein
SCO6162	0,64	0,72	0,86	0,45	0,46	0,0867	Putative two-component system response regulator
SCO6164	0,26	0,23	1,25	0,17	0,17	0,0000	Uncharacterized protein
SCO6165	0,53	0,41	1,04	0,38	0,34	0,0168	Uncharacterized protein
SCO6166	0,60	0,71	1,04	0,61	0,49	0,2028	Uncharacterized protein
SCO6197	0,37	0,41	0,63	0,21	0,22	0,0000	Putative secreted protein
SCO6202	1,33	1,22	1,17	2,12	2,57	0,0299	Uncharacterized protein
SCO6207	0,44	0,87	1,19	1,39	0,78	0,8833	Uncharacterized protein
SCO6241	3,57	1,27	2,00	1,36	1,27	0,0050	Uncharacterized protein
SCO6265	0,68	1,23	0,71	0,38	0,45	0,0719	Gamma-butyrolactone binding protein
SCO6276	0,53	0,58	0,57	0,32	0,58	0,0107	Putative secreted protein
SCO6277	0,74	1,03	0,79	0,47	0,96	0,6336	Putative epoxide hydrolase
SCO6279	0,54	0,70	1,58	0,50	0,95	0,2825	Putative diaminobutyrate-pyruvate aminotransferase
SCO6282	0,26	0,40	0,85	0,23	0,57	0,0030	Putative 3-oxoacyl-[acyl-carrier protein] reductase
SCO6283	0,65	0,78	1,19	0,47	0,78	0,3852	Uncharacterized protein
SCO6318	1,00	1,00	0,98	1,83	2,06	0,1297	Uncharacterized protein
SCO6323	0,60	0,58	0,73	0,50	0,45	0,0344	Putative tetR-family regulatory protein
SCO6324	1,35	2,41	0,99	2,64	3,24	0,0000	Putative hydrolase
SCO6332	0,87	0,75	1,58	3,85	2,07	0,0007	Uncharacterized protein
SCO6373	1,02	1,28	0,90	2,05	1,64	0,2021	Putative integral membrane protein
SCO6377	1,48	1,99	0,94	2,43	1,88	0,0072	Putative lipoprotein
SCO6388	2,13	0,94	0,75	1,37	1,21	0,5151	Uncharacterized protein
SCO6389	0,79	0,95	1,17	1,99	2,12	0,0597	Uncharacterized protein
SCO6429	0,66	0,51	0,82	0,41	0,34	0,0197	Uncharacterized protein
SCO6433	1,27	0,71	0,73	0,27	0,25	0,0108	Uncharacterized protein

SCO6434	1,15	0,69	0,75	0,48	0,46	0,1430	Putative oxidoreductase
SCO6451	0,79	0,49	1,12	0,70	0,61	0,2952	Putative substrate binding protein
SCO6452	0,83	0,57	0,92	0,48	0,51	0,1249	Putative transport permease protein
SCO6516	1,03	1,16	1,76	2,43	2,14	0,0096	Uncharacterized protein
SCO6533	0,49	0,89	0,94	1,23	0,94	1,1397	Putative nitrate reductase delta chain NarJ
SCO6543	1,29	1,34	0,98	1,68	2,28	0,0431	Uncharacterized protein
SCO6549	0,35	0,37	0,89	0,32	0,31	0,0063	Uncharacterized protein
SCO6624	0,34	1,16	0,52	0,42	0,63	0,0093	Putative membrane protein
SCO6631	0,47	0,80	0,63	0,47	0,45	0,0193	Putative membrane protein
SCO6650	0,46	0,90	0,43	0,45	0,55	0,0033	Uncharacterized protein
SCO6655	0,51	0,95	0,47	0,47	0,56	0,0098	RibA, GTP cyclohydrolase-2
SCO6660	1,03	1,15	1,31	1,85	2,29	0,0585	Uncharacterized protein
SCO6661	1,12	1,23	0,82	1,72	2,34	0,1001	Zwf, Glucose-6-phosphate 1-dehydrogenase (G6PD)
SCO6662	1,17	1,40	0,92	2,33	2,82	0,0153	Ttal1, Transaldolase 1
SCO6663	1,00	1,47	1,00	2,46	2,77	0,0123	Transketolase B
SCO6682	1,33	2,94	2,06	3,40	5,99	0,0000	RamS, Lanthionine-containing peptide SapB
SCO6705	1,33	1,68	1,32	2,47	2,48	0,0034	Putative secreted protein
SCO6715	1,69	4,15	0,73	0,74	1,63	0,0160	WblH, Transcriptional regulator WhiB
SCO6716	0,63	2,11	1,32	1,24	1,48	0,0432	Uncharacterized protein
SCO6727	1,34	1,73	1,20	1,50	2,70	0,0090	Uncharacterized protein
SCO6736	0,40	0,42	0,85	0,38	0,28	0,0076	Putative metallopeptidase
SCO6761	0,42	0,89	1,45	1,55	1,99	1,2241	Uncharacterized protein
SCO6797	0,64	0,77	0,57	0,48	0,50	0,0372	Putative ATP/GTP binding protein
SCO6808	0,48	0,69	0,57	0,42	0,44	0,0083	Putative ArsR-family transcriptional regulator
SCO6821	0,59	0,72	2,13	1,60	0,77	0,8419	Putative transferase
SCO6823	0,48	0,78	0,97	0,49	0,95	0,2338	Putative ArsR-family transcriptional regulator
SCO6828	0,59	0,57	0,62	0,49	0,46	0,0078	Putative secreted protein
SCO6831	2,16	1,14	0,94	1,23	1,00	0,3292	Uncharacterized protein
SCO6933	1,00	1,17	0,89	2,02	2,17	0,0453	Uncharacterized protein
SCO6946	1,98	1,85	2,62	1,60	2,69	0,0000	Uncharacterized protein
SCO7008	2,31	2,08	1,12	0,97	1,08	0,0663	EcaA, Putative ABC-transport protein
SCO7029	1,68	2,14	0,71	1,67	1,47	0,0377	Possible binding-protein-dependent transport protein
SCO7067	2,01	0,92	0,81	1,51	1,14	0,5822	Uncharacterized protein
SCO7071	1,07	1,44	1,32	1,83	2,10	0,0313	Uncharacterized protein
SCO7072	1,29	1,84	1,37	2,13	2,15	0,0073	Uncharacterized protein
SCO7106	0,18	0,35	1,35	3,10	2,49	0,0536	WblJ, Transcriptional regulator WhiB
SCO7122	0,75	0,43	0,94	0,87	1,14	0,4424	Putative acetyltransferase
SCO7133	2,27	0,72	0,99	1,17	1,33	0,2874	Putative membrane protein
SCO7153	1,28	1,72	1,15	3,27	2,97	0,0006	Putative sugar transporter
SCO7154	0,50	0,99	0,75	0,56	0,68	0,2816	IlvC2, Ketol-acid reductoisomerase 2
SCO7178	2,03	1,21	0,49	1,53	1,07	0,4326	Putative transcriptional regulatory protein
SCO7184	0,90	1,18	0,44	0,95	1,12	0,9282	Putative branched amino acid transport system permease
SCO7189	1,31	0,49	1,01	0,82	0,64	0,2567	Uncharacterized protein
SCO7190	1,48	1,02	0,91	0,80	0,48	0,7647	Prenyltransferase homolog (Uncharacterized protein)
SCO7232	0,62	0,92	0,98	1,83	2,24	0,2775	Putative secreted protein
SCO7251	0,48	0,62	0,87	0,51	0,60	0,0660	Uncharacterized protein
SCO7277	1,44	1,31	1,06	2,00	2,60	0,0124	Putative regulator protein
SCO7306	0,60	1,09	0,55	0,34	0,53	0,0109	WblK, Transcriptional regulator WhiB
SCO7312	0,41	0,85	1,05	1,17	1,08	1,0835	Uncharacterized protein
SCO7333	1,49	1,69	1,12	2,20	2,54	0,0061	Uncharacterized protein
SCO7335	1,08	1,28	0,98	1,97	2,04	0,0583	GlgE2, Alpha-1,4-glucan:maltose-1P maltosyltransferase 2
SCO7336	2,21	2,58	0,86	3,46	3,78	0,0000	Uncharacterized protein
SCO7337	0,78	1,25	1,11	1,89	2,12	0,0960	Uncharacterized protein
SCO7363	1,38	1,27	1,09	2,34	2,19	0,0249	Putative oxidoreductase
SCO7365	1,56	2,17	1,39	2,80	3,53	0,0005	Uncharacterized protein
SCO7383	1,60	2,00	1,20	1,74	2,56	0,0042	Uncharacterized protein
SCO7385	0,49	0,90	1,16	1,08	0,95	1,1546	Putative integral membrane protein
SCO7392	2,83	1,33	1,03	1,25	1,41	0,0825	Uncharacterized protein
SCO7426	1,71	2,31	1,49	1,48	2,55	0,0006	Uncharacterized protein
SCO7427	0,39	0,70	1,56	1,51	1,46	0,8849	NsrR, HTH-type transcriptional repressor NsrR
SCO7434	1,49	3,90	0,87	4,41	5,82	0,0000	Putative lipoprotein
SCO7449	0,92	3,16	1,43	5,75	5,70	0,0000	Putative membrane protein

SCO7450	1,25	1,42	1,55	2,17	2,45	0,0033	Putative secreted protein
SCO7451	0,81	1,59	1,13	1,96	2,31	0,0434	Uncharacterized protein
SCO7452	1,15	3,68	0,93	3,42	4,59	0,0000	Putative O-methyltransferase
SCO7453	2,44	6,65	1,41	5,91	8,96	0,0000	Putative secreted protein
SCO7471	1,87	2,23	0,67	1,37	1,58	0,0156	Putative phenylacetic acid degradation protein PaaA
SCO7472	2,56	2,45	0,90	1,62	1,91	0,0052	Putative phenylacetic acid degradation protein PaaB
SCO7476	1,42	1,22	1,44	1,83	2,01	0,0261	Putative secreted protein
SCO7481	0,43	0,85	0,75	0,50	0,44	0,0327	Uncharacterized protein
SCO7514	0,83	0,86	1,51	1,83	2,20	0,0610	Putative integral membrane protein
SCO7524	0,42	1,50	1,36	1,62	2,08	1,2143	Uncharacterized protein
SCO7528	0,96	0,61	2,03	1,50	1,73	0,1307	Uncharacterized protein
SCO7536	0,71	0,47	0,76	0,43	0,35	0,0051	Putative integral membrane protein
SCO7555	2,09	1,36	1,09	1,27	1,39	0,1978	Putative solute binding lipoprotein
SCO7598	1,75	1,40	1,40	1,35	2,10	0,0098	Uncharacterized protein
SCO7613	1,08	2,00	1,35	1,57	1,78	0,0124	Putative integral membrane protein
SCO7657	0,92	0,93	0,82	0,62	0,47	0,3515	Putative secreted protein
SCO7658	0,66	0,74	0,63	0,47	0,44	0,0306	Uncharacterized protein
SCO7660	1,15	1,38	1,48	1,99	2,15	0,0236	KcsA, pH-gated potassium channel KcsA
SCO7668	0,45	0,85	1,04	0,99	0,93	1,0071	Uncharacterized protein
SCO7673	1,35	1,81	1,28	2,35	2,95	0,0009	Putative lipoprotein
SCO7676	0,21	0,11	2,16	0,16	0,48	0,0000	Putative ferredoxin
SCO7677	0,19	0,20	1,22	0,23	0,25	0,0006	Putative secreted solute-binding protein
SCO7678	0,23	0,28	1,12	0,42	0,46	0,0029	Putative metal transport integral membrane protein
SCO7679	0,42	0,50	0,93	0,50	0,53	0,0418	Putative transport system integral membrane protein
SCO7681	0,51	0,49	1,10	0,49	0,45	0,0562	Putative AMP-binding ligase
SCO7682	0,38	0,42	0,96	0,37	0,37	0,0136	Putative non-ribosomal peptide synthase
SCO7683	0,31	0,26	0,96	0,26	0,25	0,0016	Putative non-ribosomal peptide synthase
SCO7684	0,36	0,39	1,13	0,28	0,32	0,0107	Uncharacterized protein
SCO7685	0,36	0,45	0,94	0,42	0,39	0,0118	Uncharacterized protein
SCO7686	0,38	0,35	0,92	0,35	0,33	0,0087	Putative cytochrome P450
SCO7687	0,29	0,31	0,92	0,29	0,27	0,0015	Putative thioesterase
SCO7688	0,38	0,22	1,06	0,19	0,18	0,0008	Uncharacterized protein
SCO7689	0,44	0,43	0,96	0,33	0,36	0,0162	Putative ABC transporter ATP-binding protein
SCO7690	0,43	0,48	0,96	0,49	0,46	0,0258	Putative ABC transporter ATP-binding protein
SCO7691	0,37	0,40	1,09	0,41	0,40	0,0188	Putative lyase
SCO7692	0,68	0,49	1,04	0,46	0,43	0,0759	Uncharacterized protein
SCO7696	0,48	0,42	0,80	1,20	1,32	0,1168	Putative marR-family transcriptional regulator
SCO7752	0,49	0,51	2,00	1,48	1,63	0,6721	Putative integral membrane protein
SCO7755	0,66	0,48	0,71	0,28	0,41	0,0044	Uncharacterized protein
SCO7776	2,60	0,98	1,20	1,38	1,37	0,1349	Uncharacterized protein
SCO7788	1,84	2,16	1,17	1,40	2,23	0,0033	Putative membrane protein
SCO7793	2,15	1,34	1,47	1,33	1,09	0,0875	Uncharacterized protein
SCO7799	1,67	0,97	1,19	1,50	2,14	0,0708	Putative transposase
SCO7800	1,55	1,32	2,77	1,08	1,14	0,0448	Uncharacterized protein
SCO7804	0,74	1,38	1,66	1,87	2,79	0,0089	Putative membrane protein
SCO7807	2,31	1,53	0,75	1,34	1,49	0,0814	Uncharacterized protein

* Fold change: *osdR* mutant over wild-type ratio

\# SCO refers to *Streptomyces coelicolor* genome database
