# Supplementary material for: OsdR of Streptomyces coelicolor and the Dormancy Regulator DevR of Mycobacterium tuberculosis Control Overlapping Regulons
Source: mSystems. 2016 May 3;1(3):e00014-16. doi: 10.1128/mSystems.00014-16 (PMC5069765; doi:10.1128/mSystems.00014-16)
Supplement: Table S5 [file sys001162018st5.pdf]

Supplementary Table S5: Overview genes deregulated at 36 hours.

SCO #	M145						*osdR* Mutant						Protein annotation
	24h	30h	36h	42h	54h	Profile	24h	30h	36h	42h	54h	Profile	
SCO0008	1,60	1,15	1,00	1,00	0,86		2,71	2,11	2,43	1,07	1,95		Uncharacterized protein
SCO0038	2,07	2,69	7,42	3,17	3,22		1,35	2,88	2,93	2,65	2,14		Putative sigma factor
SCO0117	0,71	0,65	1,55	0,62	0,98		0,95	0,59	0,50	0,60	0,54		Putative oxidoreductase
SCO0128	1,35	1,22	0,55	0,88	0,92		1,04	1,33	1,36	1,00	1,36		Uncharacterized protein
SCO0162	0,86	0,62	1,31	0,73	0,58		0,88	0,46	0,59	0,55	0,70		Uncharacterized protein
SCO0167	0,94	0,86	1,87	0,95	0,70		0,67	0,56	0,60	0,61	0,61		Usp-domain protein
SCO0168	1,60	1,55	5,76	1,57	1,17		0,78	0,66	0,57	0,84	1,05		Possible regulator protein
SCO0169	1,13	1,07	4,06	1,30	0,92		0,78	0,46	0,53	0,48	0,51		Uncharacterized protein
SCO0170	0,84	0,89	1,84	1,01	0,91		0,95	0,74	0,81	0,66	0,71		Uncharacterized protein
SCO0174	1,28	0,97	1,79	1,01	0,86		0,95	0,69	0,77	0,86	1,11		Putative DNA-binding protein
SCO0177	0,73	0,61	0,97	0,63	0,54		0,70	0,40	0,38	0,40	0,44		Putative membrane protein SCJ1.26
SCO0181	1,40	1,21	1,91	1,14	0,97		1,28	0,53	0,74	0,65	0,91		Usp-domain protein
SCO0185	1,18	2,26	7,72	3,22	2,13		3,04	7,64	7,15	6,90	4,89		Geranylgeranyl pyrophosphate synthase
SCO0186	1,21	1,74	5,81	2,42	1,90		2,64	6,31	6,24	6,50	5,34		Phytoene dehydrogenase
SCO0188	0,73	1,11	2,76	1,29	1,08		1,64	3,81	3,61	4,16	3,13		Putative methylesterase
SCO0191	0,67	1,11	3,97	1,79	2,04		1,87	3,39	3,52	3,81	3,04		Putative lycopene cyclase
SCO0199	0,92	0,93	2,30	0,99	0,75		0,87	0,73	0,73	0,64	0,69		Putative alcohol dehydrogenase
SCO0200	1,06	1,05	2,58	1,24	0,91		0,72	0,64	0,64	0,59	0,59		UspA
SCO0201	0,66	0,70	1,38	0,81	0,63		0,42	0,33	0,41	0,41	0,40		Putative integral membrane protein
SCO0204	1,23	1,58	3,32	1,54	1,10		-	-	-	-	-		OsdR
SCO0214	0,70	0,74	1,57	0,92	0,76		1,02	0,39	0,51	0,42	0,56		Uncharacterized protein
SCO0216	1,19	1,16	2,36	0,97	0,85		0,85	0,76	0,73	0,85	0,80		NarG2
SCO0217	0,96	1,01	1,65	0,84	0,76		0,88	0,81	0,72	0,74	0,77		NarH2
SCO0218	0,86	0,90	1,75	0,87	0,73		0,59	0,65	0,57	0,70	0,73		NarJ2
SCO0219	0,63	0,64	1,40	0,60	0,47		0,63	0,34	0,34	0,39	0,39		NarI2
SCO0223	2,85	2,52	3,18	1,93	2,31		2,69	3,82	1,52	3,12	3,58		Uncharacterized protein
SCO0415	1,56	1,17	0,60	0,79	0,54		0,99	0,93	1,28	1,28	1,54		Putative membrane protei
SCO0436	23,00	27,54	2,65	10,07	11,78		6,77	6,07	6,03	5,79	4,96		RpmF2
SCO0472	5,35	4,22	0,55	4,99	4,03		0,81	0,45	0,51	0,52	0,52		Putative secreted protein
SCO0473	2,46	2,86	0,41	2,91	2,42		0,67	0,43	0,46	0,38	0,43		Putative solute-binding lipoprotein
SCO0474	3,56	2,81	0,65	3,55	3,07		0,89	0,60	0,66	0,65	0,66		Putative lipoprotein
SCO0475	2,19	2,68	0,44	2,57	2,24		0,66	0,35	0,42	0,44	0,43		ABC transporter protein
SCO0476	2,38	2,46	0,39	2,18	2,01		0,76	0,31	0,39	0,40	0,55		ABC transport protein
SCO0477	3,40	3,01	0,98	4,09	3,99		1,38	1,01	1,10	1,13	1,29		Uncharacterized protein
SCO0530	0,76	0,70	1,15	0,71	0,76		0,74	0,79	0,54	0,70	0,70		Putative transcriptional regulator
SCO0570	9,82	9,10	1,40	11,52	12,17		0,76	1,06	1,00	1,56	1,31		RpmG2
SCO0743	2,44	3,01	7,56	3,44	3,56		4,10	6,89	5,93	6,37	8,02		Putative secreted protein
SCO0876	0,46	0,40	0,52	0,34	0,39		0,32	0,41	0,21	0,39	0,40		Uncharacterized protein
SCO0931	2,39	2,50	4,82	2,38	2,66		1,81	2,16	2,40	2,37	2,78		Putative secreted proline-rich protein
SCO0934	0,34	0,56	6,64	1,52	1,74		0,74	4,31	10,18	13,98	19,79		Putative integral membrane protein
SCO0939	0,46	0,47	1,28	0,58	0,72		0,63	0,44	0,50	0,60	0,64		Putative hydrolase
SCO1089	1,50	1,97	4,52	1,92	1,73		2,40	4,16	4,03	4,19	4,47		Uncharacterized protein
SCO1105	2,31	2,29	3,26	1,63	1,56		1,13	2,25	1,42	1,27	0,94		Putative secreted protein
SCO1235	1,52	2,04	6,16	2,21	1,70		2,44	4,42	2,92	2,01	1,72		UreB
SCO1236	1,63	2,32	6,41	2,11	1,71		2,68	4,53	2,73	1,94	1,43		UreA, Urease subunit gamma
SCO1415	0,68	1,05	4,69	2,31	3,05		0,78	2,71	4,82	7,94	10,57		SmeA
SCO1541	1,03	1,53	8,15	3,91	4,45		1,46	5,28	9,39	17,69	30,14		SsgB
SCO1550	15,81	7,49	3,70	17,11	21,47		5,57	2,39	3,31	7,73	14,47		Putative small membrane protein
SCO1571	3,75	4,64	5,44	4,48	4,50		2,05	3,79	2,63	2,30	2,61		Uncharacterized protein
SCO2008	8,03	11,86	11,68	6,68	3,85		4,44	9,87	4,99	2,50	1,43		Branched chain amino acid binding protein
SCO2009	1,84	2,24	2,34	1,43	1,18		1,16	2,03	1,12	0,95	0,75		Branched chain amino acid transport permease
SCO2113	1,39	2,00	10,19	4,53	5,17		2,27	6,62	7,71	6,80	7,30		Bfr
SCO2195	4,03	3,83	10,55	7,14	5,68		3,15	4,59	4,45	4,12	3,39		Uncharacterized protein
SCO2210	3,14	3,54	18,31	2,74	1,74		8,84	12,57	7,91	5,28	2,62		GlnII
SCO2211	3,03	3,26	8,48	3,11	1,98		7,61	9,38	7,05	5,22	2,73		Uncharacterized protein
SCO2212	5,64	6,10	7,56	9,91	9,01		2,02	3,27	3,10	2,81	1,97		Uncharacterized protein
SCO2217	25,10	25,36	25,23	12,57	4,40		13,12	22,18	10,49	3,58	1,42		Putative secreted protein
SCO2486	2,40	3,59	7,68	2,43	1,17		4,79	8,93	5,07	4,17	1,55		NirB2
SCO2487	1,41	2,08	8,95	1,75	0,85		3,21	7,56	4,23	2,93	0,92		NirB
SCO2488	1,69	2,12	6,15	1,30	0,76		2,76	6,90	4,12	3,20	1,66		NirD
SCO2505	5,83	6,12	1,00	5,94	5,11		0,81	0,73	0,76	0,76	0,66		ZnuA
SCO2506	3,20	3,41	1,22	4,01	3,84		0,72	1,01	0,95	0,96	0,95		ZnuC
SCO2507	2,70	2,77	1,06	2,97	2,73		0,87	0,91	0,97	0,81	0,79		ZnuB
SCO2574	3,80	4,03	5,45	4,43	3,74		1,50	2,86	2,22	1,95	1,52		Uncharacterized protein
SCO2580	2,08	2,62	1,13	2,58	2,58		0,65	1,16	1,13	1,13	1,17		Putative membrane protein
SCO2629	1,04	0,81	0,31	1,23	1,05		0,57	0,57	1,09	1,76	1,19		Putative membrane protein
SCO2633	1,39	2,02	0,89	3,52	7,21		0,76	0,89	0,88	0,92	2,91		SodF
SCO2634	5,60	4,72	1,76	4,77	5,18		3,27	3,18	2,02	1,68	2,02		Uncharacterized protein
SCO2705	3,46	8,08	20,61	7,60	6,72		6,48	19,16	26,89	26,79	30,48		ChpF
SCO2716	2,17	5,20	20,18	5,78	4,88		6,81	19,19	26,02	27,67	37,46		ChpA
SCO2718	19,34	54,70	184,00	63,32	43,44		69,99	142,88	166,79	142,48	269,61		RdlA
SCO2719	7,87	21,62	63,24	26,46	19,87		20,21	45,87	49,83	43,03	74,30		RdlB
SCO2774	1,21	1,14	7,01	2,67	1,69		1,92	2,92	6,22	4,57	2,63		Acyl-CoA dehydrogenase
SCO2779	0,77	0,73	1,79	0,81	0,87		1,03	0,68	2,22	1,69	1,53		Acyl-CoA dehydrogenase
SCO2795	0,89	0,70	2,10	0,98	0,93		1,60	1,67	1,87	1,71	2,11		Putative sugar binding secreted protein
SCO2819	4,35	3,62	1,76	5,15	5,50		1,65	1,51	1,53	1,48	1,95		Uncharacterized protein

SCO2924	0,75	1,06	5,66	2,10	1,82		1,39	3,87	6,29	8,22	8,85		SsgG
SCO2959	1,28	1,56	4,22	1,15	0,51		2,37	4,54	1,65	1,30	0,56		Putative nitrate extrusion protein
SCO3051	1,32	1,61	7,51	3,53	2,82		1,90	3,69	6,46	4,82	4,26		Acyl-CoA dehydrogenase
SCO3097	2,50	2,65	6,39	2,60	2,41		2,13	3,33	5,22	3,20	2,91		RpfA
SCO3105	2,45	1,86	6,62	3,15	4,41		2,12	1,73	2,88	2,76	1,63		Uncharacterized protein
SCO3106	2,36	1,85	5,05	2,56	2,18		2,14	1,68	2,38	2,33	1,37		Putative lipoprotein
SCO3121	2,70	1,74	0,70	1,49	1,50		0,57	0,62	0,66	0,84	0,76		Uncharacterized protein
SCO3206	1,59	2,37	0,54	2,68	3,91		0,71	0,71	0,61	0,53	0,63		Putative transmembrane efflux protein
SCO3207	3,08	4,18	1,24	5,55	9,06		1,18	1,12	1,19	1,18	1,58		TetR-family regulator (Fragment)
SCO3254	0,57	0,54	1,25	0,62	0,65		0,55	1,02	0,44	0,67	0,64		SpdC protein
SCO3309	3,87	3,38	2,07	2,26	2,16		3,58	3,96	4,21	2,65	3,41		Uncharacterized protein
SCO3379	0,86	1,03	3,07	1,46	1,55		0,96	2,09	2,62	2,97	3,03		Uncharacterized protein
SCO3425	2,05	2,18	0,62	2,24	2,72		0,32	0,61	0,53	0,68	0,66		RpsR2, 30S ribosomal protein S18 2
SCO3426	4,08	3,78	0,85	4,52	4,75		0,68	0,73	0,78	0,82	0,82		Uncharacterized protein
SCO3427	7,60	5,39	0,37	7,31	6,93		0,65	0,46	0,47	0,47	0,45		RpmE2, 50S ribosomal protein L31 type B 2
SCO3428	6,42	5,89	1,13	9,99	10,55		0,64	0,72	0,74	0,85	0,79		RpmG1 , 50S ribosomal protein L33 1
SCO3429	3,33	2,58	0,57	2,87	2,87		0,65	0,58	0,66	0,57	0,68		RpmB2, 50S ribosomal protein L28-2
SCO3430	4,38	3,40	0,36	3,61	3,46		1,15	0,49	0,53	0,47	0,60		RpsN2
SCO3431	5,10	4,19	1,18	5,14	4,77		1,28	1,17	1,31	1,13	1,15		Possible membrane protein
SCO3437	0,89	1,39	0,53	1,55	1,90		0,60	0,77	0,65	0,65	0,65		Putative integral membrane protein
SCO3438	0,71	1,34	0,37	1,66	2,23		0,66	0,57	0,46	0,40	0,38		Uncharacterized protein
SCO3449	1,14	2,72	1,25	2,67	2,92		0,99	2,50	1,22	1,05	0,91		Uncharacterized protein
SCO3472	8,80	8,42	4,61	8,35	6,39		12,25	15,40	15,13	10,84	24,42		Putative transposase remnant
SCO3473	9,60	11,17	8,79	15,06	10,80		3,46	0,86	1,74	1,18	1,32		Putative aldolase
SCO3474	11,06	15,77	11,46	23,30	16,28		2,01	0,50	0,84	0,56	0,96		Putative sugar kinase
SCO3475	15,58	20,51	17,65	30,78	21,93		3,77	1,01	1,40	1,20	1,15		Putative isomerase
SCO3476	7,91	10,11	7,07	13,18	10,45		2,05	1,16	1,11	1,08	1,21		Putative short-chain dehydrogenase
SCO3477	6,66	10,17	7,50	11,51	9,44		2,82	2,28	2,54	2,10	2,08		Putative dehydrogenase
SCO3529	0,53	0,38	0,19	0,36	0,31		0,78	0,34	0,42	0,44	0,44		Uncharacterized protein
SCO3537	2,07	2,57	3,22	4,55	4,74		1,28	1,57	1,61	1,41	1,76		Putative DNA-binding protein
SCO3538	2,85	3,04	4,79	6,29	7,52		1,58	1,82	1,99	1,95	2,75		Uncharacterized protein
SCO3668	5,33	5,78	10,01	7,55	9,23		2,74	4,21	4,14	5,65	6,75		HspR
SCO3671	9,20	11,67	32,31	20,36	21,19		5,92	9,31	11,58	18,34	22,85		DnaK
SCO3701	1,19	1,39	0,50	1,03	1,26		1,01	1,19	1,44	0,70	1,11		Uncharacterized protein
SCO3800	1,11	1,23	6,52	2,96	2,35		1,65	2,74	5,71	5,87	4,89		Putative acyl-CoA dehydrogenase
SCO3863	0,65	1,49	19,25	4,92	5,19		1,43	11,67	19,75	29,97	33,86		Putative secreted protein
SCO3870	2,65	2,34	2,85	4,32	5,54		1,24	1,39	1,39	1,59	1,67		Putative integral membrane protein
SCO3945	1,18	1,24	4,14	1,86	1,84		1,32	1,53	2,21	3,18	2,91		CydA
SCO4032	1,42	1,20	4,55	2,54	3,53		1,52	0,86	1,76	3,97	5,62		Putative MarR regulatory protein
SCO4142	0,49	0,66	1,86	0,49	0,58		0,84	1,29	0,61	0,52	0,47		Phosphate-binding protein PstS
SCO4178	7,59	7,77	13,92	13,80	15,79		3,46	6,16	6,58	7,16	8,68		Putative small membrane protein
SCO4200	3,23	3,31	7,11	6,14	7,41		1,33	2,43	3,41	4,30	5,12		Putative membrane protein
SCO4214	1,54	1,51	0,49	1,74	2,09		0,49	0,40	0,37	0,38	0,35		Putative AbaA-like regulatory protein
SCO4295	18,50	15,52	29,80	27,85	21,05		7,46	10,65	13,08	11,14	11,43		Cold shock protein
SCO4616	0,22	0,26	1,49	0,38	0,77		0,24	0,21	0,19	0,22	0,26		Excisionase
SCO4619	0,60	0,64	1,47	0,83	1,16		0,58	0,63	0,62	0,69	0,67		Putative integral membrane protein
SCO4626	0,52	0,51	1,10	0,59	0,88		0,73	0,51	0,49	0,44	0,62		Uncharacterized protein
SCO4640	1,50	1,06	1,92	1,03	1,11		2,14	1,14	0,93	0,86	0,94		Putative tetR-family transcriptional regulator
SCO4642	0,62	0,42	0,72	0,37	0,34		0,87	0,35	0,26	0,30	0,26		Uncharacterized protein
SCO4679	3,48	3,94	4,56	6,47	6,87		1,34	2,02	2,18	1,85	2,02		Uncharacterized protein
SCO4706	26,53	34,77	63,29	67,92	63,40		15,01	23,63	27,66	23,85	31,89		RpsS, 30S ribosomal protein S19
SCO4707	33,55	39,68	86,07	79,03	61,16		24,79	44,08	40,57	30,39	37,43		RplV, 50S ribosomal protein L22
SCO4761	47,69	25,72	49,13	48,61	60,16		30,83	16,17	23,60	16,66	29,53		GroES
SCO4789	4,44	4,52	4,83	3,17	1,93		2,13	3,32	2,06	1,54	1,00		Putative integral membrane protein
SCO4858	4,60	6,43	13,25	9,31	8,81		2,87	5,70	5,82	5,39	6,37		Succinate dehydrogenase subunit
SCO5032	2,43	1,34	4,72	1,94	2,21		4,13	3,05	2,42	2,08	2,27		AhpC
SCO5071	0,66	0,75	0,20	0,57	0,96		0,85	0,43	0,37	0,30	0,33		Hydroxylacyl-CoA dehydrogenase
SCO5074	1,10	1,18	0,44	1,29	2,24		1,72	0,93	0,54	0,48	0,44		Putative dehydratase
SCO5089	0,63	0,52	0,15	0,60	1,32		1,34	0,38	0,24	0,18	0,19		Actinorhodin acyl carrier protein (ACP) (actI ORF3)
SCO5123	10,38	7,01	13,38	14,70	14,84		3,00	5,03	5,91	9,22	15,86		Putative small membrane protein
SCO5245	4,14	3,47	4,36	4,68	5,72		2,23	2,49	2,14	2,56	2,65		UshY, Putative uncharacterized protein ushY
SCO5249	3,16	2,02	12,73	5,85	8,12		4,58	3,26	9,71	10,35	17,99		Putative nucleotide-binding protein
SCO5314	0,85	0,93	3,69	1,41	1,96		0,92	2,19	4,88	4,21	9,32		WhiE
SCO5315	0,35	0,53	5,80	1,47	2,09		0,60	1,84	5,30	6,00	13,54		WhiE
SCO5319	1,24	1,45	5,35	2,18	2,52		1,50	3,71	5,94	5,30	7,19		WhiE
SCO5389	5,24	5,00	8,66	5,04	4,65		3,33	7,62	3,89	2,79	3,28		Uncharacterized protein
SCO5439	0,83	1,51	3,12	1,22	0,86		0,82	3,16	2,41	1,54	0,94		DNA helicase (EC 3.6.4.12)
SCO5461	1,12	1,47	6,80	2,74	3,01		1,20	3,21	4,95	4,86	5,12		Putative secreted protein
SCO5484	1,96	1,08	13,60	3,81	3,79		3,84	1,98	5,06	11,54	13,42		Small hydrophobic membrane protein
SCO5521	3,61	5,74	9,56	8,85	11,08		3,30	4,54	4,62	6,01	9,43		Uncharacterized protein
SCO5583	2,23	2,65	13,98	2,86	1,91		6,69	7,87	6,73	4,66	2,59		AmtB
SCO5584	2,79	3,18	18,88	3,96	2,30		8,87	10,40	9,85	5,95	2,65		GlnK
SCO5585	1,52	1,46	3,69	1,59	1,08		2,74	3,11	2,72	2,05	1,13		GlnD
SCO5609	4,87	5,07	4,53	2,63	3,18		1,47	3,44	2,06	4,58	3,90		Uncharacterized protein
SCO5611	0,50	0,41	0,43	0,48	0,58		0,40	0,23	0,18	0,33	0,29		Putative transcriptional regulator
SCO5672	1,19	1,36	5,12	2,48	2,22		1,55	3,66	6,45	8,53	9,26		Uncharacterized protein
SCO5771	1,96	2,14	0,62	2,78	2,92		0,76	0,68	0,69	0,70	0,88		Uncharacterized protein
SCO5772	3,56	3,56	1,36	3,86	3,92		1,12	1,28	1,14	1,22	1,34		Uncharacterized protein
SCO5907	2,11	1,32	0,67	0,55	0,50		2,44	2,04	1,58	2,35	3,15		Uncharacterized protein
SCO5990	0,54	0,50	2,04	0,86	0,80		1,40	1,83	2,48	2,45	3,37		Uncharacterized protein

SCO6010	0,65	0,51	1,22	0,60	0,67		0,77	1,05	1,08	1,06	1,18		Probable ABC-transport system ATP binding protein
SCO6029	1,37	2,50	7,60	3,30	3,44		2,19	5,30	6,98	8,63	13,12		WhiI
SCO6164	2,30	1,44	0,33	2,03	1,70		0,61	0,33	0,41	0,34	0,29		Uncharacterized protein
SCO6165	1,04	0,73	0,31	0,98	1,16		0,56	0,30	0,32	0,37	0,39		Uncharacterized protein
SCO6241	0,25	0,29	0,21	0,26	0,29		0,89	0,37	0,42	0,36	0,37		Uncharacterized protein
SCO6279	1,53	1,11	0,49	1,61	0,85		0,83	0,78	0,78	0,80	0,81		Putative diaminobutyrate-pyruvate aminotransferase
SCO6324	0,50	0,69	3,28	1,52	2,05		0,67	1,66	3,23	4,00	6,65		Putative hydrolase
SCO6549	1,98	1,94	0,91	2,49	2,67		0,70	0,72	0,81	0,81	0,83		Uncharacterized protein
SCO6650	15,62	18,23	19,95	9,69	2,97		7,20	16,45	8,53	4,35	1,62		Uncharacterized protein
SCO6655	9,41	9,01	9,59	5,07	1,93		4,77	8,60	4,47	2,38	1,08		RibA, GTP cyclohydrolase-2
SCO6682	7,84	3,11	2,94	1,69	1,29		10,40	9,15	6,05	5,74	7,72		RamS
SCO6821	1,29	0,97	0,47	0,92	1,33		0,76	0,70	0,99	1,46	1,02		Putative transferase
SCO6946	0,87	0,79	0,60	0,63	0,47		1,72	1,47	1,58	1,01	1,27		Uncharacterized protein
SCO7178	0,34	0,23	0,36	0,22	0,26		0,69	0,28	0,18	0,33	0,28		Putative transcriptional regulatory protein
SCO7184	0,59	0,53	0,79	0,48	0,49		0,53	0,63	0,35	0,46	0,55		Branched amino acid transport system permease
SCO7336	0,19	0,24	1,44	0,54	0,80		0,43	0,61	1,24	1,86	3,04		Uncharacterized protein
SCO7434	0,47	0,68	8,27	2,63	3,73		0,70	2,67	7,17	11,59	21,72		Putative lipoprotein
SCO7449	1,13	1,43	10,69	3,88	6,31		1,05	4,53	15,26	22,29	35,96		WhiE-like
SCO7452	1,35	1,89	9,75	3,95	4,68		1,55	6,95	9,06	13,51	21,52		WhiE-like
SCO7453	0,74	1,35	9,22	3,38	3,97		1,80	8,96	13,01	19,96	35,55		WhiE-like
SCO7471	0,51	0,61	2,18	1,02	1,22		0,95	1,36	1,45	1,40	1,93		Putative phenylacetic acid degradation protein PaaA
SCO7472	0,75	0,78	2,20	1,00	1,07		1,93	1,91	1,98	1,63	2,04		Putative phenylacetic acid degradation protein PaaB
SCO7528	2,04	2,25	0,87	1,16	1,13		1,94	1,37	1,77	1,74	1,97		Uncharacterized protein
SCO7676	15,62	23,82	1,62	13,34	9,53		3,34	2,59	3,50	2,20	4,54		Coelibactin
SCO7677	4,56	4,17	0,71	4,27	4,02		0,85	0,83	0,87	1,00	1,02		Coelibactin
SCO7678	3,28	2,96	0,65	2,13	1,94		0,77	0,84	0,73	0,89	0,89		Coelibactin
SCO7681	1,48	1,41	0,58	1,36	1,45		0,75	0,69	0,64	0,67	0,65		Coelibactin
SCO7682	1,76	1,92	0,80	2,19	2,20		0,66	0,81	0,77	0,81	0,82		Coelibactin
SCO7683	2,44	2,38	0,66	2,73	2,85		0,75	0,61	0,63	0,72	0,73		Coelibactin
SCO7684	2,28	2,09	0,73	2,59	2,46		0,82	0,82	0,83	0,73	0,80		Coelibactin
SCO7685	2,20	2,51	1,15	2,70	2,76		0,79	1,14	1,08	1,14	1,08		Coelibactin
SCO7686	1,46	1,59	0,61	1,76	1,81		0,55	0,56	0,56	0,62	0,61		Coelibactin
SCO7687	3,04	3,16	0,99	3,82	3,71		0,89	0,98	0,91	1,09	1,02		Coelibactin
SCO7688	1,91	2,22	0,45	2,56	2,27		0,72	0,49	0,48	0,48	0,42		Coelibactin
SCO7689	1,35	1,53	0,64	1,74	1,72		0,60	0,66	0,61	0,58	0,62		Coelibactin
SCO7690	2,58	2,70	1,28	2,87	2,91		1,11	1,29	1,23	1,42	1,33		Coelibactin
SCO7691	1,93	2,05	0,72	2,23	2,21		0,71	0,82	0,79	0,90	0,89		Coelibactin
SCO7692	1,39	1,41	0,66	1,60	1,75		0,95	0,69	0,69	0,73	0,75		Coelibactin
SCO7752	2,63	1,95	0,85	1,43	1,28		1,29	1,00	1,70	2,11	2,09		Putative integral membrane protein
SCO7800	0,38	0,28	0,17	0,24	0,23		0,59	0,37	0,46	0,26	0,26		Uncharacterized protein
